# MolDeTr: A Chemistry-Informed Deep Learning Model for Next-Generation Automated Analysis of ^1^H NMR Spectra

**DOI:** 10.1021/acs.analchem.5c03465

**Published:** 2026-08-04

**Authors:** Nicolas Schmid, Marc Wanner, Giulia Fischetti, Andreas Henrici, Mohsen Meshkian, Simon Bruderer, Rudolf M. Füchslin, Björn Heitmann, Jan Dirk Wegner, Roland K. O. Sigel, Dirk Wilhelm

**Affiliations:** † Institute of Applied Mathematics and Physics (IAMP), Zurich University of Applied Sciences (ZHAW), Technikumstrasse 71, 8401 Winterthur, Switzerland; ‡ Department of Mathematical Modeling and Machine Learning (DM3L), 27217 University of Zurich (UZH), Winterthurerstrasse 190, 8057 Zurich, Switzerland; § Department of Chemistry, 27217 University of Zurich (UZH), Winterthurerstrasse 190, 8057 Zurich, Switzerland; ∥ Computer Vision Laboratory (CVL), ETH Zurich, Sternwartstrasse 7, 8092 Zurich, Switzerland; ⊥ Ca’ Foscari University of Venice, Dorsoduro 3246, 30123 Venice, Italy; # 206144 Bruker Switzerland AG, Industriestrasse 26, 8117 Fällanden, Switzerland; ¶ European Centre for Living Technology (ECLT), Ca’ Bottacin, Dorsoduro 3911, Calle Crosera 30123, Venice, Italy

## Abstract

Accurate interpretation of one-dimensional proton nuclear magnetic resonance (^1^H NMR) spectra remains a rate-limiting step in molecular structure elucidation, particularly when signal overlap, strong spin coupling, and instrumental distortions mask key features. Existing automated approaches depend on computationally intensive and sensitive iterative quantum-mechanical fitting and still require expert oversight. Here we introduce MolDeTr, a chemistry-informed deep-learning framework derived from the detection-transformer architecture that unifies peak picking, multiplet identification, and extraction of chemical shifts, scalar coupling constants, relaxation-dependent decay times, and proton counts in a single-network pass. The method targets prototypical spin systems of single-component small molecules in 1D ^1^H NMR, with up to ten distinct groups of chemically equivalent spins (multiplets). MolDeTr is trained exclusively on synthetic spectra generated by spin-dynamics simulations and augmented with realistic experimental artifacts, enabling it to generalize to unseen compounds—including experimental spectra with overlapping and strongly coupled multiplets—without reference standards or prior spin-system knowledge. Unlike structure-conditioned shift-prediction or calculation models, e.g., density functional theory (DFT), that assume the molecular structure is known, MolDeTr addresses the spectrum-conditioned inverse problem and extracts spin-system parameters directly from measured 1D ^1^H NMR spectra, thereby substantially improving chemical-shift prediction precision by one to 2 orders of magnitude compared to existing structure-conditioned approaches. Benchmarking against a diverse experimental set of ^1^H NMR spectra of modestly sized small molecules, spanning 80 to 600 MHz base frequency, shows median absolute errors of 0.89 Hz for chemical shifts and 0.20 Hz for coupling constants, while absolute proton counts are predicted with 93.5% accuracy, outperforming state-of-the-art spectrum analysis software and experienced spectroscopists. By eliminating iterative fitting and expert intervention, MolDeTr offers a scalable route to fully automated spectral analysis, accelerating molecular discovery across the chemical sciences.

## Graphical abstract

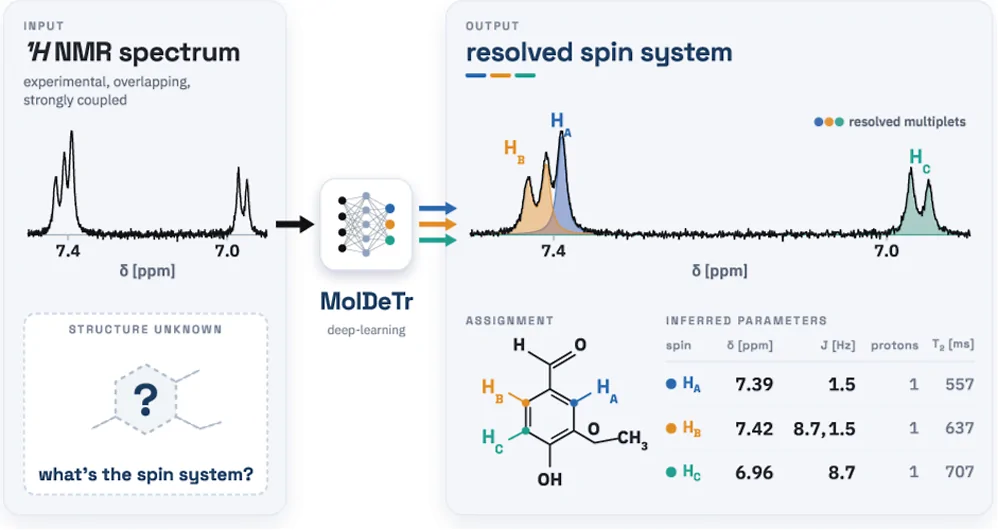

## Introduction

Spectral data analysis is critical to characterizing and understanding molecular structures, dynamics, and interactions. Among the various spectral techniques, nuclear magnetic resonance (NMR) spectroscopy stands out as a fundamental tool in molecular science. NMR spectra provide detailed insight into molecular properties by analyzing spin systems, groups of atomic nuclei whose quantum spin states generate distinctive signals. This capability is especially critical in one-dimensional (1D) proton NMR spectroscopy, a pivotal tool in fields such as drug discovery, metabolomics, and materials science, due to its rapid data acquisition and rich informational output.^1–3^

Although the simulation of spin systems (the forward problem) has attained high precision—albeit often at considerable computational cost—the interpretation of NMR spectra (the inverse problem) remains challenging and continues to be a major bottleneck in the field. Analyzing these spectra requires extensive expertise and time, as analysts must meticulously deconstruct each signal within its spectral context. Complex or distorted signal patterns exacerbate this challenge, necessitating laborious iterations between spin system simulations and comparisons with experimental data. This manual approach is time-consuming, costly, and prone to variability and errors,^4^ which limits the scalability of NMR analysis in high-throughput environments such as metabolomics and drug discovery. Although expert interpretation can achieve high accuracy using prior knowledge of spin systems, there is a pressing need for technological advancements to improve efficiency and gain deeper insights.^5^

Semiautomated simulation-based techniques have been developed to assist in inferring spin systems from NMR spectra by leveraging expert knowledge. Analysts must input the spin system structure and often specify spectral regions and approximate parameter ranges, integrating manual expertise with computational tools. While these methods enhance the interpretation process, their reliance on prior knowledge and manual intervention can limit applicability and scalability.^6–11^

In parallel, traditional automation methods such as peak picking and coupling constant extraction have been used to mitigate the challenges in NMR analysis.^12–20^ Although these automated approaches effectively handle routine aspects of spectral interpretation, they often struggle with challenging spectra featuring low signal-to-noise ratios (SNR) , spectral distortions, overlapping signals, and strong coupling interactions, which can lead to misinterpretations.^21^

Recognizing these limitations, recent advances in deep learning have made significant strides in NMR analysis. Innovations in this domain include artifact removal,^22^ peak picking,^23^ deconvolution,^24,25^ and multiplet detection.^26–28^ Further methods have also facilitated mixture analysis,^29–32^ nonuniform sampling (NUS) reconstruction,^33,34^ prediction of molecular structures^35–37^ and automated protein structure elucidation.^38^ A substantial body of prior work predicts NMR chemical shifts directly from molecular structures, spanning classical ML, deep learning, and hybrid DFT + ML approaches.^39–46^ These structure-conditioned models can reach high accuracy for small molecules when the molecular graph (and often conformational information) is available, typically achieving mean absolute errors of 0.05–0.15 ppm (30–90 Hz at 600 MHz base frequency) for ^1^H chemical shifts and 0.5–1 Hz for coupling constants. Despite these advances, accurately interpreting 1D proton NMR spectra, a fundamental technique for small molecule analysis, remains challenging, especially in low-field environments where overlapping signals and strong coupling obscure a clear interpretation.^21,47,48^

To address the challenges of interpreting complex spectral data, we developed the molecular detection transformer (MolDeTr), an end-to-end deep learning framework inspired by the detection transformer (DeTr) architecture and customized for spectral data analysis and molecular property elucidation.^49,50^ MolDeTr translates intricate spectral signatures into molecular representations, demonstrating its effectiveness with 1D proton nuclear magnetic resonance spectroscopy, renowned for its interpretative complexity. In 1D proton NMR spectra, signals from hydrogen nuclei typically appear as distinct patterns of peaks called multiplets, e.g., singlets, doublets, triplets, and quartets, arising from spin–spin interactions between neighboring hydrogen atoms in weakly coupled systems. However, in strongly coupled systems, these patterns become distorted due to increased interdependence among spins, leading to signal intensity, position, and symmetry alterations that deviate from the expected simple patterns.

MolDeTr bridges spectral data with meaningful molecular properties by identifying key relationships within and across spectral and molecular domains. Its underlying transformer architecture learns relational dependencies between entities in a sequence: in molecular spectroscopy, this means capturing connections between spin groups whose signals interact through J-coupling and chemical-shift proximity. Figure 1a illustrates this workflow for a substituted propene sample, and Figure 1b demonstrates MolDeTr’s performance on a complex aromatic spin system. The ill-posed nature of spectral inversion and its implications are treated explicitly in the Methods section. For a conceptual illustration of how relational attention compares across language processing and spectral analysis, see Supporting Information Section 1.5.

**Figure 1:**
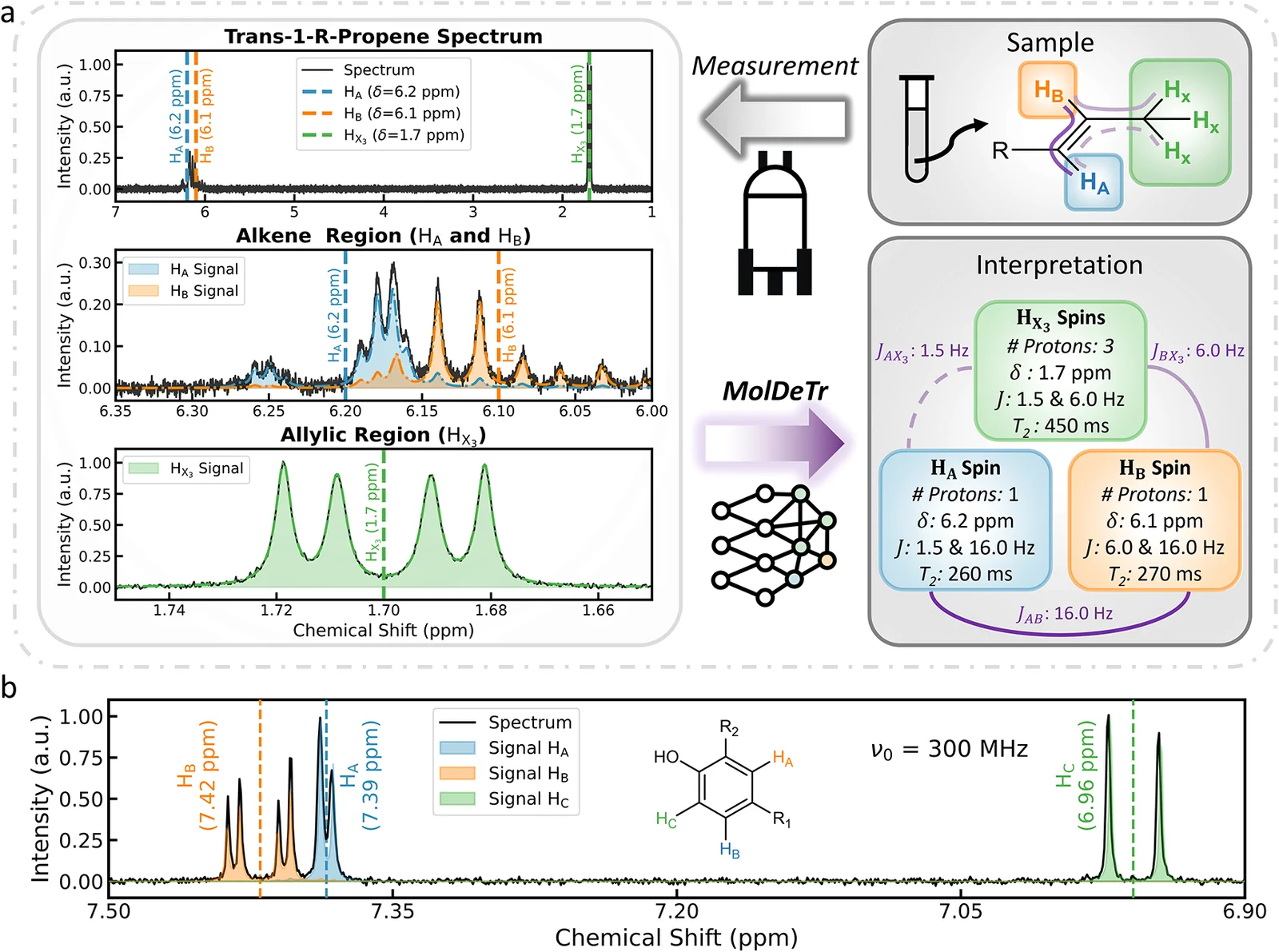
(a) MolDeTr’s automated NMR interpretation workflow. The workflow begins with measuring a trans-configured 1-substituted propene (R-CH=CH-CH_3_) sample using an NMR spectrometer, producing a one-dimensional proton spectrum enriched with complex resonance patterns. MolDeTr then automatically interprets this raw spectral data by identifying and classifying sets of magnetically equivalent spins. In this example, the molecule, selected spectral regions, and derived outputs are color-coded to highlight distinct spin groups (H_A_, H_B_, and H_X_3__). Each annotated resonance is associated with its key parameters—proton count, chemical shift δ, coupling constants *J*, and transverse relaxation times (*T*_2_)—thereby facilitating the direct elucidation of overlapping, strongly coupled spin systems. (b) Aromatic region of vanillin. Experimental proton NMR spectrum of vanillin measured at 300 MHz, highlighting the aromatic region. The full spectrum (black line) is overlaid with contributions from individual spins (H_A_, H_B_, and H_C_), indicated by vertical dashed lines at their corresponding chemical shifts. This strongly coupled ABC spin system challenges conventional spectral analysis methods, illustrating the type of spectral complexity, including strong coupling, signal overlap, and field distortions, that MolDeTr is designed to resolve (see Table 2a for quantitative results).

MolDeTr advances the interpretation of spectral data by utilizing deep learning based on detection transformers to analyze complex molecular spectra. The method targets prototypical spin systems of single-component small molecules in 1D ^1^H NMR, with up to ten distinct groups of chemically equivalent spins (multiplets). It is trained on highly representative data derived from quantum mechanical spin system simulations enhanced with realistic experimental distortions. Thus, MolDeTr effectively interprets complex experimental NMR spectra, as illustrated in Figure 1a for a substituted propene sample. By encoding chemical information within its architecture and loss functions, MolDeTr learns nuanced contextual and relational cues present in both spectral and molecular data. As a next-generation framework, it combines deep learning innovations with chemistry-informed principles and synthetic training data that approach experimental reality, enabling unprecedented capabilities in spectral analysis. This end-to-end approach directly links NMR spectra to information on molecular structures and dynamics, predicting key parameters of the spin system, including chemical shifts δ, proton counts, transverse relaxation times *T*_2_, and coupling constants *J*.

MolDeTr demonstrates clear superiority over state-of-the-art methods in coherently interpreting challenging spectra. It does not require prior knowledge of the sample or its spin systems, nor does it rely on reference standards for absolute proton count estimation. As such, MolDeTr’s contribution is orthogonal to structure-conditioned shift and *J*-coupling predictors, e.g., DFT, HOSE codes^51^ or data-driven structure to NMR predictors. In terms of chemical-shift precision, MolDeTr substantially outperforms recently proposed structure-conditioned workflows by one to 2 orders of magnitude.^39,40,52^ Under the latter conditions, it enables the interpretation of 1D proton NMR spectra previously inaccessible to expert and algorithmic analysis, possibly reducing the need for more resource-intensive two-dimensional (2D) NMR experiments in these cases and addressing previously unsolved challenges in spectral interpretation.

Furthermore, MolDeTr’s flexible framework demonstrates the potential for applications beyond NMR spectroscopy.^48^ This cross-disciplinary innovation represents a significant milestone in analytical and computational chemistry,^53,54^ enhancing research capabilities and paving the way for automated high-throughput spectral analysis in fields such as drug discovery, natural products, and metabolomics.

To illustrate how MolDeTr serves chemical analysis in practice, we describe a representative workflow. An unknown, isolated small molecule, for instance a reaction byproduct identified during pharmaceutical process development, a purified natural product fraction, or an unidentified substance in forensic analysis, is measured by standard 1D ^1^H NMR spectroscopy, optionally complemented by elemental composition from high-resolution mass spectrometry. From the crowded, potentially overlap-dominated spectrum, MolDeTr extracts an interpretable spin-system parametrization: chemical shifts (δ), coupling constants (*J*), line width proxies (Γ), and absolute proton counts, even in the presence of strong coupling, signal overlap, and field distortions. These parameters are immediately actionable: they constrain coupling topology and substitution patterns, narrow the space of plausible substructures, and enable rapid candidate ranking through consistency checks and spin-simulation matching. As demonstrated for vanillin (Figure 1b and Table 2), MolDeTr resolves complex aromatic spin systems that established commercial software cannot disentangle, directly providing the quantitative parameters needed to confirm or rule out candidate structures without prior knowledge of the spin system. This end-to-end-capability, from raw spectrum to structural constraints, is directly applicable to pharmaceutical impurity profiling, natural product identification, forensic substance characterization, process analytical technology on benchtop NMR instruments, and quality control without reference standards.

## Methods

### Chemistry-Informed Deep Learning for Solving Ill-Posed Inverse Problems in Molecular Analysis

Molecular characterization underpins progress across the life sciences and chemistry, with spectroscopic techniques such as NMR, IR, and MS providing essential molecular parameters. However, interpreting these spectra typically involves addressing ill-posed inverse problems caused by overlapping features, noise, and other experimental artifacts.^48,55^

In spectral analysis, such inverse problems are ill-posed when one attempts to infer the molecular system from an observed spectrum. Three primary challenges, which can be interconnected, include:

- Nonuniqueness: multiple molecular structures may yield indistinguishable or nearly identical spectral signatures, complicating unambiguous assignment of molecular parameters.
- Instability: small perturbations (e.g., noise, baseline shifts) in the recorded spectrum can lead to disproportionately large deviations in inferred molecular parameters, undermining reproducibility and reliability.
- Incompleteness: the information contained in a single spectrum may be insufficient to fully characterize a spin system or molecule, necessitating additional constraints or complementary data.

Although these phenomena are conceptually distinct, they often compound one another. For example, incomplete data (lack of sufficient spectral coverage or resolution) can produce nonunique solutions and intensify instability. Conversely, instability introduced by noise can mask critical spectral features, effectively reducing the informational content and thereby mimicking incompleteness.

To address these challenges, we consider a neural network *N* that approximates the inverse mapping from the observed spectrum *S* to the underlying spin system _*X*_

N(S)≈X

The training objective inverts the forward model *S* = *D*(*F*(*X*)), where *F* represents an ideal spin-system simulator and *D* encapsulates distortions and noise (Figure 2a). By exploiting large data sets and incorporating supplementary constraints, this neural network approach can learn to disentangle complex overlapping signals, thereby improving the robustness of molecular interpretations despite the ill-posed nature of the inverse problem.

**Figure 2:**
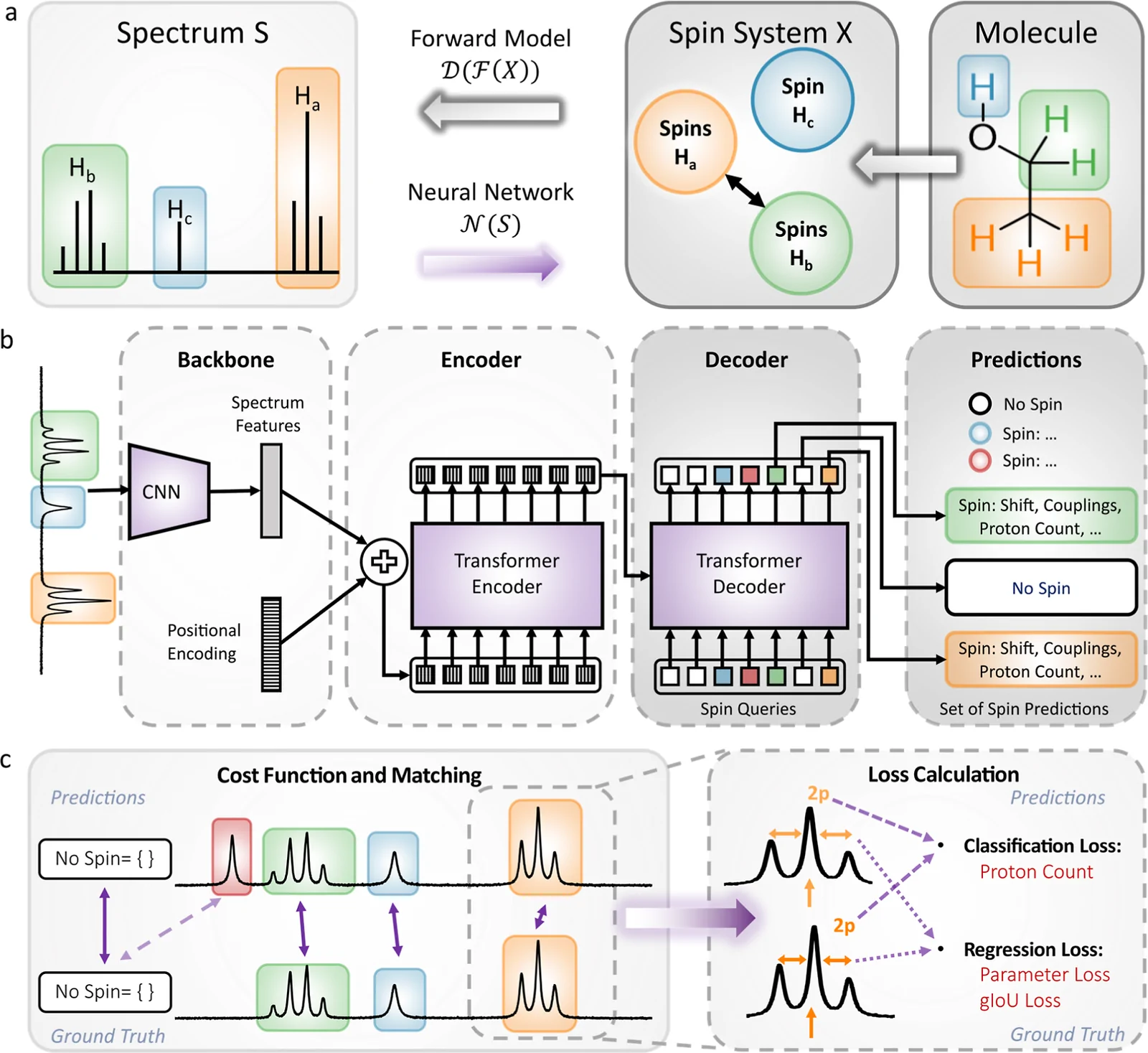
(a) Schematic representation of the inverse problem in molecular spectral analysis using ethanol’s spin system *X* in 1D proton NMR spectroscopy. The molecule comprises three distinct proton environments: methyl protons (H_a_), methylene protons (H_b_), and hydroxyl protons (H_c_). Interpreting relational cues, such as spin–spin coupling between H_a_ and H_b_, alongside contextual cues like characteristic chemical shift regions, facilitates the retrieval of *X* from the observed spectrum *S* via a neural network *N*. (b) Schematic of MolDeTr for molecular spectral analysis. Backbone: a 1D FPN extracts features from the spectrum, which are positionally encoded. Encoder: the transformer models relationships across spectral regions using self-attention, capturing relational and contextual information. Decoder: via cross-attention, encoded features link with spin query representations to generate spin parameter embeddings. Predictions: the network outputs spin/atom-wise characteristics such as proton counts, chemical shifts, coupling constants, and transverse relaxation times. (c) Bipartite matching and loss calculation in MolDeTr. Left: predicted parameters are matched to ground truth according to a cost function; colored boxes indicate matched entities; violet arrows show matches. Right: loss calculations are performed for matched predictions and ground truth parameters. Dotted arrows denote classification losses (e.g., proton counts), and dashed arrows indicate regression losses (e.g., chemical shifts and coupling constants).

A robust approach to spectral analysis leverages both relational and contextual information, moving beyond the isolated inspection of individual peaks. Traditional bottom-up peak-picking pipelines, though computationally convenient, can overlook interconnections among spectral and molecular features, often leading to misinterpretation or inconsistent assignments.^25^ By contrast, incorporating relational and contextual cues substantially enhances the accuracy and reliability of elucidating molecular properties, including structural organization, intermolecular interactions, and dynamic behaviors.

Relational cues capture how spectral features inform on molecular connectivity, spatial arrangement, and potential interactions. Key examples include:

- Spin–spin couplings (NMR): coupling constants (*J*-values) reveal which nuclei are bonded or in close spatial proximity. Karplus relationships link these values to dihedral angles, thereby providing insights into three-dimensional conformations and local dynamics.
- Fragmentation patterns (MS): characteristic ion fragments reflect the bonding arrangement and functional group connectivity within a molecule, offering clues into both static and reactive molecular states.
- Vibrational coupling (IR/Raman): overlapping vibrational bands (e.g., Fermi resonances) highlight interactions among functional groups, often manifested through peak shifting, splitting, or broadening.

Contextual cues situate observed spectral features within a broader experimental and environmental framework, enhancing interpretative power:

- Characteristic frequency ranges: functional groups typically exhibit signals in known regions—for instance, carbonyl stretches at ∼1700–1750 cm^–1^ (IR), π–π* transitions in UV/vis, or distinct chemical shifts for carbonyl carbons in NMR.
- Environmental influences: solvent polarity, temperature, and pH can significantly shift peak positions or alter intensities (e.g., pH-dependent shifts near p*K*_a_ values), necessitating careful contextual interpretation.
- Instrumentation and calibration: field strength, detector sensitivity, and referencing standards (e.g., TMS in NMR, polystyrene in IR) directly affect observed spectra. Parameters like resolution and scan rate must be standardized across instruments to ensure comparability.

/wtb/By integrating these relational and contextual cues, spectral analysis transcends narrow, peak-centric perspectives to yield a holistic, reproducible understanding of molecular structures, interactions, and dynamics. This comprehensive view mitigates ambiguous assignments, strengthens structural and mechanistic hypotheses, and unifies data interpretation across diverse experimental platforms.

In 1D proton NMR spectroscopy, the challenge of resolving inverse problems is especially pronounced for complex spin systems, where overlapping signals and subtle coupling patterns frequently surpass the capabilities of classical peak-picking methods. By integrating robust molecular constraints and leveraging domain knowledge, our approach delivers both higher accuracy and deeper insight into molecular structures and dynamics.

**Chemistry-Informed Representation Learning**:

- Magnetically equivalent spins: we reduce the parameter space by grouping magnetically equivalent protons, ensuring chemical plausibility in model predictions.
- MolDeTr architecture: drawing on concepts from transformer-based object detection, MolDeTr captures the relational and contextual nuances of spectral and molecular data from 1D proton NMR, refer to next Section for more details on MolDeTr’s architecture.
- Loss functions: domain-specific knowledge is embedded directly into the loss functions, mapping observed spectral features to spins (nodes) within graph-like molecular representations. Thus, coupling networks, chemical shifts, and other molecular constraints are integrated into the core model optimization process, refer to Section on matching and loss functions.

**Leveraging Relational and Contextual Information**:

- Relational analysis: by tracking spin–spin coupling patterns and their magnitudes, our model elucidates molecular connectivity and stereochemical arrangements, refining traditional analysis of bond topology.
- Contextual analysis: chemical shift regions are used to infer local electronic environments and identify key functional groups. These contextual insights constrain the range of plausible structural motifs and reinforce model consistency across diverse molecular classes.

This top-down, chemistry-informed pipeline surpasses conventional peak-picking-based heuristics by embedding spectral and molecular principles at every data processing stage. For additional details, see Supporting Information Sections 1.2 and 1.3, where we further elaborate on the role of relational and contextual cues in 1D proton NMR and demonstrate how domain-specific constraints unify robust model training with interpretability in challenging analytical scenarios.

### MolDeTr: A Detection Transformer-Based Framework Delivering Unprecedented Interpretative Power in Molecular Analysis

Spectral methods are pivotal in molecular science for elucidating molecular structures, dynamics, and interactions, but traditional neural networks often fail to extract detailed information from difficult spectra and overlapping signals. To address this limitation, we present MolDeTr, inspired by the detection transformer (DeTr).^50^ Designed explicitly for spectral and molecular data analysis, MolDeTr leverages detection transformer models to capture the relationships between spectral features and spin systems, enabling precise prediction of magnetically equivalent spin parameters directly from the NMR spectra. An overview of the MolDeTr architecture is shown in Figure 2b.

Inputs are phase- and baseline-corrected 1D spectra resampled to 6144 points across 1200 Hz (5.12 points/Hz). The network outputs an orderless set of equivalent-spin entities, each comprising proton count, chemical shift, line width Γ (∝ 1/*T*_2_), a 1D signal interval, and a permutation-invariant *J*-descriptor *E*(*C*).

The MolDeTr architecture comprises three primary components: a backbone network, a transformer module, and a prediction network. The backbone employs a one-dimensional feature pyramid network (FPN),^56^ adapted to extract multiscale features from 1D proton NMR spectra. This network generates rich spectral embeddings that encapsulate essential information about the spectrum, providing a robust foundation for subsequent processing.

The extracted embeddings are augmented with positional encodings, adding important chemical shift information to capture local and global spectral information. We use one-dimensional, learnable positional embeddings^49^ to encode chemical shifts along the spectral axis (see Supporting Information Section 2.1). The Transformer encoder utilizes self-attention mechanisms to dynamically model relationships across different spectral regions, enabling MolDeTr to adapt to various spectral patterns and regions. Unlike convolutional neural networks (CNNs), which rely on fixed-size filters and often struggle with long-range dependencies, the self-attention mechanism allows MolDeTr to flexibly adjust to relevant spectral features regardless of their position. This capability is advantageous for analyzing difficult spectra, including both close- and far-apart resonances (see Supporting Information Section 1.4 for visualizations of MolDeTr’s use of relationships between spectral patterns). Architectural specifics of the transformer configuration and attention mechanism are provided in Supporting Information Section 2.1. In brief, we adopt multiscale deformable attention from Zhu et al.,^57^ adapted from two-dimensional spatial features to one-dimensional spectral features. The model uses 6 encoder and 6 decoder layers with 8-head attention (4 feature levels, 4 sampling points per level), a hidden dimension of 256, and a 1D FPN backbone with 11 residual blocks (Supporting Information Section 2.1).

In the Transformer decoder, self-attention is first applied to the learnable queries, representing equivalent spins in NMR or, more generally, atoms or spectral groups. This self-attention mechanism allows queries to diversify their representations, enabling them to focus on different molecular aspects, such as distinct spins or specific atomic environments. Following this, cross-attention aligns the encoded spectral features with each specialized query, allowing MolDeTr to target each spin’s most relevant spectrum aspects. Subsequently, another self-attention layer models the interactions among these spin queries, capturing molecular dependencies between spins such as connectivity, bonding, stoichiometry, stereochemistry, and molecular dynamics. Thus, MolDeTr effectively emulates the behavior of higher-dimensional NMR experiments such as total correlation spectroscopy (TOCSY). This sequential application of self-attention and cross-attention enables MolDeTr to effectively interpret both isolated and interconnected features of difficult spectra and molecules, thereby enabling a comprehensive mapping between spectral and molecular domains.

The prediction network processes the Transformer’s output to generate parameters related to equivalent-spin groups. By default, we group magnetically equivalent protons. In symmetric aromatic motifs (e.g., AA^′^BB^′^)

We represent chemically equivalent but magnetically nonequivalent positions as a single entity with multiple *J* entries. These parameters include both discrete properties (e.g., proton counts in NMR) and continuous properties (e.g., chemical shifts, coupling constants, signal regions, and transverse relaxation times). For inference, a single decoder query group of ten spin-query slots is used (see Supporting Information Section 2.1.2 for the Group-DETR training strategy; “query groups” here refers to the decoder training strategy and is distinct from chemically equivalent spin groups). This comprehensive output enables direct, end-to-end extraction of molecular information from NMR spectra, removing the need for extensive postprocessing or the complex, fragile pipelines of independent heuristics common in traditional methods.

MolDeTr employs chemistry-informed detection transformer architectures to model relationships between spectral features and spins or atoms, thereby enhancing the coherence and capabilities of spectral analysis and improving molecular characterization and structure elucidation. Detailed architectural and implementation specifics of MolDeTr are provided in Section 2.1.

### Optimized Spectroscopy-Specific Matching and Loss Functions Drive Enhanced Spectral Analysis

To adapt MolDeTr for molecular spectral analysis, we introduced an NMR-specific cost and loss function tailored to 1D proton NMR data. This approach directly aligns predicted and ground truth spins, as illustrated in Figure 2c. Using a set prediction framework,^50^ MolDeTr naturally accommodates varying numbers of spectral entities (e.g., spins, atoms, or groups) without requiring postprocessing steps such as nonmaximum suppression. Instead, it employs the Hungarian algorithm^58^ to optimally match predicted spins with their true counterparts. This strategy enables MolDeTr to handle spectra containing varying numbers of multiplets (groups of magnetically equivalent spins), including those that share similar or identical chemical shifts. By predicting a fixed number of spins and utilizing a “no spin” category for excess predictions, the model maintains robust generalization across diverse spin systems.

The cost function is comprised of three equally weighted components, balancing classification and regression tasks:

- Proton count cost (classification cost): this component penalizes incorrect predictions of proton counts and misclassifications between “spin” and “no spin” classes, utilizing focal loss.^59^ It is specifically tuned to address class imbalances and focuses on challenging cases.
- Continuous spin parameter cost (*L* ^1^ cost): this term computes the absolute differences between predicted and true values for key NMR spectral parameters, such as chemical shifts and coupling constants. It ensures accurate numerical predictions of these essential NMR features.
- Signal overlap cost (gIoU cost): generalized intersection over union (gIoU) cost^60^ is applied to 1D signal regions in the NMR spectrum, measuring the overlap between predicted and actual signal regions to refine boundary predictions. Notable overlapping signal regions are not a problem for MolDeTr, as illustrated in Figure 3.

**Figure 3:**
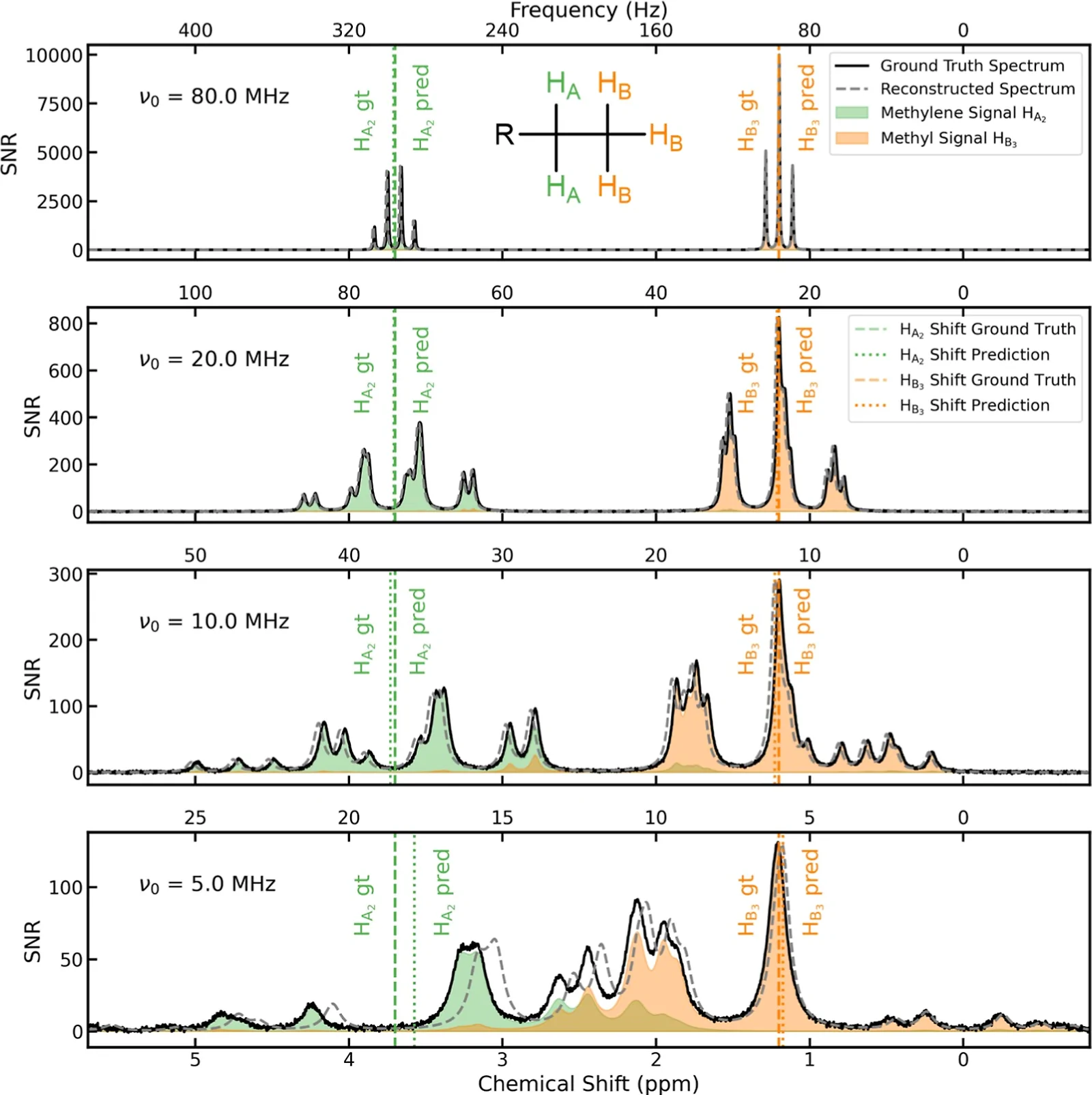
MolDeTr accurately reconstructs ethyl group spectra across different magnetic field strengths. Spectra of the ethyl group at varying base frequencies: 80 MHz, 20, 10, and 5 MHz. In each panel, the ground truth spectrum is shown as a solid black line, and the reconstructed spectrum simulated from MolDeTr’s predicted parameters is depicted as a dashed gray line. The colored areas represent the individual contributions from the spin groups: green for methylene protons (H_A_2__) and orange for methyl protons (H_B_). Vertical dashed lines indicate the ground truth chemical shifts, while vertical dotted lines denote the predicted chemical shifts, with colors corresponding to each group. The close alignment between the ground truth and reconstructed spectra across all field strengths demonstrates MolDeTr’s accuracy and robustness in handling spectral conditions, including strong coupling effects and low SNR conditions.

After matching the predicted spins with the ground truth, MolDeTr employs a corresponding loss function to guide the training (see Figure 2c). The loss function mirrors the structure of the cost function and encloses the following:

- Proton count loss (classification loss): this is applied to all predictions to ensure accurate proton count predictions and proper differentiation between “spin” and “no spin” classes.
- Continuous spin parameter loss (*L* ^1^ and gIoU loss): this combined loss, applied only to matched spins (when proton count > 0), refines the prediction of chemical shifts, coupling constants, transverse relaxation times, and signal regions, thereby improving regression accuracy.

Furthermore, MolDeTr incorporates efficient training and resampling strategies, allowing it to scale across varying spectral widths and base frequencies. By standardizing the digital resolution of the spectrum and leveraging MolDeTr’s robust architecture, the model maintains consistent performance across different NMR instruments and spectral ranges. Detailed information on the optimizer, training parameters, loss weighting, normalization, learning rate strategies, and computational resources is available in Supporting Information Section 2.3.

Our specialized cost and loss functions, along with tailored training methods, introduce chemical constraints, enabling adaptability and coherent analysis across various spectral and molecular conditions.

### Advancing Synthetic Data Generation: Combining Simulation with Experimental Spectral Distortions

Synthetic data generation has become indispensable in spectral analyses, with NMR spectroscopy serving as a prominent example.^5,21^ This strategy is particularly valuable for training deep learning algorithms, as it mitigates the difficulties of acquiring consistently labeled experimental data, which is often expensive and labor-intensive. Additionally, the demand for large and diverse data sets, which usually cannot be feasibly obtained through experimental methods alone, highlights the critical role of synthetic data generation in advancing spectral research.

Conventional methods for generating synthetic spectral data usually depend on the random sampling of key spectral parameters, including peak positions, intensities, line widths, and shapes.^21^ While efficient, these methods frequently overlook crucial molecular and spectral contexts—such as the spin coupling relationships within a molecule, spectral regions tied to specific functional groups, and higher-order quantum effects (strong coupling). These elements are fundamental in NMR for capturing the connectivity patterns unique to real molecular environments. Machine learning models lacking such interconnected and contextual chemical specificity may struggle with experimental spectral data, where capturing these nuanced relationships is key.

To overcome some of these limitations in the case of NMR spectroscopy, we introduce a novel methodology that combines quantum mechanical spin system simulation with realistic spectral distortions. By simulating spin systems frequently found in molecular motifs in small molecules, our approach captures the complexity of multiplets and the intricate coupling interactions between spins, providing a more accurate foundation for training deep learning models in spectral analysis. Specifically, our methodology simulates various proton spin systems classified by their coupling strengths: uncoupled, weakly coupled, and strongly coupled. These categories include methyl groups (A_3_), polysubstituted aromatic systems (AX and AMX), ethyl groups (A_2_X_3_), and complex aromatic frameworks (AA′XX′ and AA′BB′C). A comprehensive list of spin systems and detailed examples are provided in Supporting Information Section 3.1.

Our simulation process for data generation involves constructing Hamiltonians based on selected spin systems and their corresponding parameters. We then solve the stationary Schrödinger equation to determine the spin system’s eigenvalues and eigenstates and calculate the resonance frequencies and intensities.^61,62^ Leveraging the methodology described by De Lorenzi et al.,^63^ we efficiently simulate millions of spin systems, making this approach highly scalable for large-scale synthetic data generation while maintaining high accuracy. Quantum mechanical simulations of spin systems provide precise representations within experimental accuracy.^8^ This precision enables the generation of distortion-free synthetic spectra that faithfully capture the complex behavior of molecular spin systems. Detailed simulation parameters, including ranges for chemical shifts, coupling constants, transverse relaxation times, and computation techniques, are provided in Supporting Information Section 3.2. Additionally, annotated examples of the generated spectra are available in Supporting Information Section 3.2.1. This comprehensive parameter space ensures the simulations capture a broad spectrum of molecular scenarios, providing highly realistic and robust training data for MolDeTr.

Under ideal conditions, simulated and experimental spectra would be virtually indistinguishable if all common distortions and artifacts were perfectly corrected and the SNR was sufficiently high. However, achieving such comprehensive correction in practice is rarely feasible. Therefore, we deliberately introduce realistic artifacts and distortions into our simulated spectra to bridge the gap between simulation and real-world experimentation. Although our spin system simulations successfully replicate the intrinsic signals observed in experimental data, they inherently lack the imperfections commonly encountered in actual NMR measurements. We address this gap by incorporating ^13^C satellites, field inhomogeneities (modeled using SHIMpanzee^64^), phase distortions, Gaussian noise at varying intensities, and baseline distortions. Examples of these artificially introduced imperfections are provided in Supporting Information Section 3.2.2.

To ensure reproducibility and to cover edge cases across different instruments and processing pipelines, we specify the numeric ranges used for randomization. Asymmetric line broadening was varied between 0 and 3 Hz (fwhm, shim-induced). Phase errors included both zero-order distortions of up to ± 8° and first-order distortions of ± (8/1200)° Hz^–1^ across the 1200 Hz window. ^13^C satellites were introduced with coupling constants *J*_CH_ = 40–220 Hz and intensities ranging from 0.5% to 1.5% of the parent line. Noise levels were adjusted so that the maximum peak (SNR_max_) spanned 10^2^ to 10^5^. Residual baseline distortions were added with amplitudes up to 5× the noise level. These wide ranges were deliberately chosen to mirror cross-laboratory variability, including differences in consoles, probes, processing pipelines, and sample preparation, and to ensure robust model generalization across heterogeneous acquisition settings.

By merging high-fidelity quantum mechanical simulations of spin systems with realistic spectral distortions, our approach establishes a new standard for generating synthetic NMR data. This framework yields accurately labeled molecular spin systems and spectra that more closely mirror experimental observations, optimizing machine learning model training. Moreover, it bridges theoretical simulations with real-world applications, fostering enhanced analytical accuracy and reliability across various scientific fields (see Supporting Information Section 3.3). This strategy of incorporating spectral distortions alongside precise simulations has the potential to inspire similar methodologies across other spectral techniques beyond NMR.

## Results

### Exceptional Performance in Crowded and Distorted Simulated Spectra

To rigorously evaluate the effectiveness of MolDeTr in the spectral analysis of small molecules using 1D proton NMR, we first tested it on a comprehensive and high-quality simulated data set comprising 10,000 spectra with the corresponding ground truth. This data set was designed to reflect realistic molecular scenarios in proton NMR spectroscopy, covering a variety of cases, including spectra with multiple independent spin systems—some exhibiting overlap and strong coupling, which are common challenges in analyzing small-molecule spectra. To emulate real-world spectral distortions and enhance the algorithm’s robustness, these spin systems were exposed to various distortions, including field inhomogeneities, residual phase errors, and noise (see the Methods Section for details on data generation and distortions). In spectra with lower SNR in the test set, some individual resonance peaks, the distinct signals that make up multiplets, can fall below the noise level because of their low intensity.

We evaluated MolDeTr for several parameters, including chemical shifts, coupling constants, line widths, and signal regions, as well as its classification accuracy in determining the number of protons per magnetically equivalent spin signal. The results, summarized in Table 1a, demonstrate MolDeTr’s high predictive accuracy, achieving strong coefficients of determination (*R* ^2^) and low absolute errors for both chemical shifts and coupling constants. This enables detailed structural and dynamic insights from a single 1D proton NMR spectrum.

**Table 1:** Overview of Parameter Prediction Performance across Different Setups^a^

	Δ	*J*
(a) Multiple spin systems		
*R* ^2^	0.996	0.979
MAE [Hz]	3.73	0.56
MedAE [Hz]	0.53	0.17
Acc. [%]	99.1	
Acc. (excl.) [%]	97.5	
(b) Single weakly coupled spin systems		
*R* ^2^	0.999	0.987
MAE [Hz]	0.49	0.31
MedAE [Hz]	0.35	0.11
Acc. [%]	99.6	
Acc. (excl.) [%]	99.0	
(c) Single strongly coupled spin systems		
*R* ^2^	0.999	0.978
MAE [Hz]	0.76	0.57
MedAE [Hz]	0.53	0.20
Acc. [%]	99.7	
Acc. (excl.) [%]	99.3	
(d) Experimental test set		
*R* ^2^	0.999	0.936
MAE [Hz]	1.37	0.47
MedAE [Hz]	0.89	0.20
Acc. [%]	93.5	
Acc. (excl.) [%]	92.1	

a Abbreviations: *R* ^2^, coefficient of determination; MAE, mean absolute error; MedAE, median absolute error; Acc., classification accuracy for proton counts. Acc. (excl. “no spin”) reports the accuracy excluding the “no spin” class. Column headers: δ (chemical shift), *J* (coupling constants).

The classification performance of MolDeTr demonstrated exceptional results, achieving an overall accuracy of 99.1%, inclusive of the “no spin” class, and 97.5% when this class was excluded. This high level of accuracy is attributed to MolDeTr’s ability to perform relational reasoning, enabling it to accurately interpret signal-splitting patterns and deduce proton counts, even in normalized spectra without absolute scaling. This contrasts with traditional signal integration methods,^65^ which often depend on integral standard references to quantify proton counts accurately, overlooking coupling and splitting. In particular, MolDeTr maintains strong performance even in complex scenarios involving multiple overlapping spin systems. The model’s consistent precision in the face of spectral distortions highlights its robustness and reliability for quantitative predictions. In particular, the measurable decay of the free induction decay (FID) *T*_2_* is subject to extrinsic distortions, most notably phase errors and field inhomogeneities, that broaden and reshape spectral lines. Our approach effectively attempts to disentangle these external perturbations, enabling an accurate retrieval of the intrinsic transverse relaxation time *T*_2_ used in the spin system simulations and yielding a mean absolute error of 20 ms. Additional results for transverse relaxation times *T*_2_ and signal regions, as well as more details on the presented results in this Section, are found in the Supporting Information Section 4.1.

### Breaking New Ground in Spectral Interpretation: MolDeTr Excels in Analyzing Strongly Coupled Systems

To further assess MolDeTr’s capabilities in the spectral analysis of small molecules using 1D proton NMR, we categorized spin systems into weakly and strongly coupled groups based on the ratio |Δδ·ν_o_ / J|, where Δδ represents the chemical shift difference (ppm) , ν_o_ the base frequency (Hz), and *J* the coupling constant (Hz), using a threshold of 10. In this specific data set of 10,000 spectra consisting of single weakly or strongly coupled spin systems only, designed to resemble interacting hydrogen (proton) groups in molecules, MolDeTr achieved high classification accuracies of 99.0% and higher for both weakly and strongly coupled systems. The model consistently predicted key spectral features, including chemical shifts and coupling constants (Table 1b,c). Although weakly coupled systems benefited from more apparent signal shapes and reduced overlap, MolDeTr also excelled in strongly coupled systems, which pose more significant challenges and are often deemed impractical for purely manual interpretation.

Our results demonstrate that MolDeTr maintains robust performance across both weakly and strongly coupled spin systems, even as spectral complexity increases with multiple overlapping spin systems (see Table 1a). This reveals that MolDeTr can extract valuable insights from difficult spectral data, where traditional expert analysis and algorithms may struggle. The Supporting Information Sections 4.2 and 4.3 provide additional results for both coupling regimes.

### Bringing Quantitative Analysis to Low Magnetic Fields: Exemplified for Ethyl Groups

In this section, we showcase MolDeTr’s capability to accurately analyze and reconstruct the spectrum of an ethyl group at varying low magnetic field strengths (Figure 3). The ethyl group is a well-characterized spin system comprising a methylene group (H_A_2__) and a methyl group (H_B_3__). For illustrative purposes, the ground truth parameters in the simulated, distorted spectra remain consistent across all fields. The chemical shifts are 3.70 ppm for the methylene protons and 1.20 ppm for the methyl protons. The coupling constant between these groups is 7.00 Hz, and the transverse relaxation times *T*_2_ are 531 and 455 ms, respectively.

The predicted parameters obtained from MolDeTr for each spectrum are as follows:

- Spectrum at 80 MHz: predicted shifts at 1.20 and 3.71 ppm, coupling constant of 6.98 Hz, and transverse relaxation times *T*_2_ of 531 and 460 ms.
- Spectrum at 20 MHz: predicted shifts at 1.21 and 3.71 ppm, coupling constant of 6.99 Hz, and transverse relaxation times *T*_2_ of 505 and 455 ms.
- Spectrum at 10 MHz: predicted shifts at 1.23 and 3.73 ppm, coupling constant of 7.04 Hz, and transverse relaxation times *T*_2_ of 505 and 448 ms.
- Spectrum at 5 MHz: predicted shifts at 1.17 and 3.57 ppm, coupling constant of 6.97 Hz, and transverse relaxation times *T*_2_ of 578 and 505 ms.

As the base frequency (ν_0_) decreases, the spectra transition from weak to strong coupling regimes, and the SNR decreases due to lower sensitivity at reduced field strengths.^66^ These conditions traditionally present significant challenges for spectral interpretation, complicating quantitative analysis and accurate reconstruction with existing methodologies. We first used MolDeTr to obtain parameter predictions for each spectrum. Then, we utilized these predicted parameters to construct the spin system and simulate the spin system, which generated the reconstructed spectra. Despite the inherent analytical difficulties at lower field strengths, MolDeTr successfully recovers the spectral parameters even at 5 MHz, demonstrating excellent agreement between ground truth and reconstructed spectra. This underscores MolDeTr’s robustness and precision under diverse experimental conditions and extends advanced quantitative and automated analysis to low-field NMR spectroscopy. Note that reconstructions could be further improved with fitting algorithms such as DAISY^67^ or the method of De Lorenzi et al.^63^ that could exploit MolDeTr predictions as priors.

### Enabling Unprecedented Analysis across Diverse and Complex Experimental Spectra

To demonstrate MolDeTr’s versatility and reliability in practical applications, we applied it to a diverse set of experimental 1D proton NMR spectra of small molecules, with proton base frequencies ranging from 80 to 600 MHz. Gaussian noise at 0.1% of the maximum signal intensity was added to minimize spurious correlations occasionally introduced by preprocessing steps such as line broadening. To assess MolDeTr’s robustness under varying noise conditions, the analysis was repeated five times with different random noise seeds, allowing us to evaluate the prediction variability.

Our data set included 12 expert-annotated experimental spectra, corresponding to 13 analyzed regions of interest (ROIs), featuring regions with overlapping spin systems and strong coupling effects that typically require prior knowledge of the spin systems and quantum mechanical simulations for precise interpretation. In particular, 7 of the 12 spectra were measured on 80 MHz benchtop spectrometers. Refer to Supporting Information Section 4.4 for more details on the experimental test set. We note that this experimental set is modest in size and that the molecules are small. From an NMR standpoint, however, the analytical difficulty lies in dissecting the spectrum into its underlying NMR parameters (the spectral inverse problem) rather than in the size of the molecule, and is concentrated in the strongly coupled, overlapping aromatic regions of the set (for example the vanillin and cinnamic acid spin systems); the overlap and coupling regime of every region of interest are detailed in Table S7 and Supporting Information Section 4.4. Several of the test compounds feature heteroatoms bonded directly to or adjacent to the analyzed aromatic ring systems: ethyl vanillin and vanillin carry oxygen substituents (methoxy, hydroxyl, aldehyde) on the ring, caffeic acid has two hydroxyl groups at ring positions 3 and 4, and the benzoate aromatic protons in cocaine are adjacent to an ester oxygen. Further compounds such as ibuprofen and cinnamic acid contain carboxylic acid groups near the aromatic system. For the complete list of experimental spectra, their source publications, compounds, solvents, magnetic field strengths, and evaluated spectral windows, we provide Table S7 and Figures S17–S21 in Supporting Information Section 4.4.

MolDeTr accurately predicted chemical shifts and coupling constants in these complex experimental spectra. The predicted chemical shifts closely matched the values measured experimentally, with residuals predominantly within ± 3.5 Hz (Figure 4a,b). The coupling constants were predicted with high precision, with residuals mainly within ± 1 Hz and a slight overestimation bias observed (Figure 4c,d). An outlier in coupling constant predictions highlights that higher residuals often align with increased variance, aiding in identifying and validating uncertain predictions. Despite challenging spectral features and experimental distortions, the mean absolute errors were 1.368 ± 1.075 Hz for chemical shifts and 0.470 ± 0.543 Hz for coupling constants. The median absolute errors were 0.885 and 0.199 Hz, respectively (Table 1d). These metrics underscore MolDeTr’s substantial accuracy and precision in quantitative predictions, even in complex experimental spectra.

**Figure 4:**
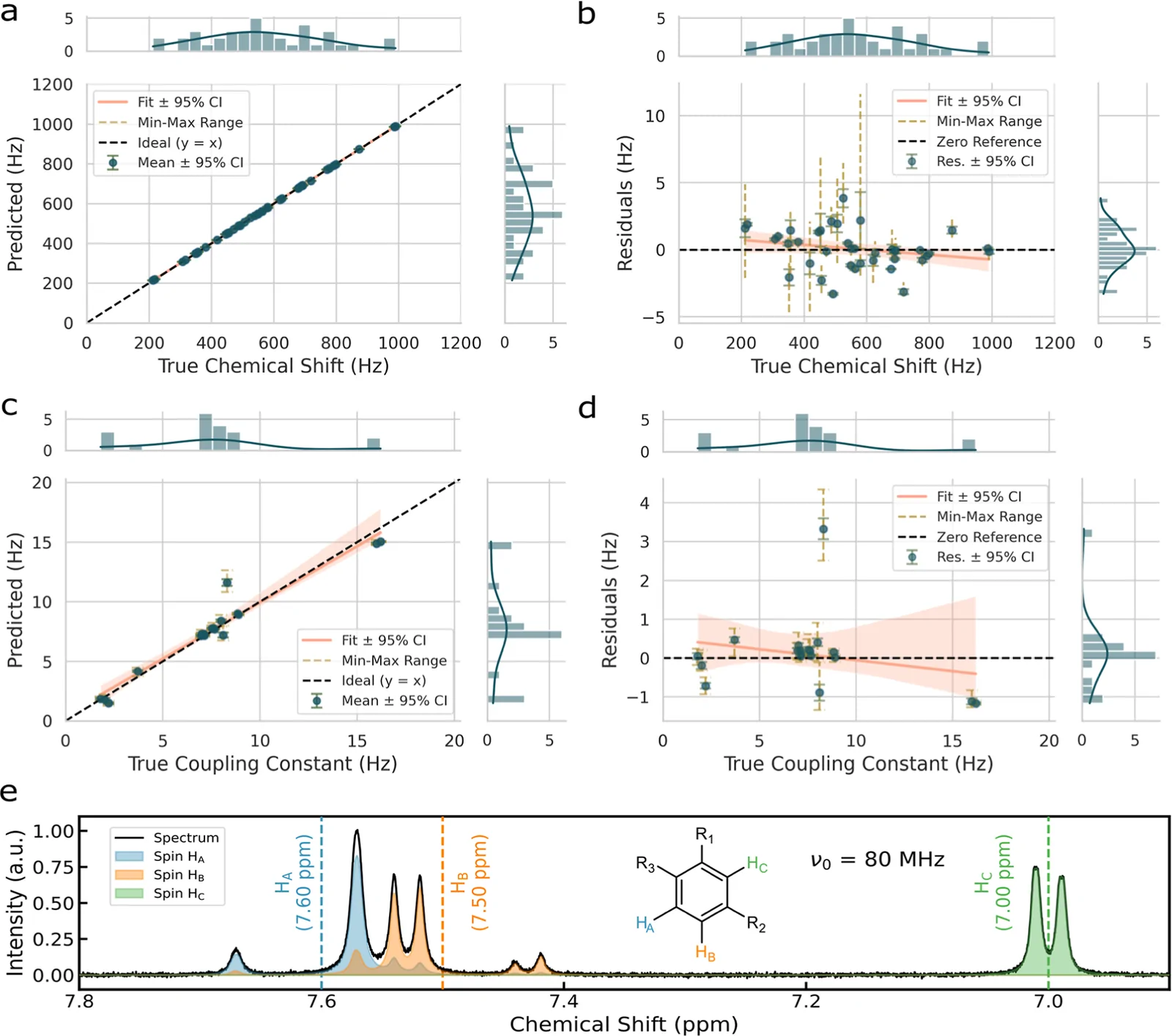
Performance evaluation of MolDeTr predictions for chemical shifts and coupling constants. (a,c) Predicted versus true values for chemical shifts and coupling constants, respectively. Data points represent the mean prediction across five runs, with error bars denoting the 95% confidence intervals and the extrema (min and max) across these runs indicated for each true parameter. The dashed black line indicates perfect agreement (*y* = *x*), while the red line represents the fitted regression with a 95% confidence interval shaded in pink. (b,d) Residual plots for chemical shifts and coupling constants, illustrating prediction errors with distributions shown in the side histograms. For reference, the horizontal dashed line represents zero error. (e) Aromatic region of a simulated 1,2,4-trisubstituted benzene derivative (ABC spin system) at 80 MHz. The full spectrum (black line) is overlaid with contributions from individual spins (H_A_, H_B_, and H_C_), indicated by vertical dashed lines at their corresponding chemical shifts.

In addition to regression tasks, we evaluated MolDeTr’s performance in classifying proton counts. The overall accuracy of the proton count prediction was 93.5% and 92.1% when excluding “no spin”. These results highlight MolDeTr’s effectiveness in handling various proton counts in experimental data; refer to Supporting Information Section 4.5 for more details.

### Experimental Case Studies Demonstrating MolDeTr’s Analytical Capabilities

To illustrate MolDeTr’s capabilities on experimentally acquired NMR data, we examine the aromatic region of vanillin as a representative example (Figure 1b and Table 2a). This ABC spin system exhibits partially overlapping resonances that challenge conventional spectral analysis methods. MolDeTr accurately predicts chemical shifts (7.39, 7.42, and 6.96 ppm for spins A, B, and C, respectively), coupling constants, and transverse relaxation times *T*_2_, closely reflecting the ground truth values. This example illustrates the structure-elucidation use case outlined in the Introduction: given only the raw ^1^H spectrum, without prior knowledge of the spin system or molecular structure, MolDeTr extracts chemical shifts, coupling constants, and proton counts that directly constrain the substitution pattern and local connectivity of the aromatic fragment. The slight overestimation bias in relaxation time may arise because MolDeTr attempts to extract only the portion of line width related to transverse relaxation time *T*_2_, excluding contributions from field inhomogeneities and phase errors, which are typically included in expert annotations. For an additional experimental example involving cinnamic acid and its complex aromatic AA′BB′C spin system, please refer to Supporting Information Section 4.6.

**Table 2:** (a) Comparison of Ground Truth and MolDeTr Predictions for the Experimental Vanillin (ABC Spin System)^a^

spin	H count	δ (ppm)	*J* (Hz)	*T*_2_ (ms)
(a) Vanillin Aromatic Region (ABC Spin System)				
Ground Truth				
A	1	7.39	2.0	455
B	1	7.42	8.1, 2.0	637
C	1	6.96	8.1	531
Mnova (no prior)				
A	2	7.41	—	—
B	—	—	—	—
C	1	6.96	8.0	424
MolDeTr (no prior)				
A	1	7.39	2.0	557
B	1	7.42	8.7, 1.5	637
C	1	6.96	8.1	707
(b) 1,2,4-Trisubstituted Benzene Derivative (ABC Spin System)				
Ground Truth				
A	1	7.60	8.0	455
B	1	7.50	2.0, 8.0	531
C	1	7.00	2.0	637
Mnova (no prior)				
A	2	7.53	—	—
B	—	—	—	—
C	1	7.00	1.7	435
MolDeTr (no prior)				
A	1	7.59	8.1	448
B	1	7.50	1.3, 8.1	577
C	1	7.00	1.8	637

a (b) Comparison of ground truth, Mnova, and MolDeTr predictions for a distorted simulated 1,2,4-trisubstituted benzene derivative (ABC spin system). Column headers: spin label; H count = proton count; δ = chemical shift; *J* = coupling constants; *T*_2_ = transverse relaxation time.

### MolDeTr Outperforms Leading Analytical Software in the Interpretation of Difficult Spectra

We compared both methods using two representative cases to benchmark MolDeTr against a widely used, state-of-the-art NMR analysis platform (Mnova).^19^ First, we examined the experimentally acquired vanillin spectrum (Table 2a), where Mnova, also operating without prior knowledge of the spin system, could not resolve overlapping resonances and predict their coupling constants. Second, we evaluated a distorted, simulated _ABC_ spin system (Figure 4e and Table 2b), for which the ground truth parameters were known precisely. In this scenario, Mnova struggled to disentangle the strongly coupled spins, thereby limiting its ability to extract accurate parameters. While the program performs well with straightforward spectra, its underlying algorithm is not designed to handle the complexity introduced by strongly coupled and overlapping resonances. In contrast, MolDeTr successfully distinguished all spins and accurately predicted the parameters in both spectra.

## Discussion

MolDeTr advances NMR spectral analysis by employing a sequence-to-set detection transformer-based architecture that adaptively focuses on intricate relationships between molecular properties and their spectra. Unlike traditional heuristic pipelines—consisting of peak picking, multiplet aggregation, multiplet integration, and coupling constant extraction—or sequence-to-sequence paradigms common in large language models for chemistry,^68^ MolDeTr directly maps spectral data to spins and atoms represented as nodes in a molecular graph. This chemistry-informed model design, combined with tailored loss functions and attention mechanisms, captures both local spectral and molecular features and broader global relationships, enabling insights that would usually require different 2D NMR experiments. As a result, MolDeTr offers more consistent, problem-specific solutions and enables a precise, quantitative interpretation of complex molecular interactions. It is a next-generation analytical tool that reflects the expertise of skilled spectroscopists and sometimes even surpasses it.

In that inverse setting, the spectrum-conditioned experimental benchmarks (Table 1d) and case-study comparisons (Table 2) reported in this manuscript quantify MolDeTr’s parameter extraction performance without relying on structural priors. Notably, MolDeTr attains Hz-level accuracy for chemical shifts and sub-Hz accuracy for coupling constants, corresponding to improvements of approximately one to 2 orders of magnitude over typical structure-based predictors for chemical shifts, e.g. DFT or HOSE codes, with the largest relative gains observed at higher magnetic fields.

MolDeTr harnesses synthetic data generated through high-fidelity quantum mechanical simulations of spin systems, enhanced with realistic spectral distortions to replicate experimental conditions. This strategic use of simulated data is crucial for successful model training and addressing the significant practical challenges associated with the time-consuming and costly acquisition and annotation of experimental NMR data. By establishing a robust framework that produces accurately labeled molecular spin systems and spectra closely mirroring real-world experimental data, MolDeTr sets a new standard for synthetic NMR data generation.

As a result, it delivers exceptional performance across a broad range of simulated and experimental 1D proton NMR spectra. MolDeTr outperforms established software such as Mnova in resolving complex spin systems and adeptly manages diverse spin systems under various experimental conditions.

The impact of MolDeTr extends to enhancing spectral analysis under challenging conditions, such as low signal-to-noise ratios, strong couplings, and crowded spectral regions typical of low-field and benchtop 1D NMR experiments. These problematic conditions would often necessitate more time-consuming and sample-intensive 2D NMR experiments. Hence, MolDeTr’s capabilities are particularly advantageous for applications that require rapid and accurate analysis, including drug discovery, metabolomics, and environmental monitoring. Moreover, the accessibility and affordability of low-field and benchtop spectrometers, combined with MolDeTr’s quantitative analytical capabilities at lower fields without requiring expert knowledge, extend access to advanced spectral analysis. This advancement makes such analysis feasible for smaller facilities and laboratories with limited resources and extends its applicability across a broader range of research environments. Notwithstanding these strengths, several current scope limitations warrant note.

MolDeTr currently targets prototypical spin systems of single-component small molecules from 1D ^1^H NMR spectra. Spectra with extreme congestion reflect the ill-posed nature of the inverse problem and cannot in general be uniquely resolved, a limitation shared by manual and automated analyses. Mixtures, impurities, and solvent peaks are out of scope. Reported performance assumes standardized 1200 Hz windows and pertains to the distortion ranges used for training (Supporting Information Section 3.2.2). The model capacity comprises ten spin queries per window. This is an engineering choice, not a fundamental constraint, and can be scaled. Contextual and relational cues are integrated as in-spectrum features through self-attention in the encoder and decoder, cross-attention and positional embeddings. Acquisition metadata such as solvent, temperature, or pH are not modeled explicitly, but implicitly addressed through systematic variations of spin system parameters and distortions. Because MolDeTr solves the spectrum-conditioned inverse problem, it extracts spin-system parameters as they appear in the observed spectrum regardless of what environmental factors caused the observed chemical shifts. The experimental test set (Table S7 in the Supporting Information) confirms this empirically: spectra were acquired in five different solvents (CDCl_3_, DMSO-*d*_6_, C_6_D_6_, D_2_O, and acetone-*d*_6_), and the model generalized across all conditions. It should be noted that the experimental test set, while diverse in coupling regimes, field strengths, solvents, and heteroatom environments, remains modest in size. A fundamental challenge in assembling larger annotated benchmarks is that raw NMR spectral data (free induction decays and fully processed spectra) are rarely deposited alongside publications; typically only extracted parameters such as chemical shifts and coupling constants are reported.^69^ Constructing ground-truth spin-system annotations therefore requires access to original raw data combined with labor-intensive expert interpretation and quantum-mechanical validation.

MolDeTr elucidates and quantifies molecular structures and dynamics without the need for prior compound-specific spin system information, expert knowledge, and reference standards, allowing applications in characterizing unknown drug degradation products in pharmacokinetics, identifying bioactive compounds in natural product research, and facilitating biomarker discovery in clinical diagnostics.^2,3^ By enabling rapid and accurate spectral data analysis, MolDeTr reduces the time and resources needed for research and development, fostering innovation and enhancing scientific discovery efficiency.

MolDeTr propels automated spectral analysis forward by leveraging its chemistry-informed detection transformer architecture alongside highly realistic simulated data. This synergy enhances the consistency and accuracy of spectral interpretations and broadens the conditions under which molecular properties and their corresponding spectra can be quantitatively analyzed in an automated fashion. As a result, MolDeTr strengthens analytical capabilities and facilitates various applications by providing swift and reliable spectral analyses. This advancement accelerates innovation and scientific discovery across various disciplines, making sophisticated spectral analysis more accessible and efficient for researchers, especially in resource-limited and high-throughput environments. As demonstrated with the vanillin case study (Figure 1b and Table 2), MolDeTr provides a practical tool for structure elucidation of unknown single-compound analytes, converting overlap-limited ^1^H spectra into quantitative spin-system constraints across multiple application domains.

## Supplementary Material



## Data Availability

The source code for MolDeTr is openly available on GitHub (https://github.com/smidooo/MolDeTr) under the Apache License 2.0, and a citable snapshot of each release is archived in Zenodo under the concept DOI https://doi.org/10.5281/zenodo.21214876, which always resolves to the most recent version. The data supporting this study are openly available in Zenodo at https://doi.org/10.5281/zenodo.21217102. This deposit contains the trained model weights, the 13 experimental regions of interest (ROIs) used for evaluation (80–600 MHz), and a representative subset of the synthetic spectra, together with their ground-truth spin-system annotations. Full details of the data curation are provided in the Supporting Information (Section 4.4).

## Notes

Additional methods and implementation details; extended benchmarking tables and metrics; training data generation parameters; experimental spectra descriptions; supplementary figures S1–S23 and supplementary tables S1–S8; and source data for figures. (PDF)
