# Supplementary material for: MolDeTr: A Chemistry-Informed Deep Learning Model for Next-Generation Automated Analysis of 1H NMR Spectra
Source: Anal Chem. 2026 Aug 4;98(32):23399–412. doi: 10.1021/acs.analchem.5c03465 (PMC13492045; doi:10.1021/acs.analchem.5c03465)
Supplement: Supplementary file 1 [file ac5c03465_si_001.pdf]

Supplementary Information

MolDeTr: A Chemistry-Informed Deep Learning Model for Next-Generation Automated Analysis of ^{1}H NMR Spectra

Nicolas Schmid[1,2,3,*] Marc Wanner[4] Giulia Fischetti[1,5] Andreas Henrici[1]

Mohsen Meshkian[1] Simon Bruderer[6] Rudolf M. Füchslin[1,7] Bjoern Heitmann[6]

Jan Dirk Wegner[2] Roland K. O. Sigel[3] Dirk Wilhelm[1,*]

[1]Institute of Applied Mathematics and Physics (IAMP), Zurich University of Applied Sciences (ZHAW), Technikumstrasse 71, 8401 Winterthur, Switzerland

[2]Department of Mathematical Modeling and Machine Learning (DM3L), University of Zurich (UZH), Winterthurerstrasse 190, 8057 Zurich, Switzerland

[3]Department of Chemistry, University of Zurich (UZH), Winterthurerstrasse 190, 8057 Zurich, Switzerland

[4]Computer Vision Laboratory (CVL), ETH Zurich, Sternwartstrasse 7, 8092 Zurich, Switzerland

[5]Ca' Foscari University of Venice, Dorsoduro 3246, 30123 Venice, Italy

[6]Bruker Switzerland AG, Industriestrasse 26, 8117 Fällanden, Switzerland

[7]European Centre for Living Technology (ECLT), Ca' Bottacin, Dorsoduro 3911, Calle Crosera, 30123 Venice, Italy

*Corresponding authors: `nicolas.schmid.research@gmail.com`, ORCID: 0000-0003-1930-7654; `wilk@zhaw.ch`, ORCID: 0000-0001-5109-9803

1 Automated Spectral Data Analysis: Challenges and Opportunities in NMR and Beyond

The automated analysis of spectral data, particularly in NMR, is pivotal for advancing chemical research and discovery. However, this automation faces numerous challenges that hinder its efficiency and accuracy. This section delves into the specific obstacles encountered in 1D proton NMR spectra, explores innovative global strategies that offer potential solutions, and examines the role of neural networks in enhancing the interpretability of automated analyses.

1.1 Specific Challenges in 1D Proton NMR Spectroscopy

Automating the analysis of spectral data, particularly in NMR, presents significant challenges. These challenges include managing complex spin systems, addressing strong coupling (non-linear interactions), dealing with low SNR, addressing signal distortions, and resolving overlapping resonances. While these issues are prominent in proton NMR, many are also manifested in other spectral techniques such as carbon-13 NMR, 2D NMR, IR, and MS. Table S1 summarizes these challenges and their impact on both manual and automated analysis.

Table S1: Key challenges in automated analysis of spectroscopy, focusing on 1D proton NMR.

**Challenge**	**Impact on Experts**[1]	**Impact on Automated Analysis**
**Complex Spin Systems**	Manually resolving interactions in systems with multiple coupling pathways or chemically but not magnetically equivalent nuclei is time-consuming and requires deep expertise [2].	Algorithms often struggle to predict the behavior of complex spin systems without prior spin system knowledge, leading to incomplete or inaccurate results [3, 4].
**Strong Coupling**	Higher-order splitting patterns complicate interpretation, often requiring simulations to accurately resolve [4, 2].	Non-linear effects confuse algorithms, leading to errors in predicting J-coupling constants and other key parameters [2, 4].
**Low SNR**	Weak signals make it challenging to distinguish real peaks from noise, particularly in low-concentration samples, low magnetic fields, or complex mixtures [5].	Algorithms may miss critical peaks or apply excessive noise reduction, potentially erasing important spectral features [6].
**Signal Distortions**	External factors, such as instrumental limitations or sample impurities, often cause distortions, requiring manual correction [6].	Distortions introduce artifacts that lead to inaccurate peak identification or parameter prediction unless carefully addressed [7, 5].
**Overlapping Resonances**	In crowded spectra, overlapping peaks often require expert deconvolution and prior knowledge of the spin system [2, 4].	Automated systems can misinterpret overlapping signals, leading to mispredictions, particularly in densely packed spectral regions [8, 7].

In 1D proton NMR, these challenges are particularly pronounced due to the complexity of small molecules, which often exhibit intricate spin systems [1]. Proton NMR is further complicated by the relatively narrow chemical shift

range of protons, increasing the likelihood of spectral overlap and ambiguity. This overlap can obscure essential features, making it difficult for both human analysts and automated systems to extract accurate molecular information.

Experts typically rely on a combination of experience, prior knowledge, and advanced simulation techniques to interpret difficult spectra. This process is time-intensive and depends on human judgment. In contrast, algorithms should be designed to handle these challenges without relying heavily on prior knowledge, a significant limitation in current automated systems [5].

1.2 Contextual and Relational Cues in 1D Proton NMR: Foundations for Expert and Algorithmic Analysis

1D proton NMR spectroscopy is a fundamental technique for determining the structures of organic molecules. It offers detailed information about the molecular environment, connectivity, and dynamics of hydrogen atoms (proton spins) within a compound. Accurate interpretation of proton NMR spectra, whether performed by experienced chemists or advanced algorithms, relies on effectively identifying and integrating contextual and relational cues. These cues are essential for retrieving molecular structures and dynamics with high precision and reliability.

1.2.1 Relational Cues

Relational cues involve the interactions and connections between signals in the spectrum and protons in the molecule, elucidating how these atoms are linked and influence each other. Understanding these interactions is crucial for mapping the molecular framework and deciphering the spin systems present. Key relational cues in proton NMR include:

- **Spin-Spin Coupling Constants (J-Coupling)**: J-coupling results from interactions between neighboring protons, causing the signal to split into multiplets such as doublets, triplets, and quartets. The number of splits and their intensities reveal the number of adjacent protons and the strength of their interactions, aiding in determining the spatial arrangement of protons within the molecule. Higher-order and long-range couplings can provide further structural insights, albeit with increased spectral complexity.
- **Multiplet Structures**: Following the $n + 1$ rule, where n is the number of neighboring protons, multiplet patterns help identify distinct proton environments and delineate spin systems. Complex phenomena like rooftopping (roof-shaped pattern), where strong coupling distorts expected splitting patterns, offer additional information about the proximity and direction (up-field/down-field) of the coupling protons in the spectrum. Advanced models, such as our transformer-based MolDeTr, effectively decode these intricate patterns to extract meaningful structural data.
- **Relative Signal Integrals**: The integral of each NMR signal corresponds to the number of protons contributing to that signal. Accurate integration is vital for assigning peaks to specific proton environments and verifying the stoichiometry of different proton types within the molecule, ensuring consistency with the proposed molecular structure.
- **Line Widths (T_2 Relaxation Times)**: The width of NMR signals provides insight into the molecular dynamics and local environment of protons. Broader lines may indicate rapid relaxation processes, interactions with other molecular entities, or dynamic processes such as chemical exchange. Variations in line widths help differentiate between different proton types and elucidate their mobility and interactions within the molecule.
- **Coupling with Heteroatoms**: Interactions between protons and heteroatoms (e.g., ^{1}H-^{15}N or ^{1}H-^{13}C couplings) offer additional structural information. Although direct observation of these couplings in 1D

proton NMR is limited due to the low natural abundance of ^{13}C and ^{15}N, their indirect effects can influence the spectra. For instance, heteronuclear couplings can cause subtle shifts or splittings in proton signals, providing insights into the electronic structure and molecular framework beyond proton-proton interactions.

- **Spectral Distortions as Relational Cues**: Certain spectral distortions can offer relational information by systematically influencing multiple signals:
  - **Shim and Line Width Distortions**: Field inhomogeneities can cause line-shape distortions often affecting all signals similarly, indicating instrumental influences rather than molecular interactions.
  - **Phase Distortions**: Gradual phase shifts across the spectrum typically stem from experimental artifacts, allowing differentiation between genuine molecular signals and systematic distortions.
  - **^{13}C Satellites**: These appear symmetrically around proton signals, providing information about the signal of the proton they are interacting with.
  - **Solvent and Impurity Peaks**: Recognizing and excluding solvent residues or impurities prevents misinterpretation of these signals as part of the target molecule's spin system.

Together, these relational features enable the reconstruction of the molecular framework by illustrating proton interactions and connectivity, facilitating accurate determination of chemical shifts, coupling constants, and proton counts essential for comprehensive structural elucidation.

1.2.2 Contextual Cues

Contextual cues encompass the broader chemical and experimental conditions that inform the interpretation of proton NMR spectra. These cues are critical for ensuring accurate signal assignments and minimizing misinterpretations. Key contextual cues include:

- **Characteristic Chemical Shift Regions**: Protons in specific functional groups resonate within defined chemical shift ranges (e.g., aldehyde protons around 9–10 ppm, aromatic protons between 6–8 ppm). Identifying these regions aids in assigning signals to functional groups based on the expected chemical environment and serves as a prior for prototypical spin systems in these regions.
- **Spectral Crowdedness**: High signal density can lead to overlapping peaks, necessitating careful deconvolution to ensure accurate peak assignments, especially in molecules with multiple similar proton environments.
- **Solvent and Reference Peaks**: Correct identification and exclusion of solvent and reference compound signals prevent their misassignment as part of the target molecule's structure.
- **Environmental Effects**: Factors such as temperature, solvent polarity, and concentration can influence chemical shifts and line widths. Understanding these effects provides context for spectral variations and aids in accurate signal interpretation.

- **Dynamic Processes**: Chemical exchange, conformational changes, and molecular tumbling rates can impact chemical shifts and line widths. Recognizing these dynamic processes is essential for accurate signal assignment and understanding molecular behavior.
- **Instrumental Parameters**: Magnetic field strength, pulse sequences, and receiver settings affect spectral quality and resolution. Accounting for these parameters ensures proper interpretation and facilitates meaningful comparisons across different NMR datasets.
- **Spectral Distortions**: Various factors can introduce distortions, including:
  - **Baseline Distortions**: Imperfections in the baseline can obscure or mimic real signals, complicating peak integration.
  - **Phase Distortions**: Incorrect phasing can lead to distorted peak shapes, affecting accurate interpretation.
  - **Noise and Impurities**: Random electronic noise or sample contaminants can obscure weak signals or create false positives.
- **Spectral Distortions as Contextual Cues**: Recognizing shared distortions across multiple peaks can indicate common underlying factors, such as instrumental artifacts or consistent experimental conditions. This awareness allows for adjustments in analysis to account for their impact, enhancing the accuracy of structural interpretations.

These contextual cues complement relational cues, providing a comprehensive framework that enhances the accuracy and reliability of proton NMR analysis.

1.2.3 Practical Application: Ethanol Example

To illustrate the integration of contextual and relational cues in proton NMR analysis, consider the spectrum of ethanol (Figure S1b):

- **Spin-Spin Coupling**: The methyl protons (H_a) and methylene protons (H_b) exhibit distinct splitting patterns, a triplet and a quartet, respectively. These patterns indicate the connectivity between H_a and H_b, confirming the presence of an ethyl group.
- **Multiplet Structures and Proton Counts**: The triplet for H_a corresponds to three protons, while the quartet for H_b corresponds to two protons. The relative integrals of these multiplets align with the expected proton counts, validating their assignment to specific environments.
- **Rooftopping Effects**: Strong coupling between H_a and H_b can cause rooftopping, where multiplet splitting patterns are distorted and tilted towards each other. This warping provides insights into the proximity and chemical environments of the coupling partners. Our transformer-based model, MolDeTr, effectively decodes these complex patterns to extract structural information.

- **Chemical Shifts**: The chemical shifts of H_a and H_b fall within the aliphatic region, supporting their identification as methyl and methylene protons, respectively.
- **Line Width of Hydroxyl Proton** (H_c): The broad line width of the hydroxyl proton indicates rapid exchange with the solvent (e.g., dimethyl sulfoxide (DMSO)), reflecting the dynamic behavior of the hydroxyl group. In contrast, in a non-hydrogen-bonding solvent such as $CDCl_3$, the resonance is sharper and upfield, see Figure 2a, illustrating the influence of solvent interactions on chemical shifts.

This example demonstrates how the synergy between contextual and relational cues enables comprehensive and accurate interpretation of proton NMR spectra. By systematically analyzing coupling patterns, rooftopping effects, multiplet structures, signal integrals, line widths, and accounting for spectral distortions within appropriate chemical shift regions, both experts and algorithms like MolDeTr can deduce molecular spin systems with high confidence.

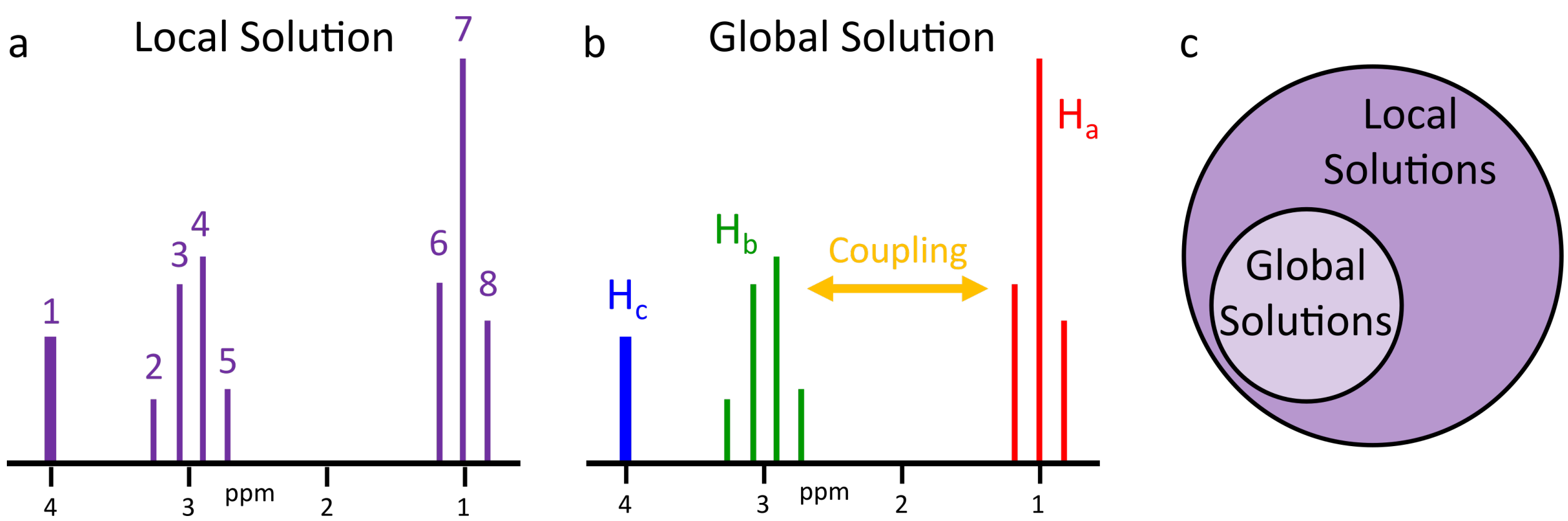

Figure S1: Analytical Approaches in Proton NMR Spectroscopy Illustrated with an Ethanol Spectrum. (a) *Local Approach*: Analyzes each peak independently based on individual parameters (chemical shift, intensity, and line width), potentially leading to multiple interpretations due to isolated consideration of each feature. (b) *Global Approach*: Treats the spectrum as an interconnected spin system, where peaks are parts of multiplet patterns governed by coupling interactions. The ethanol spectrum exemplifies both contextual and relational cues: distinct spin-spin coupling patterns of methyl (H_a) and methylene (H_b) protons appear as a triplet and a quartet, respectively. The broad hydroxyl proton (H_c) reflects rapid solvent exchange. Rooftopping effects in the multiplets provide insights into proton proximity and chemical environments. (c) The global perspective reduces parameter space and enhances coherence by recognizing inherent relationships within multiplets and coupling partners, facilitating more accurate spectral interpretations.

1.3 From Peaks to Patterns: Embracing Global Top-Down Strategies in NMR Analysis

In the analysis of NMR spectra, processes such as peak picking and deconvolution are often essential. These steps involve transforming the spectrum from a list of chemical shifts into a series of peaks and, subsequently, multiplets. We refer to this methodology as a *local* or *bottom-up* approach to spectrum analysis.

Consider ethanol's NMR spectrum, as illustrated in Figures S1a and S1b. The spectrum comprises eight distinct peaks, each characterized by three parameters: chemical shift, intensity, and line width. This local approach examines each peak independently, resulting in a detailed yet high-dimensional interpretation. Specifically, with eight peaks and three parameters per peak, the total parameter space spans 3×8 = 24 dimensions. While this strategy yields a detailed representation, it also introduces complexity, as each peak is treated in isolation, potentially leading to multiple interpretations due to the lack of relational context.

In contrast, the *global* or *top-down* approach (Figure S1b) parametrizes the spectrum by considering the molecule's spin system as a whole. Rather than focusing on individual peak characteristics, this perspective emphasizes broader, interconnected spectral features, such as multiplet patterns and coupling constants. In ethanol's case, the global approach recognizes the coupling between the methyl (H_a) and methylene (H_b) protons, which manifest as a triplet and quartet, respectively, due to their spin-spin coupling. The broad peak from the hydroxyl proton (H_c) reflects rapid solvent exchange. By treating these features as part of an interconnected system, the global approach condenses the parameter space significantly.

Instead of requiring separate parameters for each individual peak, the global approach groups related peaks into multiplets and simplifies the spectrum to key parameters. For ethanol, this means accounting for three chemical shifts, three proton counts, three line widths, and one coupling constant between H_a and H_b, resulting in a reduced parameter space of 10 dimensions. This holistic method assumes that the amplitude and pattern of peaks are determined by the proton count and the coupling interactions within each multiplet, rather than by isolated peak parameters.

As summarized in Figure S1c, the global approach not only reduces the dimensionality of the analysis but also enhances coherence by leveraging the inherent relationships between resonances. By considering the entire spin system, this strategy simplifies the analysis and mitigates the complexities inherent in NMR spectra, especially for molecules with more complex spin systems. The global approach is particularly advantageous when analyzing crowded regions or strongly coupled resonances, where a local or bottom-up strategy might become unwieldy and prone to error. It facilitates more accurate and coherent spectral interpretations by recognizing the collective behavior of protons and their interactions.

1.4 Decoding Neural Network Predictions: Visualizing Key Influences in NMR Spectrum Analysis

Understanding how neural networks make predictions is crucial for validating and improving their performance. Gradient norm visualizations are an effective tool for this purpose, allowing researchers to quantify the impact of each input feature on the model's output. Specifically, this technique calculates the L^2 norm of the gradient of the output with respect to the input features. In simpler terms, it measures how much a small change in the input data can influence the neural network's prediction [9].

When applied to NMR spectra, gradient norm visualizations help identify the precise points in the spectrum that most significantly affect the neural network's predictions of signal properties, such as the number of protons, coupling constants, or chemical shifts. High gradient norms at specific spectral points indicate regions where minor variations can lead to substantial changes in the predicted properties. This not only highlights the model's sensitivity to particular frequencies but also provides valuable insights into the neural network's decision-making process based on spectral data. Let $x \in \mathbb{R}^{6144}$ denote the input spectrum of a 1200 Hz window, and let k index a single predicted entity (equivalent spin group). We define a scalar output $y = (E_k(C))_{\max}$, i.e., the "max" component of the entity's 4-D J-embedding $E(C) = [\min, \max, \text{mean}, \sum]$. We then compute the saliency curve as $g = \left\|\partial y/\partial x\right\|_2$ by backpropagation with the trained network in evaluation mode. k is the prediction matched to a specific ground-truth entity via the Hungarian assignment. Saliency values are min–max normalized for display. Unless stated otherwise, we use $y = (E_k(C))_{\max}$; analogous maps can be produced for δ or Γ by substituting the scalar of interest.

Figure S2 demonstrates how the neural network focuses on specific frequencies within two distinct spin systems in an NMR spectrum. In the top panel, the network highlights the $\mathtt{A_1A_1'}$ spins within the $\mathtt{A_1A_1'X_1X_1'}$ system, particularly at 930 Hz. This emphasizes the relationship between the gradient norm and the coupling constant of this spin. Conversely, the bottom panel focuses on the $\mathtt{A_2}$ spin at 210 Hz within the $\mathtt{A_2X_2}$ system. For clarity, these key frequencies are enclosed in red boxes. Points with higher gradient norms are depicted with brighter colors, indicating the neural network's predictive focus. Frequencies with gradient norms below 0.45 are omitted to reduce visual clutter. Additionally, adjacent frequencies such as 750 Hz for $\mathtt{X_1X_1'}$ and 160 Hz for $\mathtt{X_2}$ are highlighted in blue boxes, showcasing increased gradient norms and indicating the model's attention to signals from coupling partners. This approach demonstrates how MolDeTr leverages interrelated spectral features across the spectrum to predict coupling constants of spins, facilitating the identification of coupling partners and the construction of spin systems based on the predicted parameters.

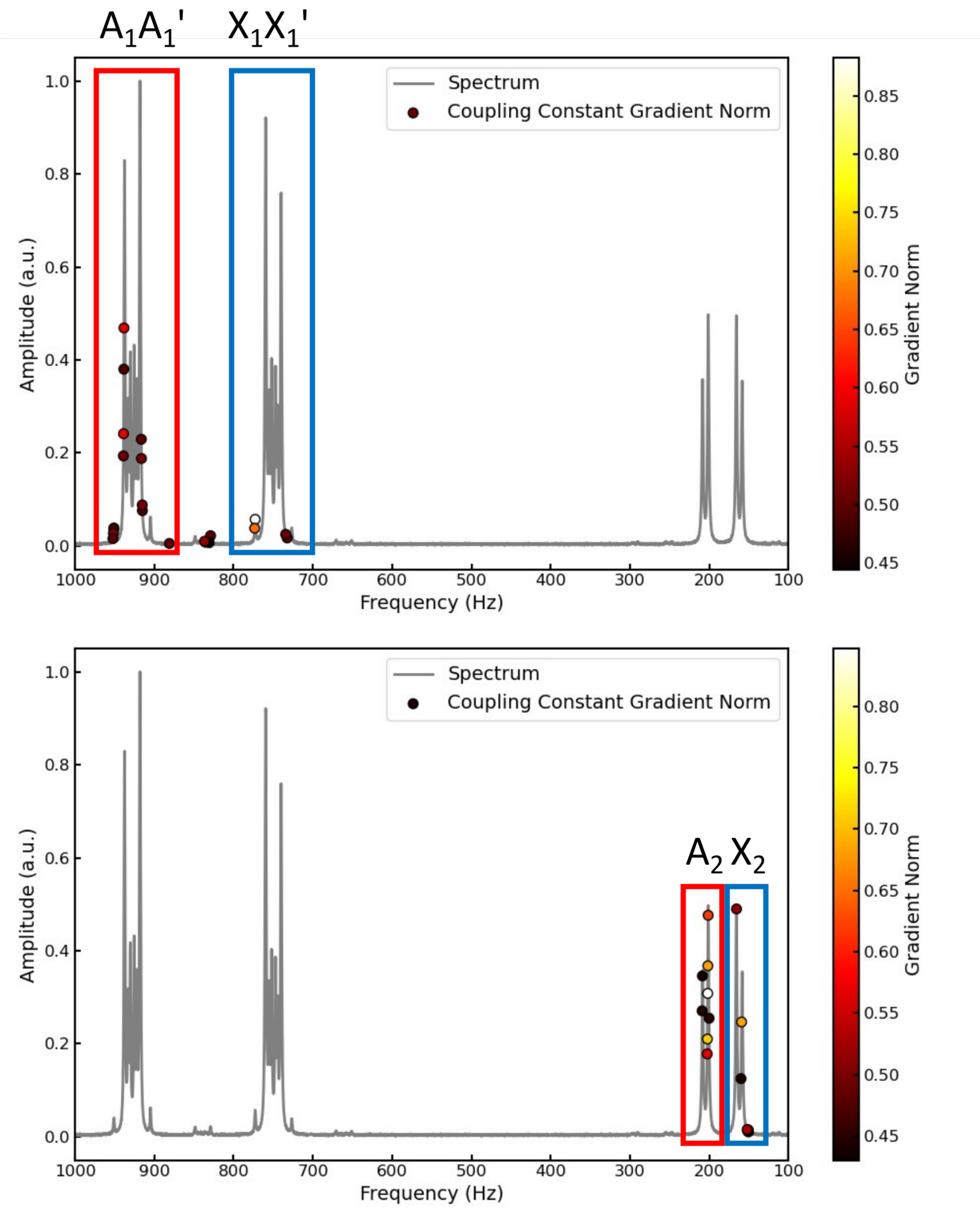

Figure S2: Gradient norm visualizations reveal the neural network's focus on specific frequencies within two spin systems in an NMR spectrum: the $A_1A_1'X_1X_1'$ system (top panel) and the A_2X_2 system (bottom panel). The top panel highlights the A_1A_1' spins at 930 Hz, while the bottom panel emphasizes the A_2 spin at 210 Hz, both enclosed in red boxes. Points with higher gradient norms are shown with brighter colors, indicating greater neural network emphasis. Blue boxes denote adjacent frequencies, such as 750 Hz for X_1X_1' and 160 Hz for X_2, which exhibit elevated gradient norms and reflect the model's attention to coupling partners.

1.5 Conceptual Analogy: Relational Attention in Language Processing and Spectral Analysis

To provide an accessible entry point for readers from diverse analytical chemistry backgrounds, we draw a conceptual parallel between the relational attention mechanisms used in language processing and in molecular spectroscopy. Figure S3 illustrates this shared principle.

In language processing, relationships extend beyond simple word matching to encompass grammar, syntax, and nuanced meanings that govern term co-occurrence and connections. For example, in English, the verb "eating" is closely associated with the noun "apple," reflecting a direct action-object relationship. In contrast, its connection to the adjective "shiny" is weaker, as the latter describes an attribute of "apple." Similarly, from English (input domain) to French (output domain), the verb "mange" aligns with "eating," demonstrating that transformer models can effectively map concepts across different linguistic structures despite variations in grammar and syntax.

A comparable principle applies to molecular spectroscopy, where patterns emerge from the physical and chemical laws governing atomic interactions rather than from linguistic structures. For instance, in an NMR spectrum (input domain) of ethanol (C_2H_6O), the protons of the CH_2 quartet are closely linked to those of the CH_3 triplet due to shared spin-spin coupling (J-coupling) interactions. At the same time, the OH singlet exhibits a weaker association with the triplet. These spectral relationships are governed by nuclear spin interactions, electronic environments, relaxation dynamics, and molecular geometry.

We emphasize that this analogy targets only the attention mechanism that is common to both domains: self-attention captures relational dependencies between entities (words or spin groups), and cross-attention aligns these entities with their encoded input representations. In both cases, queries, keys, and values are learned high-dimensional continuous vectors, and the scaled dot-product attention computation is identical. The domains differ fundamentally, however, in what the encoder receives as input: in language processing, the encoder operates on discrete deterministic tokens that appear identically in every instance and have unambiguous boundaries. In NMR spectroscopy, the encoder operates on a continuous noisy spectrum where signals are subject to overlap, strong coupling effects, and instrumental distortions. These upstream differences make the spectral inverse problem fundamentally ill-posed.

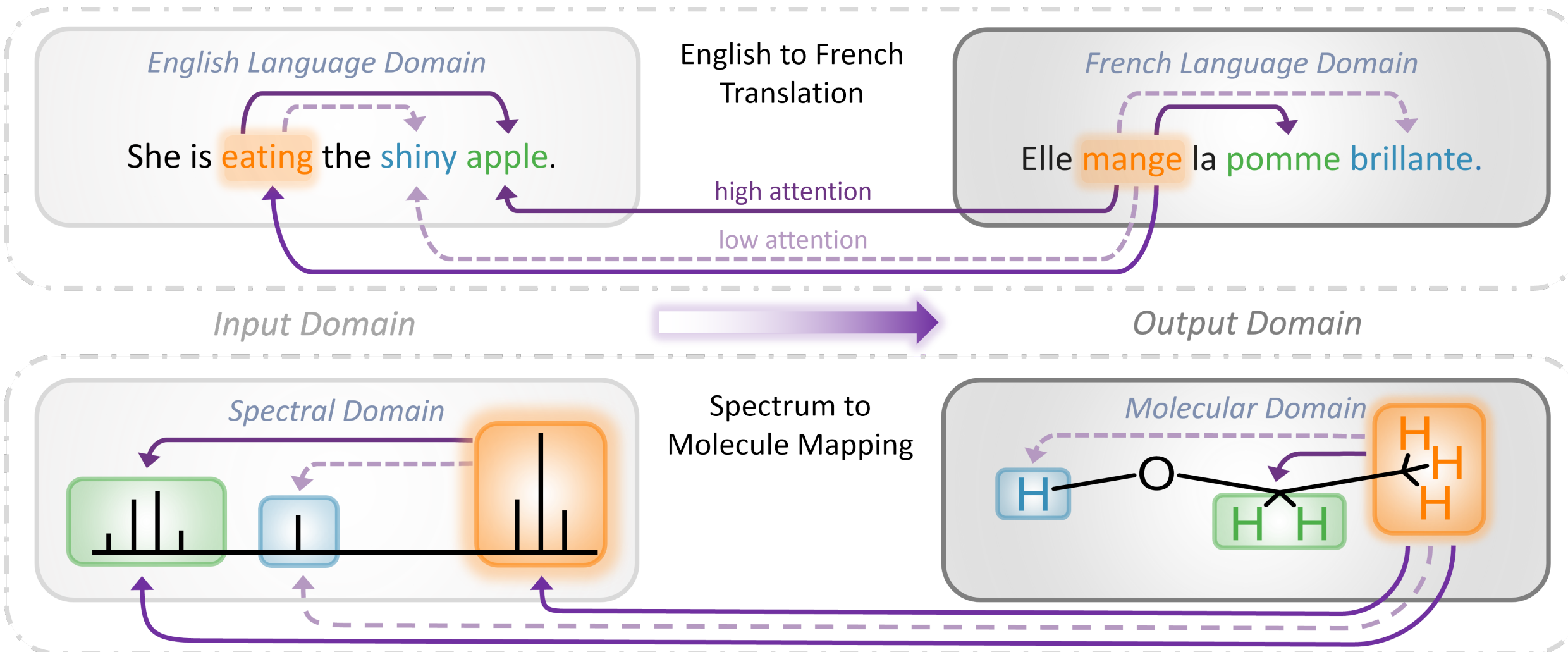

Figure S3: **Conceptual analogy between relational attention in language processing and spectral analysis.** In the language example (top), solid violet lines indicate strong connections and dashed lines weaker ones between English and French domains. In the spectral example (bottom), analogous relationships link spectral and molecular domains. Orange highlights the main entities; blue and green denote related components. This analogy is purely conceptual — unlike deterministic linguistic tokens, NMR signals are subject to noise, overlap, strong coupling, and instrumental distortions, making the spectral inverse problem ill-posed (see Methods in the main text).

2 MolDeTr: Inside the Architecture and Training Strategies of a Transformer-Based Analysis Framework

2.1 Network Architecture

2.1.1 1D Feature Pyramid Network Backbone for Multi-Scale Spectral Feature Extraction

We employ a 1D Feature Pyramid Network (FPN) as the backbone of MolDeTr. The FPN architecture comprises both bottom-up and top-down pathways. The bottom-up pathway progressively downsamples the input NMR spectrum through successive convolutional layers, while the top-down pathway performs upsampling at each corresponding layer. This dual-pathway design creates a pyramid-like hierarchy of feature maps, enabling the model to capture multi-scale spectral features effectively. Residual connections between corresponding layers of the bottom-up and top-down pathways preserve high-resolution information, preventing its loss during processing. Specifically, our 1D FPN consists of 11 residual blocks [10], each employing varying kernel sizes of $k \in \{3, 5, 7, 9\}$ and a total of 256 channels (64 per kernel size) in the bottom-up path. The top-down path includes 11 upsampling layers, each integrating features from the bottom-up pathway via residual connections to enhance feature representation.

2.1.2 Transformer Encoder-Decoder with Deformable Attention and Iterative Refinement

The transformer component of MolDeTr is similar to the original DeTr model but is adapted for one-dimensional spectral data by utilizing one-dimensional positional embeddings, refer to Figure 1b in main manuscript. This

adaptation ensures that the transformer effectively processes the sequential nature of NMR spectra without altering its core architecture. Our transformer implementation follows Zhu et al. (Deformable DETR), adapted from two-dimensional spatial features to one-dimensional spectral features. The principal modification is that positional embeddings and deformable sampling operate along the one-dimensional chemical-shift axis rather than a two-dimensional spatial grid. All other architectural components follow the reference. The complete parameter specification is as follows. We incorporate multi-scale deformable attention and iterative parameter refinement as introduced by Zhu et al. [9], alongside the Group DeTr approach to improve decoder training [11]. In the decoder, ten spin queries were utilized for each of the 8 decoder query groups (following the Group-DETR training strategy of Chen et al.; not to be confused with chemically or magnetically equivalent spin groups in the NMR sense). We adopt Group-DeTr to accelerate training; at inference we use the first group only. Hence, per input spectrum window we effectively predict up to 10 different equivalent-spin groups. The remaining groups assist training efficiency. The equivalent-spin group capacity is scalable with compute. In addition, six layers of self-attention are integrated following the cross-attention mechanism to facilitate direct correlation between spins. The transformer features a hidden dimension of 256, with each encoder and decoder layer comprising 8 attention heads. The model includes 6 encoder layers and 6 decoder layers, with deformable attention spanning 4 levels and utilizing 4 points per level, enhancing the model's capacity to attend to relevant spectral features across multiple scales.

2.2 Coupling Constant Prediction Mechanism

Accurate prediction of coupling constants is essential for the precise interpretation of NMR spectra. In MolDeTr, each spin (node) within the spin-system graph is associated with a set of coupling constants shared with its coupled spins. Given the inherently unordered nature of these couplings, enforcing a specific sequence can artificially inflate prediction errors. To address this, MolDeTr directly predicts permutation-invariant embeddings of the coupling constants, thereby more reliably capturing the intrinsic relationships among spins without imposing an arbitrary order.

Why Ordering Can Be Problematic. Consider a node with true coupling constants:

$$C_{\text{true}} = \{1.5\,\text{Hz},\ 2.5\,\text{Hz},\ 7.5\,\text{Hz},\ 16.7\,\text{Hz}\}.$$

Suppose the model predicts:

$$C_{\text{pred}} = \{2.5\,\text{Hz},\ 7.5\,\text{Hz},\ 16.7\,\text{Hz}\},$$

thereby *missing* the smallest coupling constant, 1.5 Hz.

Ordered Alignment Error: Imposing a strict ordering (e.g., sorting from smallest to largest) results in:

$$\text{Ground Truth (sorted): } [1.5,\ 2.5,\ 7.5,\ 16.7],$$
$$\text{Prediction (sorted): } [2.5,\ 7.5,\ 16.7].$$

Calculating the position-wise absolute errors:

$$\begin{aligned}
&\text{Position 1: } |1.5 - 2.5| = 1.0\,\text{Hz},\\
&\text{Position 2: } |2.5 - 7.5| = 5.0\,\text{Hz},\\
&\text{Position 3: } |7.5 - 16.7| = 9.2\,\text{Hz},\\
&\text{Position 4: Missing prediction for } 16.7\,\text{Hz}.
\end{aligned}$$

The cumulative error sums to $1.0 + 5.0 + 9.2 = 15.2\,\text{Hz}$, excluding any penalty for the missing coupling. Despite the prediction being near-correct for three of the four couplings, the rigid index-by-index comparison disproportionately penalizes the omission, leading to an inflated error metric.

Permutation-Invariant Embedding Error: In contrast, MolDeTr directly predicts the embedding of the coupling constants:

$$E_{\text{pred}} = E(C_{\text{pred}}) = \left[\min(C_{\text{pred}}),\ \max(C_{\text{pred}}),\ \text{mean}(C_{\text{pred}}),\ \sum(C_{\text{pred}})\right] = [2.5,\ 16.7,\ 8.90,\ 26.7].$$

The ground truth embedding is:

$$E_{\text{true}} = E(C_{\text{true}}) = \left[\min(C_{\text{true}}),\ \max(C_{\text{true}}),\ \text{mean}(C_{\text{true}}),\ \sum(C_{\text{true}})\right] = [1.5,\ 16.7,\ 7.05,\ 28.2].$$

Calculating the absolute differences:

$$\begin{aligned}
&|\min(C_{\text{true}}) - \min(C_{\text{pred}})| = |1.5 - 2.5| = 1.0\,\text{Hz},\\
&|\max(C_{\text{true}}) - \max(C_{\text{pred}})| = |16.7 - 16.7| = 0\,\text{Hz},\\
&|\text{mean}(C_{\text{true}}) - \text{mean}(C_{\text{pred}})| = |7.05 - 8.90| = 1.85\,\text{Hz},\\
&|\sum(C_{\text{true}}) - \sum(C_{\text{pred}})| = |28.2 - 26.7| = 1.5\,\text{Hz}.
\end{aligned}$$

The total embedding error is $1.0 + 0 + 1.85 + 1.5 = 4.35\,\text{Hz}$, which is significantly lower than the error incurred by the ordered alignment approach. Notably, the permutation-invariant method effectively captures the most critical aspects of the coupling set (minimum, maximum, mean, and total sum) while minimizing the impact of missing or slightly altered couplings. Because the number of couplings $|C|$ varies from spin to spin, we report both mean and $\sum$ alongside $\min$ and $\max$ to retain information that would otherwise be lost when $|C|$ changes. In practice, our core evaluations emphasize $\max(J)$, which typically carries the strongest structural and interpretive relevance and is most reliably predicted. For selected showcase cases, we demonstrate full recovery of the entire coupling set C, but recovering more than three distinct couplings with the current four-dimensional embedding turned out to be difficult. We therefore do not seek exact recovery of C from $E(C)$ in the general evaluation protocol.

Formal Description of the Embedding. To circumvent the drawbacks of ordered alignment, we treat each node's coupling constants

$$C = \{c_1, c_2, \ldots, c_n\}$$

as an *unordered set* and embed them using permutation-invariant functions:

$$E(C) = \left[\min(C),\ \max(C),\ \text{mean}(C),\ \sum(C)\right].$$

For example, if

$$C = \{2.5\,\text{Hz},\ 7.5\,\text{Hz},\ 16.7\,\text{Hz}\},$$

then

$$E(C) = [2.5,\ 16.7,\ 8.90,\ 26.7].$$

This embedding succinctly captures essential statistical properties—such as the minimum, maximum, mean, and total sum—without enforcing any arbitrary order among the coupling constants.

Predictive Usage and Flexibility:

In the majority of analyses, we focus on the largest coupling constant ($\max$), which often carries significant structural or interpretive relevance. Nonetheless, we can incorporate $\min$ or $\sum$ to capture more detailed aspects of the coupling set. *By treating couplings as permutation-invariant embeddings, we bypass another layer of bipartite matching (i.e., nested bipartite matching in the MolDeTr model).* Such nested bipartite matching is computationally expensive and slows down training significantly.

Inverse Problem:

To recover the final set of coupling constants from the predicted embeddings, we solve:

$$\hat{C} = E^{-1}(E_{\text{output}}),$$

where E^{-1} is an inverse mapping that reconstructs the coupling-constant set.

By leveraging this permutation-invariant embedding approach, MolDeTr robustly models coupling constants without relying on an ordering scheme—thus minimizing error propagation and reducing computational overhead, even when predictions would differ in quantity or when couplings are nearly identical in value.

2.3 Optimized Training Protocols and Resampling Strategies

2.3.1 Training Protocol

MolDeTr was trained using the Rectified Adam (RAdam) optimizer [12], chosen for its ability to enhance convergence stability by mitigating issues associated with suboptimal local minima that often plague standard optimizers like Adam

[13]. RAdam integrates adaptive learning rates with rectification mechanisms, thereby improving training stability and overall performance.

2.3.2 Training Parameters

- **Learning Rate**: Initialized at 1×10^{-4}.
- **Batch Size**: 32.
- **Learning Rate Scheduling**: The learning rate is reduced by half upon observing a plateau in validation error, facilitating finer adjustments as training progresses.
- **Projection Layers**: These layers are trained with a learning rate that is 10% of the main network's rate, ensuring gradual fine-tuning and stable integration with the backbone and transformer components.
- **Epochs**: The model has trained over 150 epochs, balancing comprehensive data exposure with computational efficiency.

2.3.3 Dataset and Preprocessing

The training dataset comprised 5 million simulated NMR spectra, each digitized at a resolution of 6144 pixels across a 1200 Hz range, resulting in a sampling rate of 5.12 points per Hz. This high digital resolution data enables MolDeTr to capture intricate spectral details essential for precise parameter prediction. All spectral intensities were normalized to a [0, 1] range, eliminating assumptions regarding total proton counts and ensuring uniform scaling across diverse spectral features.

2.3.4 Normalization and Loss Weighting

To maintain consistency across different spectral parameters, such as chemical shifts and coupling constants, all parameters were normalized within the [0, 1] interval. Within the L^1 loss function, coupling constants were assigned a weight of 2, while chemical shifts received a weight of 32. These empirically determined weights ensure a balanced influence of both classification and regression tasks during training, promoting accurate and reliable parameter predictions.

2.3.5 Computational Resources and Scalability

Training was conducted on two Nvidia V100 GPUs, each with 32 GB of memory, allowing the model to reach convergence in approximately 300 GPU hours. The architecture of MolDeTr is inherently scalable and capable of handling spectra with wider ranges and a larger number of magnetically equivalent spins. Leveraging the DeTr framework's proficiency in managing extensive inputs and comprehensive prediction sets [14], MolDeTr is well-equipped to further expand its analytical capabilities as computational resources and dataset complexities increase.

2.3.6 Resampling Strategy

To ensure uniform processing across spectra acquired at varying field strengths, all input data were resampled to a fixed resolution of 5.12 points per Hz. This standardization guarantees that MolDeTr maintains consistent analytical performance regardless of the original spectrometer's base frequency, thereby preserving the quality and reliability of spectral analyses across diverse experimental conditions.

3 Modular Quantum Mechanical Spin System Simulations for High-Fidelity NMR Spectral Data

High-quality, consistently labeled, and large-scale datasets are essential for advancing ML applications across various spectral techniques, including NMR, MS, and IR [15, 16]. Accurately simulating spectral data addresses challenges associated with experimental data acquisition and ensures that ML models are trained on realistic and representative datasets. A critical component of this effort in NMR spectroscopy is the precise quantum mechanical modeling and simulation of spin systems, which serve as modular building blocks for generating comprehensive NMR spectra. By constructing complex molecular spin systems from these basic modules and leveraging their independent and parallel simulation, we achieve significant improvements in computational efficiency and speed. Additionally, incorporating realistic spectral distortions bridges the gap between synthetic and experimental data, enhancing the robustness and generalizability of ML models. This section outlines our systematic approach to defining, assembling, and simulating spin system building blocks, with a focus on coupling regimes and spectral distortions to produce high-fidelity NMR spectral data for advanced analytical applications.

3.1 Modular Spin System Building Blocks

To accurately replicate the complexities and structural details of real NMR spectra, we decompose intricate spin systems into smaller, manageable building blocks. These basic modules are categorized based on their coupling regimes—**strongly coupled**, **weakly coupled**, and **uncoupled**—ensuring that each building block captures distinct aspects of spin interactions prevalent in various molecular environments. This modularity allows for independent and parallel simulation of each building block, significantly enhancing computational efficiency and scalability.

3.1.1 Classification by Coupling Regimes

Creating representative spectra that encompass all coupling regimes relies on balancing the creation of spin systems in different coupling regimes. We classify coupling strength using the dimensionless ratio $\left|\frac{\Delta\delta}{J}\right|$, where $\Delta\delta$ is the chemical shift difference between coupled nuclei and J is the coupling constant:

$$\left|\frac{\Delta\delta}{J}\right| = \frac{|\delta_A - \delta_B|}{J}$$

Using a threshold value of 10, we define the coupling regimes as follows:

- **Weakly Coupled**: $\left|\frac{\Delta\delta}{J}\right| > 10$. The chemical shift difference significantly exceeds the coupling constant, resulting in distinct and easily resolvable multiplet patterns. At this ratio, coupling effects are minimal, and first-order approximations are generally sufficient.

- **Strongly Coupled**: $\left|\frac{\Delta\delta}{J}\right| \leq 10$. The chemical shift difference is comparable to or smaller than the coupling constant, leading to complex splitting patterns that may overlap and require sophisticated analysis. Coupling effects are significant and cannot be neglected.
- **Uncoupled**: Represented by single or symmetrically equivalent spins with negligible coupling, producing singlet signals in NMR spectra.

Although we did not find a universally accepted threshold value in the literature, we chose a threshold of 10 based on empirical observations and practical considerations in NMR spectroscopy. When the ratio $\left|\frac{\Delta\delta}{J}\right|$ exceeds 10, the coupling effects become less significant, and the spectra can often be interpreted using first-order approximations. This threshold allows us to effectively categorize the coupling regimes for the purpose of creating representative spectra.

3.1.2 Building Block Catalog

Each building block represents a minimal spin system characterized by specific coupling interactions and chemical shifts within a defined coupling regime. Table S2 categorizes these modules by coupling regime, providing representative functional groups or molecular fragments and their analytical relevance in one-dimensional proton NMR spectroscopy [17, 18].

Table S2: Modular spin system building blocks categorized by coupling regimes, with representative functional groups and molecular fragments.

Coupling Regime	Building Block	Examples and Relevance
**Strongly Coupled**	AB, ABX, AB_2, ABC, AA′BB′, AA′BB′C, AA′BB′X, A_2B, A_3B, A_2B_2, A_3B_2	**ABX**: 1-Bromo-2-chloropropane ($BrCH_2CHClCH_3$) – Two methylene protons (H_A, H_B) strongly coupled to each other and weakly coupled to the adjacent CH proton (H_X). Common in asymmetric alkyl halides.<br>**AA′BB′**: 1-Bromo-2-chloroethane ($BrCH_2CH_2Cl$) – Two inequivalent methylene groups ($H_A, H_{A'}$ and $H_B, H_{B'}$) coupled within and between groups. Important for studying geminal and vicinal coupling.<br>**ABC**: Vanillin ($C_8H_8O_3$) – Three aromatic protons (H_A, H_B, H_C) with strong coupling, observed in substituted aromatic compounds.<br>**AA′BB′C**: System with AA' and BB' pairs strongly coupled to each other and to C, such as in the aromatic system of cinnamic acid.
**Weakly Coupled**	AX, AMX, AA′XX′, AA′MM′X, A_2X, A_3X, A_3X_2, A_2X_2, AM_2X	**A_2MXY**: Allyl chloride ($CH_2 = CH–CH_2Cl$) – The two terminal vinyl protons are *cis*/*trans*-inequivalent (H_M, H_X); with the internal vinyl proton (H_Y) they form a three-spin vinyl pattern, while the two equivalent CH_2Cl protons form the H_{A_2} group. Useful for unsaturated compounds.<br>**A_3X_2**: Ethanol (CH_3CH_2OH) – The methyl protons (H_{A_3}) weakly couple to the methylene protons (H_{X_2}). Fundamental for alcohol analysis in metabolomics.
**Uncoupled**	A, A_2, A_3, A_4, A_6	**A_3**: Methyl protons of nitromethane (CH_3NO_2) – The three protons (H_A) are chemically and magnetically equivalent, appearing as a singlet. Common in solvents and small molecules.<br>**A_6**: Benzene (C_6H_6) – The six aromatic protons (H_A) are chemically and magnetically equivalent, producing a sharp singlet due to high symmetry. A benchmark in NMR analysis.

We have selected these examples to illustrate both the analytical relevance and the potential challenges associated with interpreting different spin systems. Strongly coupled systems like $AA'BB'$ exhibit complex multiplet structures that require advanced analysis techniques, which is essential for elucidating detailed structural information in stereochemically rich environments such as benzene rings. Weakly coupled systems such as A_3X_2 display simpler splitting patterns that are fundamental in structural identification of common functional groups like ethyl groups. Uncoupled systems, represented by nuclei that do not experience significant coupling, produce singlet peaks that are critical in recognizing certain functional groups or symmetrical structures.

3.1.3 Integration into Complex Spin Systems

By systematically combining the defined building blocks, we can model the full range of coupling interactions present in real molecules. This modular approach ensures efficient and scalable simulations of complex spin systems from diverse chemical environments. For example, complex aromatic systems can be constructed by integrating strongly coupled modules like ABX and $AA'BB'$. At the same time, aliphatic chains can be modeled using weakly coupled modules such as AX and A_3X_2.

The resulting spectra illustrate the complex interactions within the experimental data, offering a thorough representation of solid training data for machine learning models. This, in turn, enhances their predictive performance in spectral analysis.

3.2 Simulating Diverse NMR Spectra: Methodology and Parameterization

To generate realistic synthetic NMR spectra, we meticulously simulate a wide range of spin systems, encompassing strongly coupled, weakly coupled, and uncoupled systems. This diversity ensures comprehensive coverage of spectral features encountered in real-world samples, enhancing the robustness and generalizability of ML models trained on this data.

3.2.1 Simulation Framework

Our simulation framework models individual spin system building blocks independently, leveraging their small size ($N < 6$) to enable parallel processing. This modular approach eliminates the need for clustering or approximation methods typically required for larger spin systems. Each building block is simulated on separate computational threads or processors, ensuring high spectral realism while significantly improving computational efficiency and speed.

3.2.2 Spin System Generation and Parameter Sampling

The generation of synthetic spectra involves creating diverse spin systems and sampling critical parameters such as chemical shifts, coupling constants, and line widths within realistic ranges to reflect typical NMR conditions:

- **Chemical Shifts**: Uniformly sampled from 0 to 15 ppm (0 to 1200 Hz on an 80 MHz spectrometer).

- **Coupling Constants**: Uniformly sampled between 0.01 Hz and 20 Hz, covering typical scalar coupling interactions in small to medium-sized molecules.
- **Line Widths**: Uniformly sampled between 0.3 Hz and 2.2 Hz, capturing peak broadening due to molecular dynamics.

Each spin system is treated as an independent entity during simulation, allowing precise control over spectral features and enabling efficient parallel computation.

3.2.3 Efficient Spectrum Simulation through Block Diagonalization

To efficiently simulate the NMR spectra of small spin systems, we employ block diagonalization techniques [19, 20, 21]. The Hamiltonian operator for N nuclei in an external magnetic field oriented along the z-axis is given by:

$$\mathcal{H} = \sum_{i=1}^{N} \delta_i I_{z,i} + \sum_{i<j} J_{ij} \mathbf{I}_i \cdot \mathbf{I}_j$$

where:

- δ_i is the chemical shift of nucleus i,
- J_{ij} is the scalar coupling constant between nuclei i and j,
- $I_{z,i}$ is the z-component of the angular momentum operator for nucleus i,
- $\mathbf{I}_i$ and $\mathbf{I}_j$ are the **spin angular momentum vector operators** for nuclei i and j, respectively. Each vector operator comprises Cartesian components $I_{x,i}$, $I_{y,i}$, $I_{z,i}$ and $I_{x,j}$, $I_{y,j}$, $I_{z,j}$, which satisfy the standard angular momentum commutation relations:

  $$[I_{a,i}, I_{b,j}] = i\hbar\delta_{ij}\epsilon_{abc}I_{c,i}$$

  where $a, b, c \in \{x, y, z\}$, δ_{ij} is the Kronecker delta, and ϵ_{abc} is the Levi-Civita symbol.

A significant challenge in quantum mechanical simulations is the exponential growth of the Hamiltonian matrix with the number of spins. However, because the Hamiltonian commutes with the **total I_z operator** ($I_{z,\text{total}} = \sum_{i=1}^{N} I_{z,i}$), $\mathcal{H}$ can be decomposed into smaller, independent blocks. This block diagonalization approach substantially reduces computational complexity by enabling the diagonalization of each block separately.

In simple single-pulse experiments, such as standard one-dimensional proton NMR spectra, explicit modeling of relaxation processes is often unnecessary [19]. Instead, the spectrum is constructed from the **resonance frequencies** and **intensities** derived from the eigenvalues and eigenstates of the stationary Schrödinger equation:

$$\mathcal{H}\Psi = E\Psi$$

Here, each eigenvalue E corresponds to a specific energy level, translating to a resonance frequency $\nu_i = (E_i - E_{\text{ref}})/h$, where E_{ref} is a reference energy and h is Planck's constant. The corresponding eigenstates Ψ_i determine the transition intensities I_i based on their population distributions and transition dipole moments.

The simulated spectrum $S(\nu)$ is generated by summing **Lorentzian line shapes** centered at the resonance frequencies ν_i with intensities I_i and line widths Γ_i:

$$S(\nu) = \sum_i \frac{I_i}{1 + \left(\frac{\nu - \nu_i}{\Gamma_i}\right)^2}$$

This quantum mechanical model accurately captures complex NMR spectra, including those with extensive spectral line patterns, by efficiently simulating interactions within small, independently simulated spin blocks. This approach enhances scalability, allowing for the practical simulation of systems with a considerable number of spins without prohibitive computational costs [3].

3.3 Generating and Enhancing NMR Spectra: Examples and Distortions

To validate our simulation framework and ensure the generation of realistic NMR spectra, we present examples of simulated spectra, both pristine and with realistic distortions.

3.3.1 Simulated Spectra Without Distortions

Figures S4 and S5, along with Table S3, show simulated NMR spectra without distortions, highlighting diverse coupling behaviors across various spin systems. In symmetric systems like `AA′BB′`, chemically equivalent but magnetically non-equivalent spins are treated as single entities with additional coupling interactions due to similar coupling constants.

In Table S3, several abbreviations are used to concisely present the data. These abbreviations are defined as follows:

- **# P**: Proton Counts
- **Shift**: Chemical Shift
- **LW**: Line Width (Inverse proportional to Transverse Relaxation Time)
- **SRW**: Signal Region Width
- **Coupling**: Coupling Constant

The **signal region** of a nucleus in an NMR spectrum is the range of frequencies or chemical shifts where its peaks appear, determined by the coupling constants with all coupled nuclei. It accounts for the splitting pattern caused by direct and indirect spin-spin interactions, with the total width calculated as the sum of all coupling contributions. For example, in an AM_2X system, the signal region of A spans the combined splitting from M_2 (a triplet due to J_{AM}) and X (a doublet from J_{AX}), resulting in a total region width of $2 \cdot J_{AM} + J_{AX}$.

Table S3: Details of Simulated Spectra (1-6)

Spectrum	Signal	# P	Shift (Hz)	LW (Hz)	SRW (Hz)	Coupling (Hz)
**1**	1	1	51.28	2.05	5.56	5.56
	2	3	234.10	2.15	16.67	5.56
**2**	1	1	625.86	1.53	16.82	4.26, 18.77
	2	1	648.86	2.18	19.02	4.26, 12.57
	3	1	864.81	0.53	19.02	19.02
	4	1	1063.84	0.35	23.02	19.02
	5	1	1075.85	1.43	31.33	12.57, 18.77
**3**	1	1	185.63	1.79	6.72	2.89, 12.32
	2	1	240.89	0.40	15.21	3.83, 12.32
	3	1	306.70	1.41	16.15	2.89, 3.83
**4**	1	3	263.82	0.31	25.12	12.56
	2	2	278.50	0.65	37.68	12.56
**5**	1	2	362.63	2.16	13.79	0.95, 5.38, 7.47
	2	2	370.26	1.16	26.65	0.95, 5.38, 15.90
	3	1	433.45	0.84	46.75	7.47, 15.90
**6**	1	1	239.93	1.88	28.75	3.66, 12.55
	2	1	415.61	0.73	28.56	9.61, 18.95
	3	1	500.99	1.30	19.97	1.02, 18.95
	4	1	504.83	1.37	10.63	1.02, 9.61
	5	2	586.19	1.33	31.58	12.55, 19.03
	6	1	707.22	1.12	41.72	3.66, 19.03

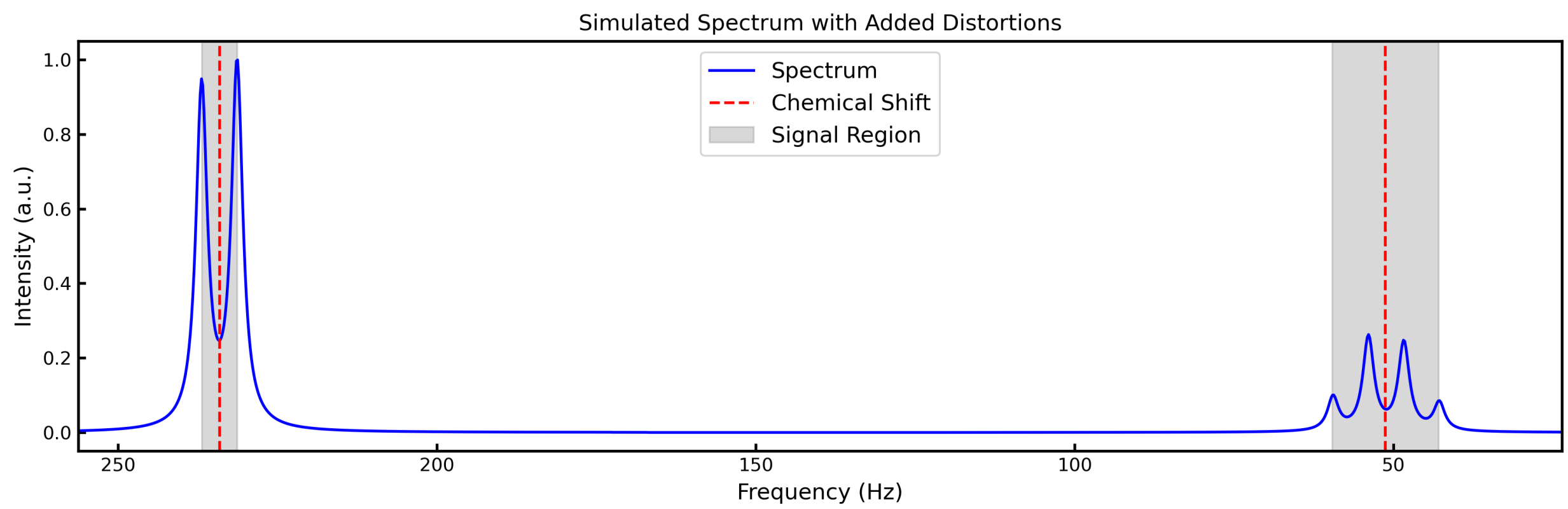

(a) Spectrum 1

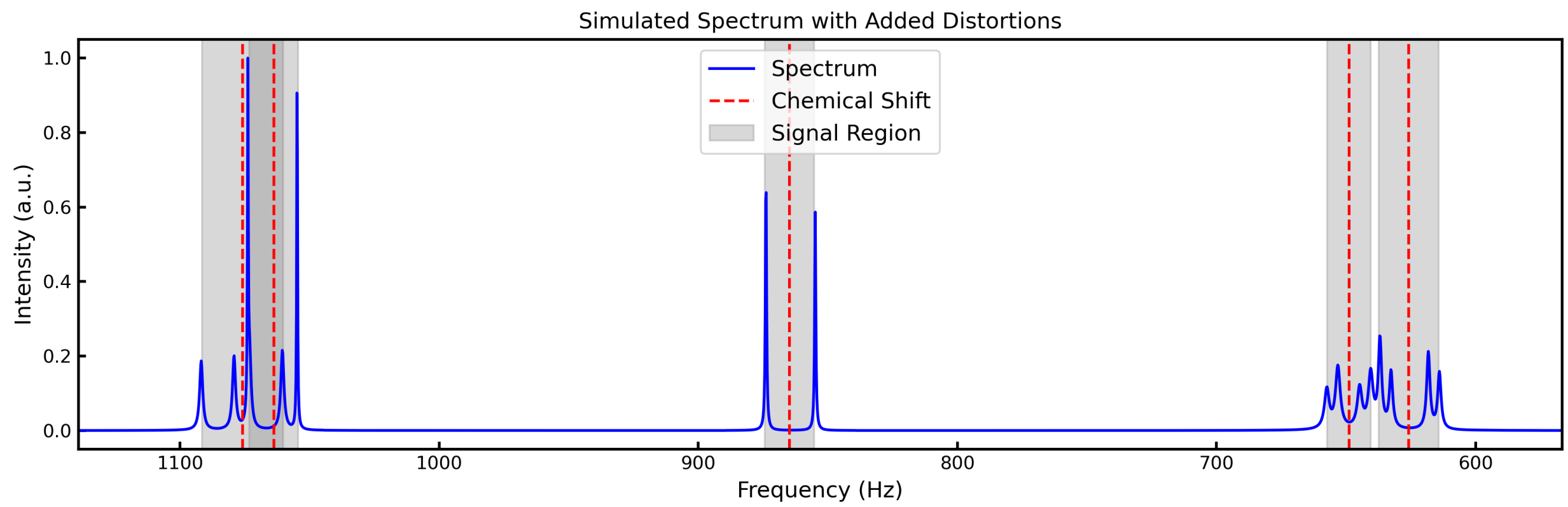

(b) Spectrum 2

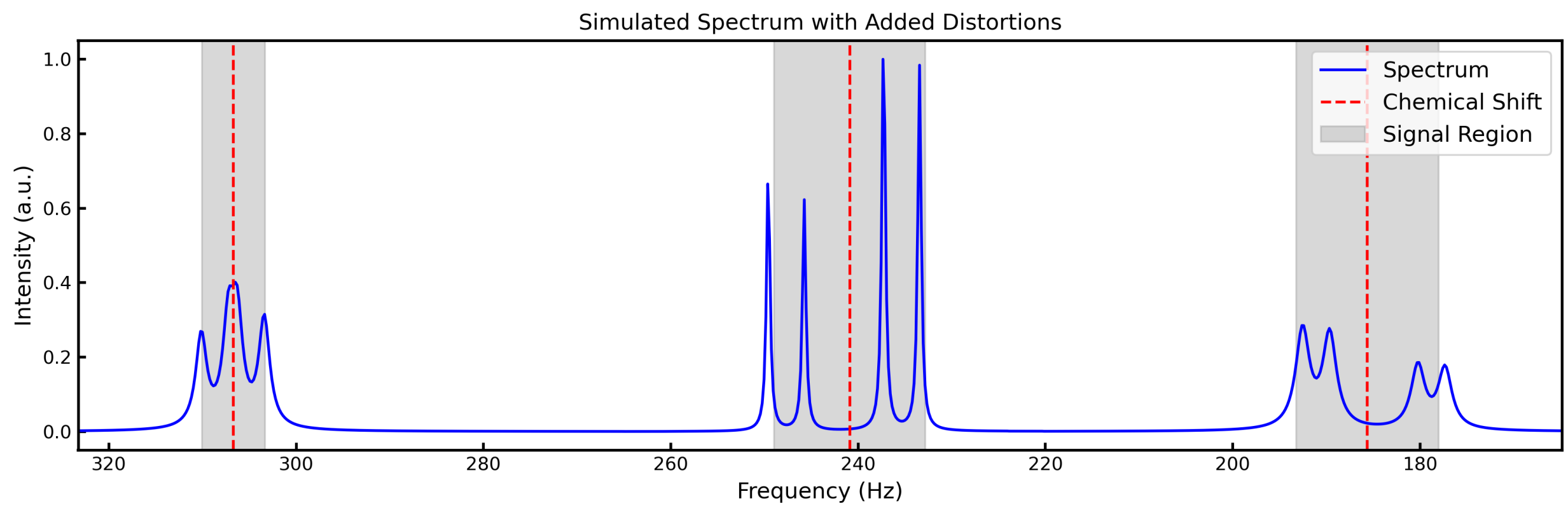

(c) Spectrum 3

Figure S4: Examples of simulated spectra without distortions (1-3)

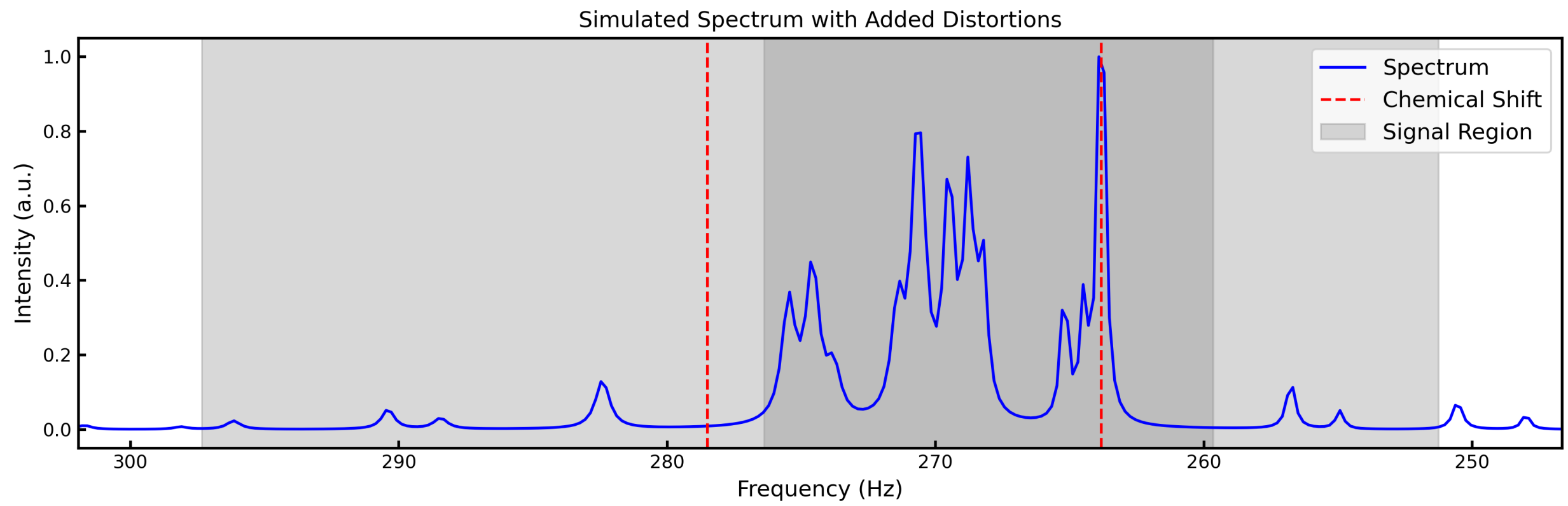

(a) Spectrum 4

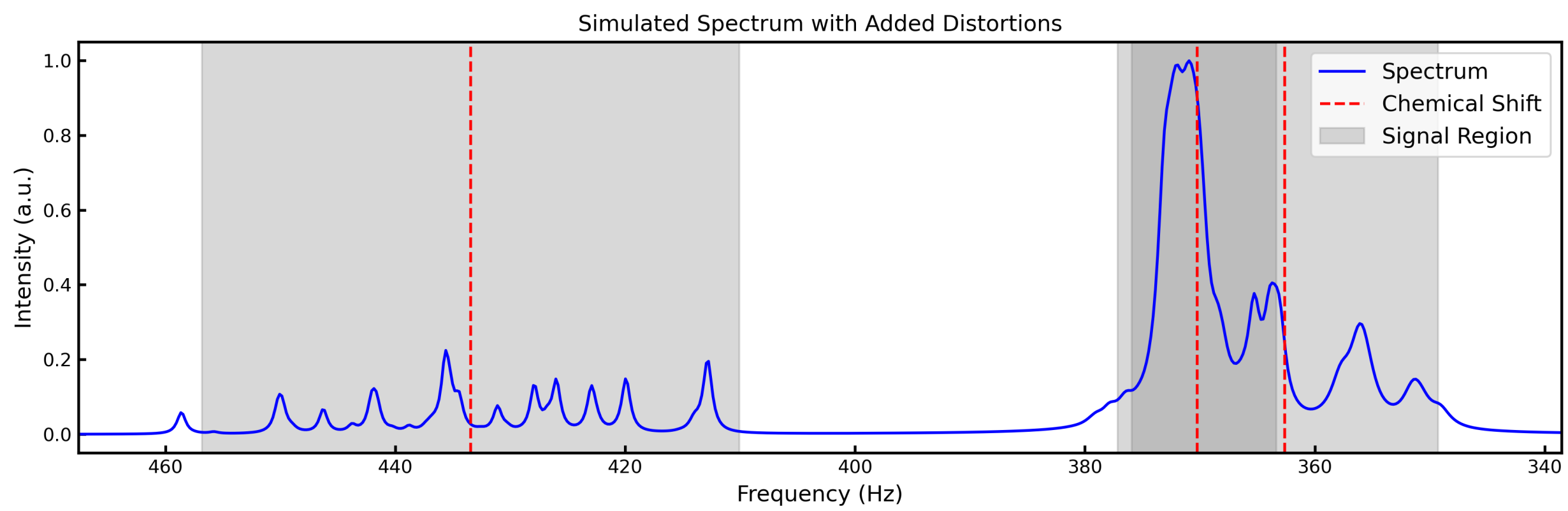

(b) Spectrum 5

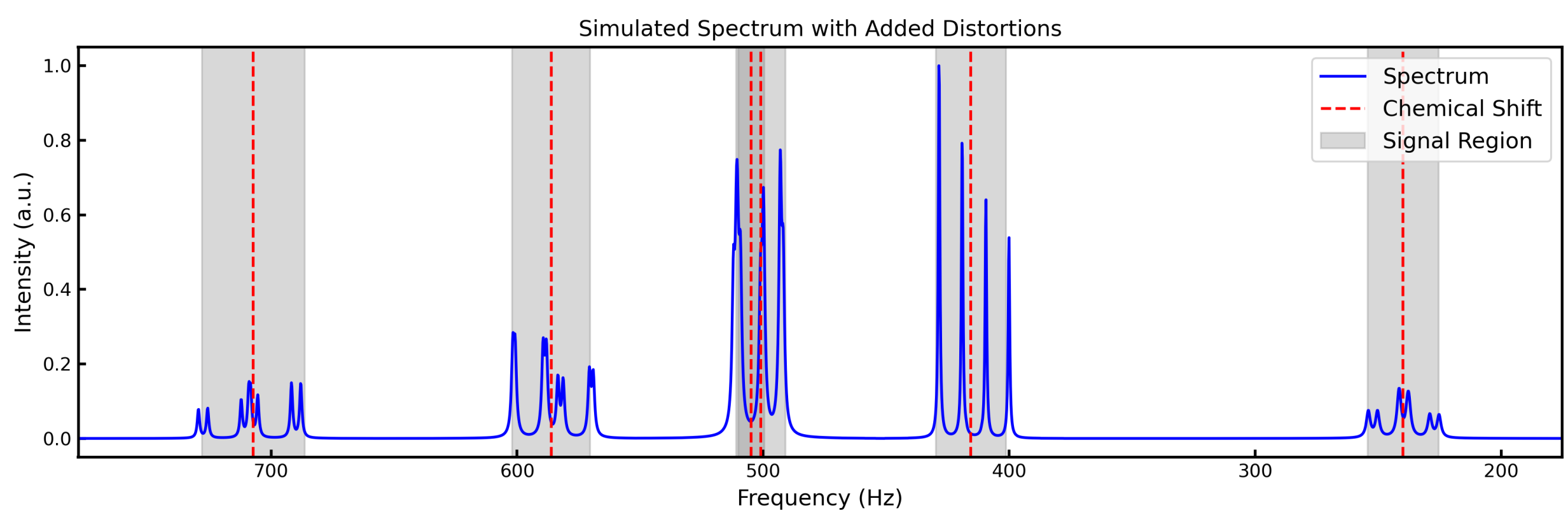

(c) Spectrum 6

Figure S5: Examples of simulated spectra without distortions (4-6)

3.3.2 Introducing Realistic Spectral Distortions

To ensure that synthetic spectra closely resemble experimental NMR data, we introduce realistic distortions that replicate inherent experimental imperfections. These distortions enhance the training data, enabling ML models to generalize effectively to practical scenarios.

Types of Distortions The distortions applied to synthetic spectra include:

- **^{13}C Satellites**: Minor peaks from the natural abundance of ^{13}C isotopes, introducing additional spectral lines.
- **Shimming Distortions**: Variations in magnetic field homogeneity, simulated via SHIMpanzee [22], affecting line shape and peak symmetry.
- **Phase Distortions**: Shifts in the phase of the NMR signal, leading to asymmetric peak shapes.
- **Gaussian Noise**: Additive noise of varying intensities to simulate signal noise, affecting the SNR.
- **Baseline Variations**: Fluctuations in the baseline signal to mimic instrument and sample imperfections, introducing baseline drifts and offsets.

Application of Distortions During each training epoch, distortions are randomly applied to synthetic spectra, ensuring that ML models encounter a wide range of spectral irregularities. This randomness prevents data repetition and enhances the model's ability to generalize to unseen experimental data.

Figures S6 and S7 illustrate the sequential application of distortions to two different spectra. The top panels display the original (simulated) spectra, followed by panels showing spectra with added distortions. This approach equips the MolDeTr model to handle real-world spectral irregularities, thereby improving its predictive reliability and generalization capability.

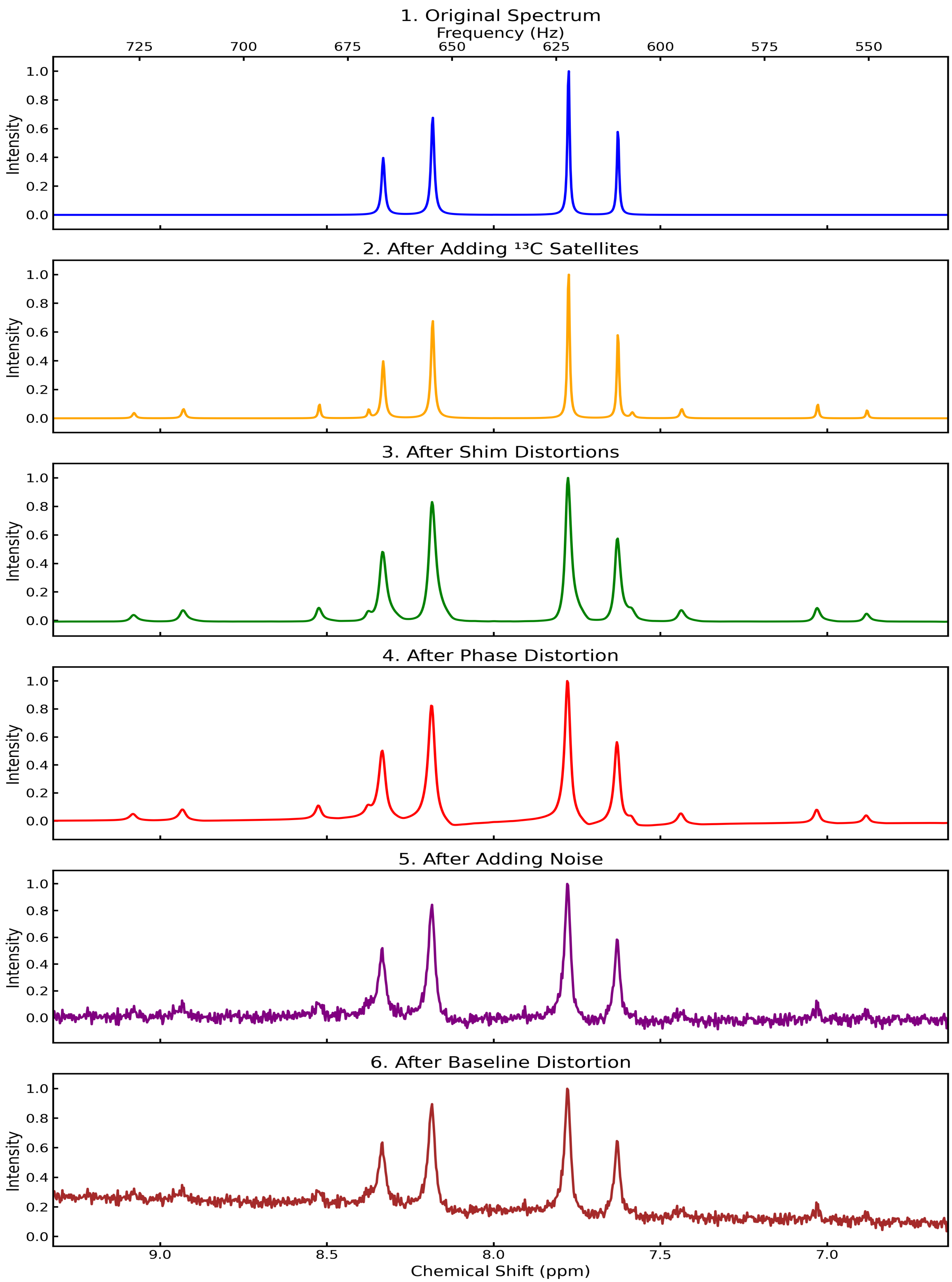

Figure S6: First NMR spectrum: the original (simulated) spectrum is progressively distorted by adding ^{13}C satellites, shimming distortions, phase distortions, Gaussian noise, and baseline distortions.

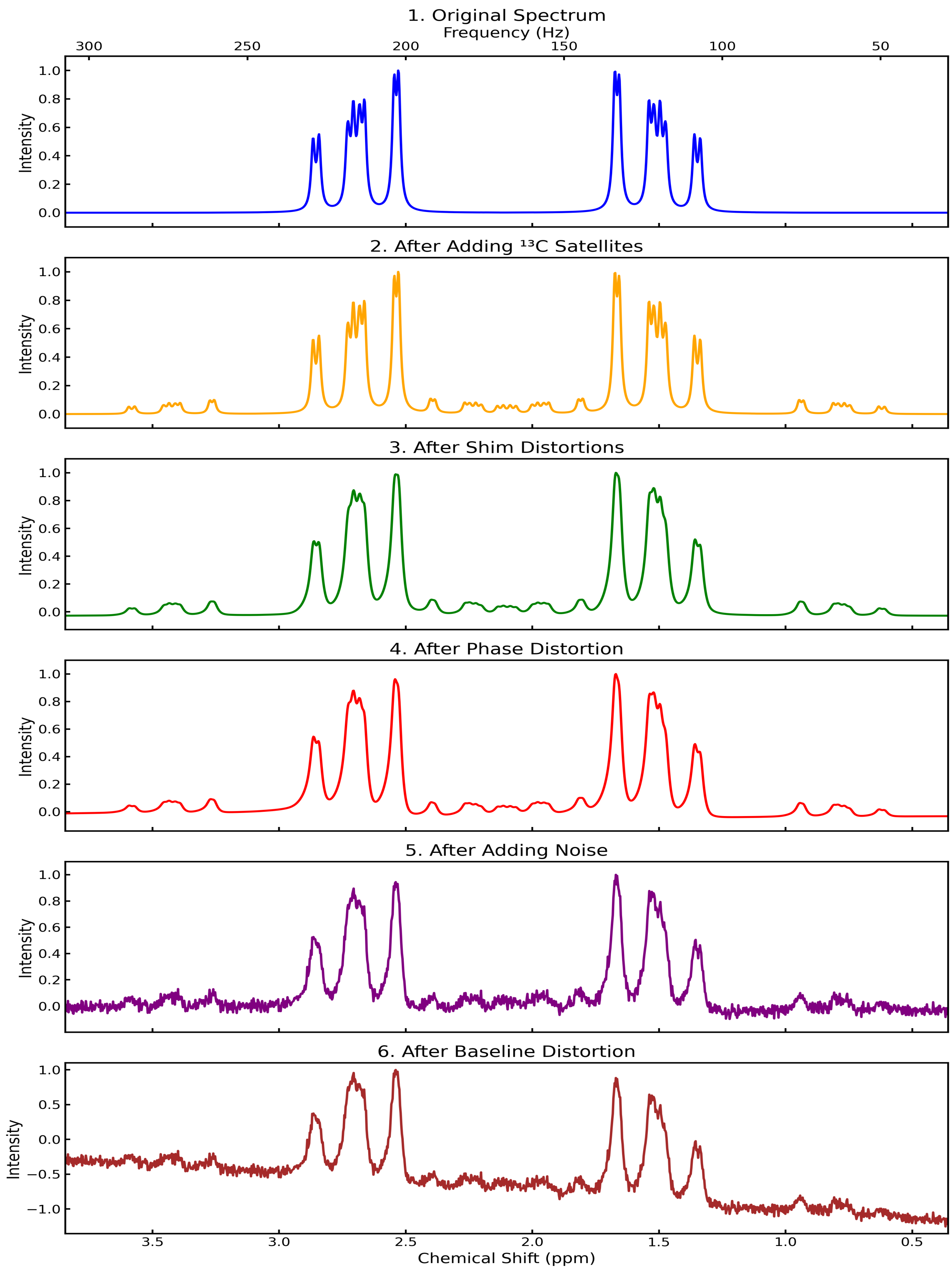

Figure S7: Second NMR spectrum: the original (simulated) spectrum is sequentially distorted with ^{13}C satellites, shimming distortions, phase distortions, Gaussian noise, and baseline distortions.

3.4 Analytical Relevance

The precise simulation of diverse molecular spin systems, combined with the incorporation of realistic distortions, facilitates their application across multiple analytical domains where difficult spectra and intricate spin systems are critical. Key applications include:

- **Molecular Structure Elucidation**: Accurate identification of spin systems aids in determining molecular connectivity and stereochemistry, essential for elucidating the structures of unknown compounds [23].
- **Metabolomic Profiling**: Diverse spin systems enable comprehensive profiling of metabolites, supporting biomarker discovery and metabolic pathway analysis [24, 25].
- **Pharmaceutical Development**: Understanding spin systems in drug candidates assists in optimizing molecular interactions and assessing conformational stability, enhancing drug design processes [26].
- **Quality Assurance**: Reliable spin system verification ensures product purity and detects adulterants in pharmaceutical and materials science industries [27].
- **Reaction Mechanism Studies**: Monitoring changes in spin systems over time provides insights into reaction mechanisms and kinetics, elucidating the underlying processes of chemical transformations [28].

The principles underlying our data creation pipeline—combining precise simulations of physical and chemical phenomena with realistic distortions—are broadly applicable to other spectral techniques, including MS and IR. This robust framework for generating high-fidelity spectral data empowers the broader spectral science community to develop advanced machine learning models that deliver reliable and insightful analytical outcomes.

4 Detailed Results

4.1 Extended Results on Multiple Independent Spin Systems

This section provides an extended analysis of multiple independent spin systems, building upon the Results section. Figures S8, S9, and S10 present evaluation metrics for these systems, derived from distorted simulated data. Spectra 2 and 6 from SI Section 3.2.1 serve as representative examples.

As presented in Table S4, the performance metrics for line widths and signal region widths in multiple independent spin systems exhibit strong correlations, with coefficients of determination (R^2) of 0.881 and 0.975, respectively. The low mean absolute error (MAE) and median absolute error values indicate high precision in the estimations, where line widths achieve an MAE of 0.101 Hz and a median error of 0.045 Hz, while signal region widths exhibit an MAE of 0.800 Hz and a median error of 0.240 Hz. These results underscore the accuracy and reliability of the measurements. Furthermore, the line width (Γ) is inversely related to the transverse relaxation time (T_2) through the formula:

$$T_2 = \frac{1}{\pi\,\Gamma}$$

This relationship implies that smaller errors in line width estimation (Γ) correspond to smaller errors in T_2 estimation. For example, a line width of 1 Hz results in a transverse relaxation time of approximately $T_2 = \frac{1}{\pi \times 1\,\text{Hz}} = 318\,\text{ms}$. Therefore, precise estimation of line widths is crucial for accurately determining T_2, which is essential for understanding the dynamics of spin systems.

Table S4: Performance for multiple independent spin systems.

	**Line Widths**	**Signal Region Widths**
**Coefficient of Determination (R^2)**	0.881	0.975
**Mean Absolute Error (Hz)**	0.101	0.800
**Median Absolute Error (Hz)**	0.045	0.240

Figures S8a and S8b present the regression results for chemical shift predictions. The scatter plot in Figure S8a demonstrates a strong agreement between predicted and ground truth chemical shifts, indicating high model accuracy. The residuals in Figure S8b show small deviations, confirming the consistency and reliability of the predictions.

The line width regression results are shown in Figures S8c and S8d. Figure S8c highlights a strong alignment between predicted and ground truth line widths, while the residuals in Figure S8d are predominantly within a ± 0.2 Hz range. A slight bias is observed, where smaller line widths tend to be overestimated and larger ones underestimated.

Signal region width predictions are detailed in Figures S9a and S9b. Figure S9a illustrates that predicted signal region widths align closely with their ground truth values, reflecting the model's spatial localization accuracy. The residuals

in Figure S9b reveal that errors are mostly confined to a ± 3 Hz range, emphasizing the robustness of the predictions across various spin systems.

Coupling constant predictions are shown in Figures S9c and S9d. Figure S9c demonstrates a robust correlation between predicted and ground truth coupling constants, with most deviations falling within a ± 0.2 Hz range. The residuals in Figure S9d further highlight the model's consistency, with minimal discrepancies even in complex systems.

Figures S10a through S10b focus on precision-recall and threshold-dependent metrics. The precision-recall curve in Figure S10a demonstrates high precision across most recall values, with a minor drop at higher recalls, particularly in systems involving more protons (e.g., 3p, 4p). Figure S10b reveals that optimal F1 scores are achieved at a threshold of 0.4, while extreme thresholds lead to reduced performance.

The threshold-dependent average precision, visualized in Figure S10c, corroborates these findings, with the highest precision observed between thresholds of 0.4 and 0.6. Additionally, Figure S10d examines average precision across various Intersection over Union (IoU) thresholds. The model performs robustly under looser IoU criteria, but its performance diminishes at stricter thresholds, particularly beyond 0.6.

Lastly, the confusion matrix in Figure S10e highlights classification performance across multiple proton count scenarios, including the ”no spin” category. The matrix demonstrates the model's high accuracy in distinguishing different proton counts while revealing areas for improvement in more complex spin systems.

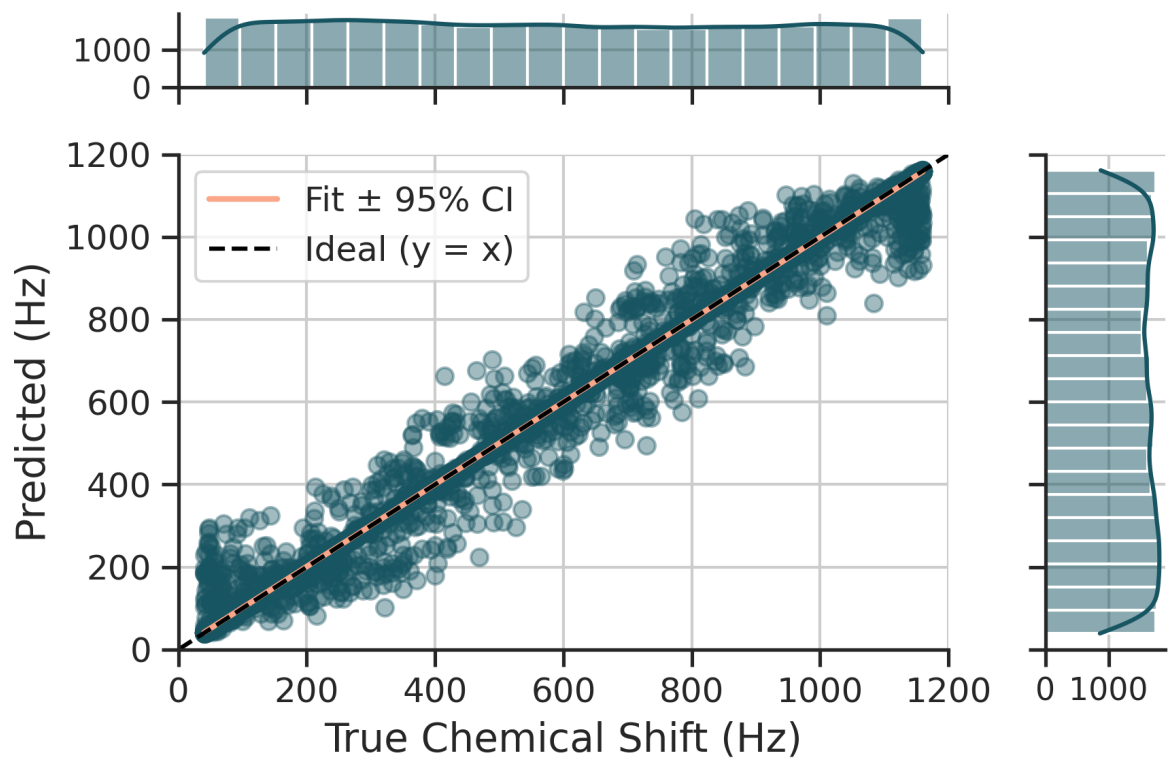

(a) Chemical shift predictions versus ground truth.

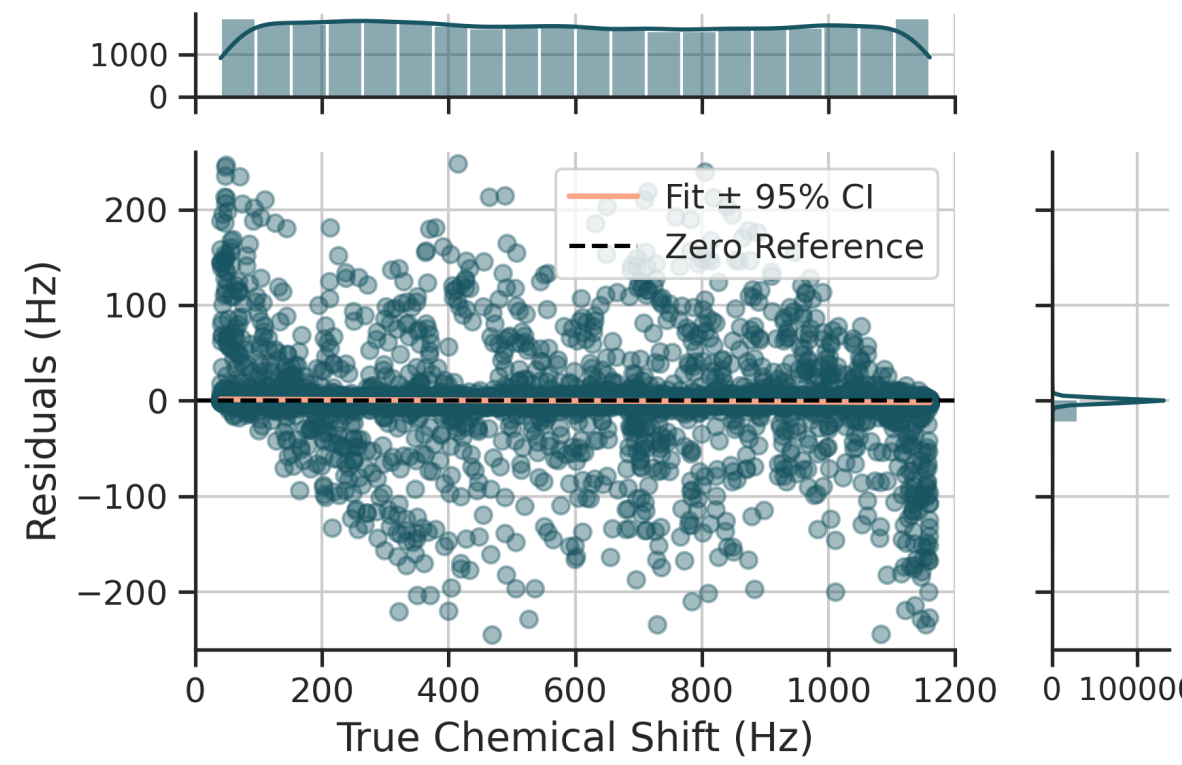

(b) Chemical shift residuals.

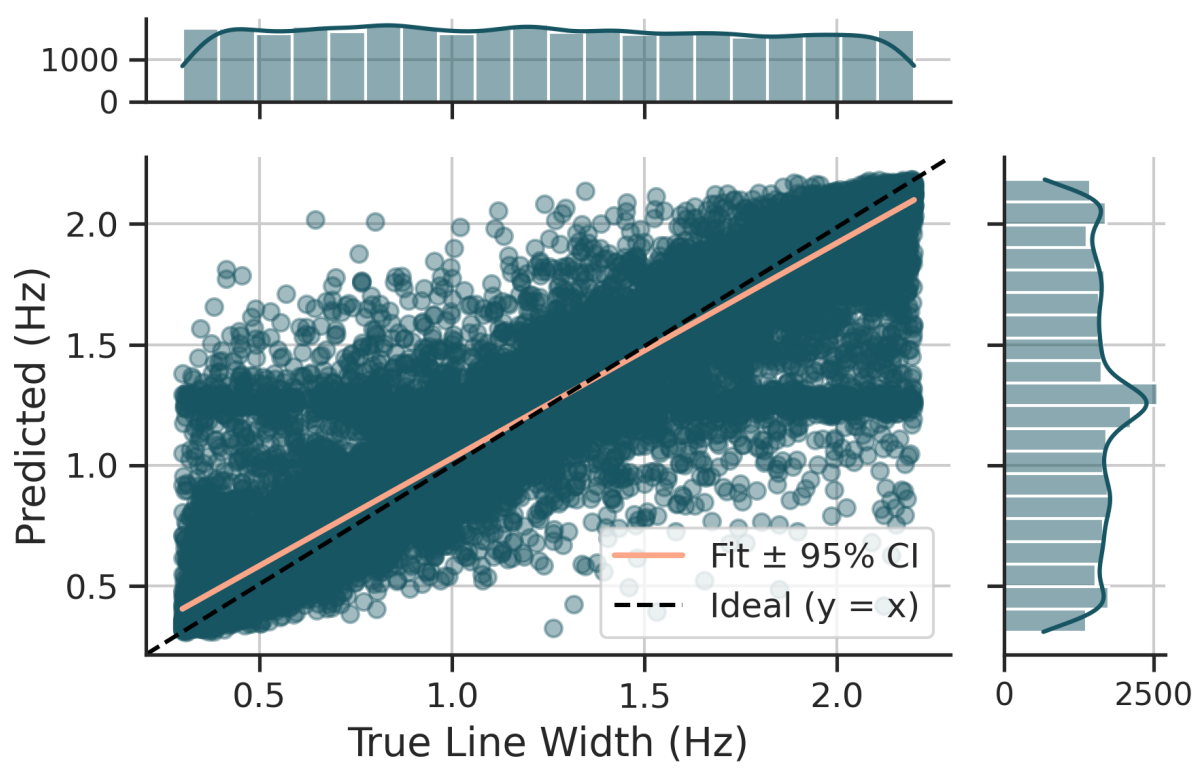

(c) Line width predictions versus ground truth.

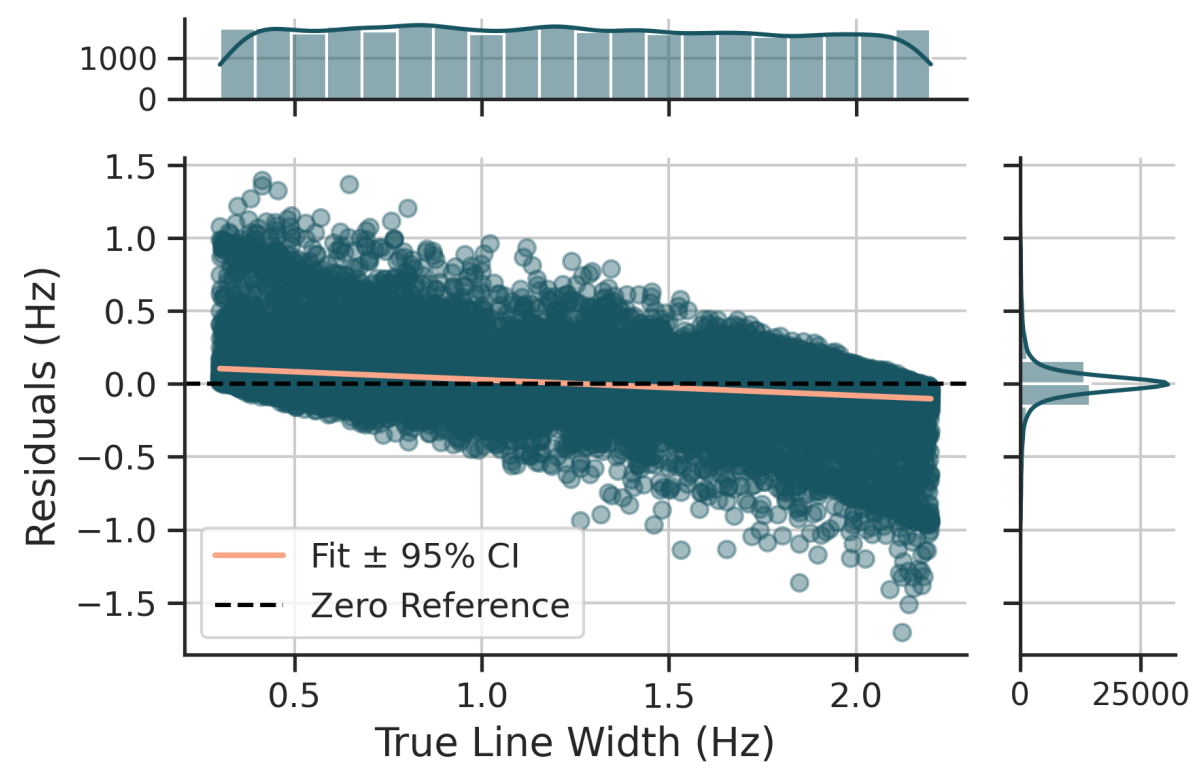

(d) Line width residuals.

Figure S8: Evaluation metrics for multiple independent spin systems (1). This figure compares predicted values with ground truth for key spectral parameters. (a) The scatter plot shows the relationship between predicted chemical shifts and ground truth values, highlighting the model's accuracy. (b) The residuals for chemical shift predictions indicate the consistency of the predictions, with deviations confined to a narrow range. (c) Predicted line width versus ground truth values, showing the model's ability to estimate this parameter accurately. (d) Residuals for line width predictions reflect the reliability and robustness of the model's performance.

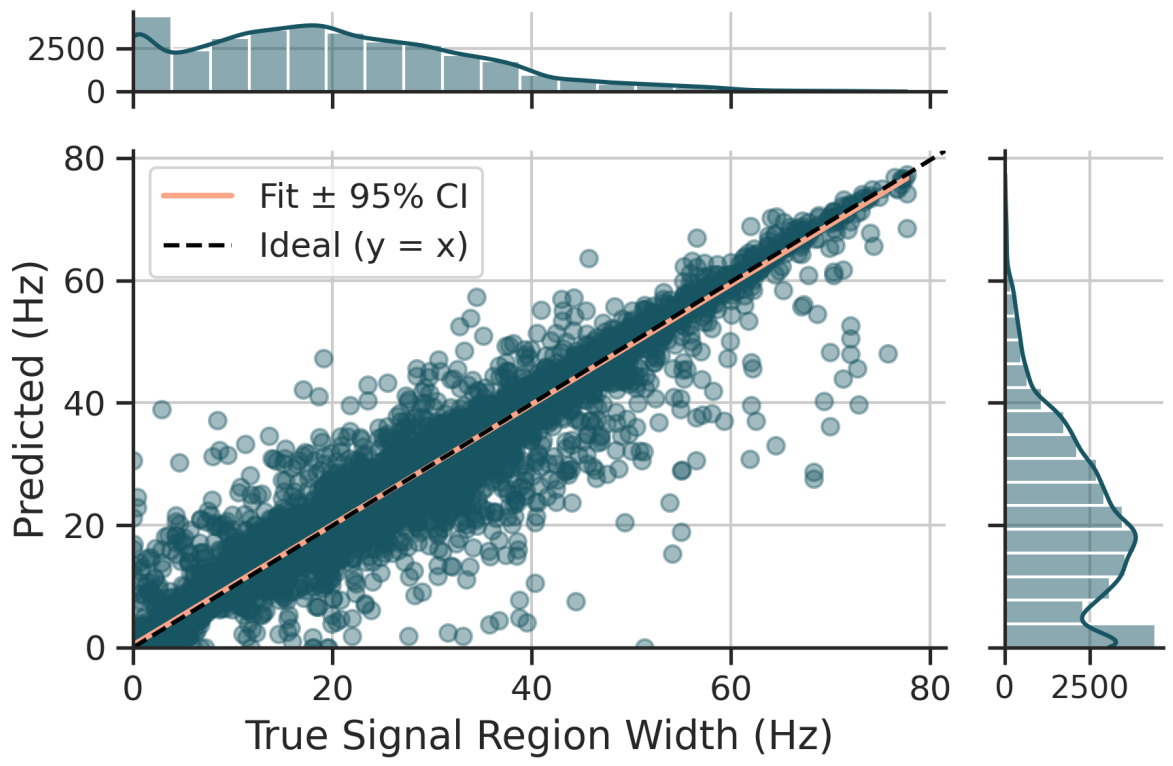

(a) Signal region width predictions versus ground truth.

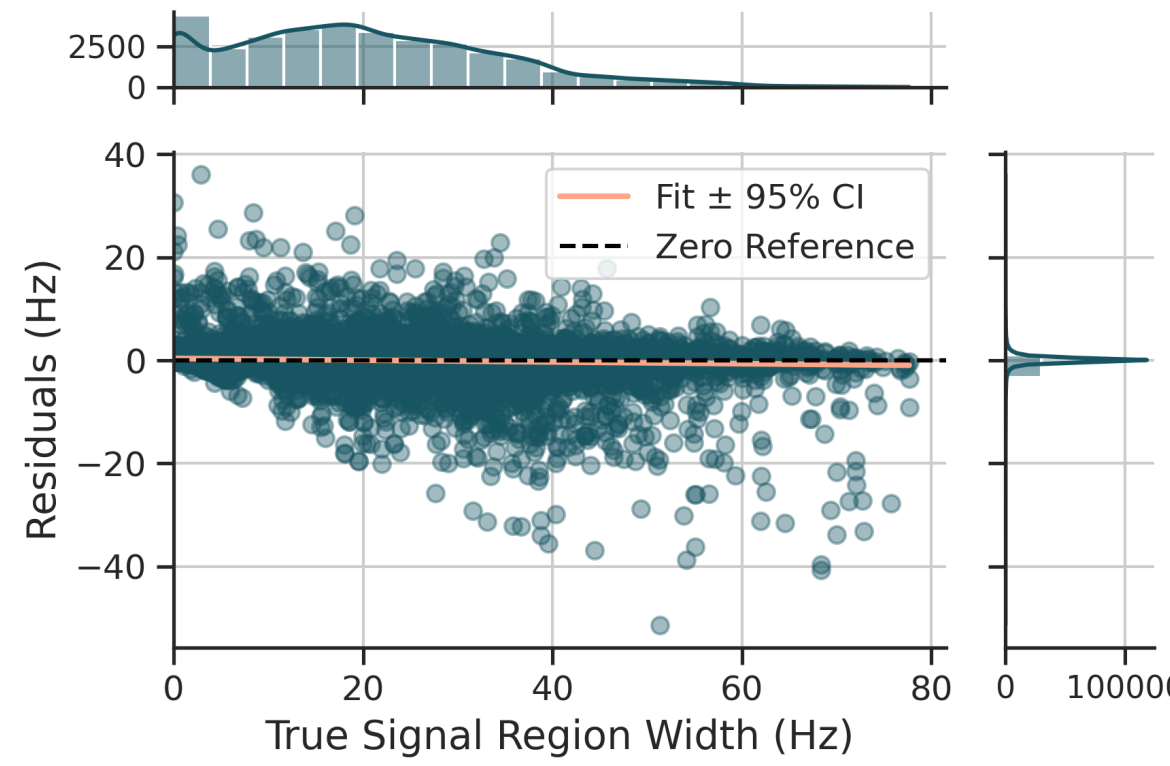

(b) Signal region width residuals.

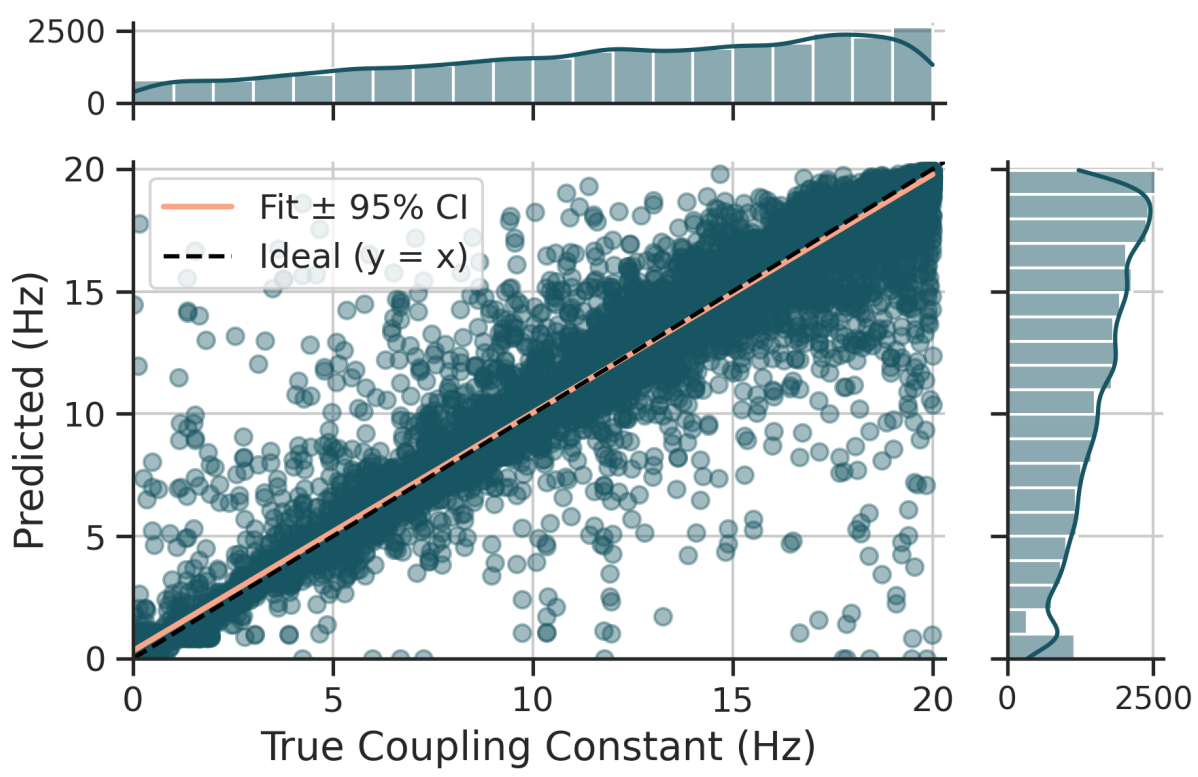

(c) Coupling constant predictions versus ground truth.

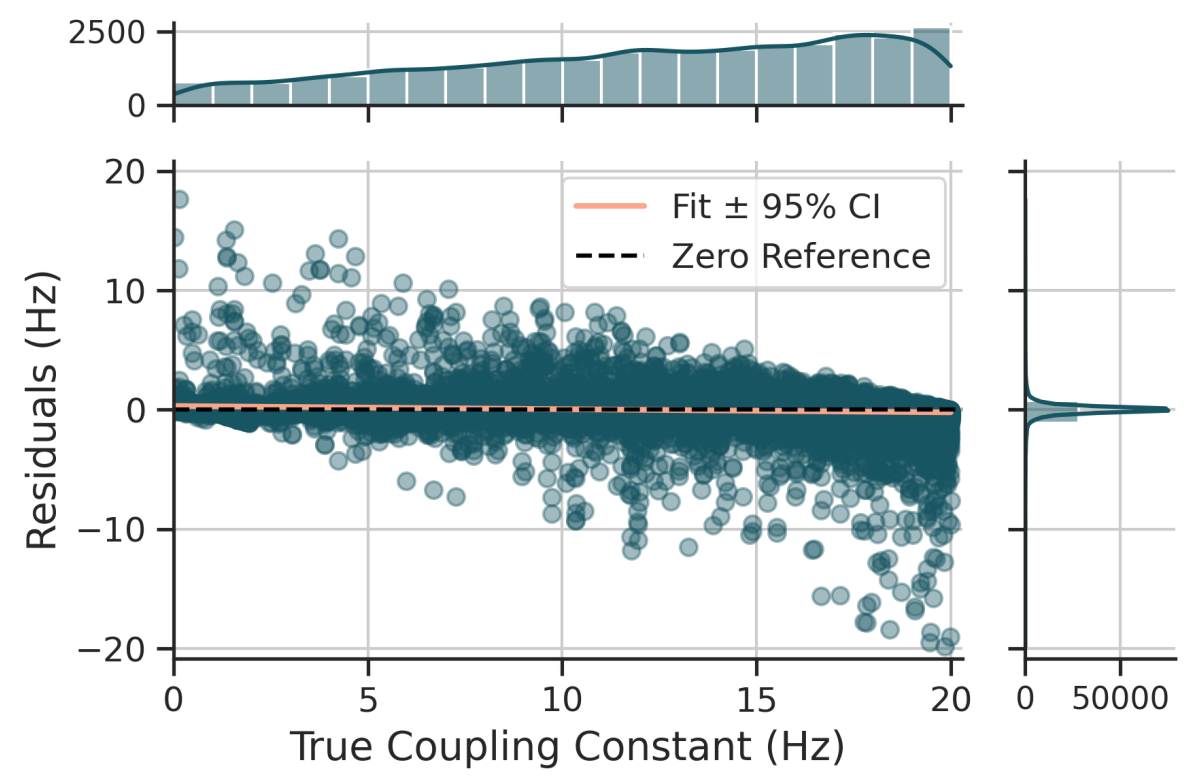

(d) Coupling constant residuals.

Figure S9: Evaluation metrics for multiple independent spin systems (2). This figure presents a detailed comparison of predicted values versus ground truth for coupling constants, signal region widths, and their residuals. (a) Predicted signal region versus ground truth values, showing spatial alignment accuracy. (b) Residuals for signal region width predictions, highlighting minor deviations. (c) Coupling constant predictions compared with ground truth values, demonstrating robust model performance. (d) Residuals for coupling constant predictions provide insights into prediction consistency and any discrepancies across spin systems.

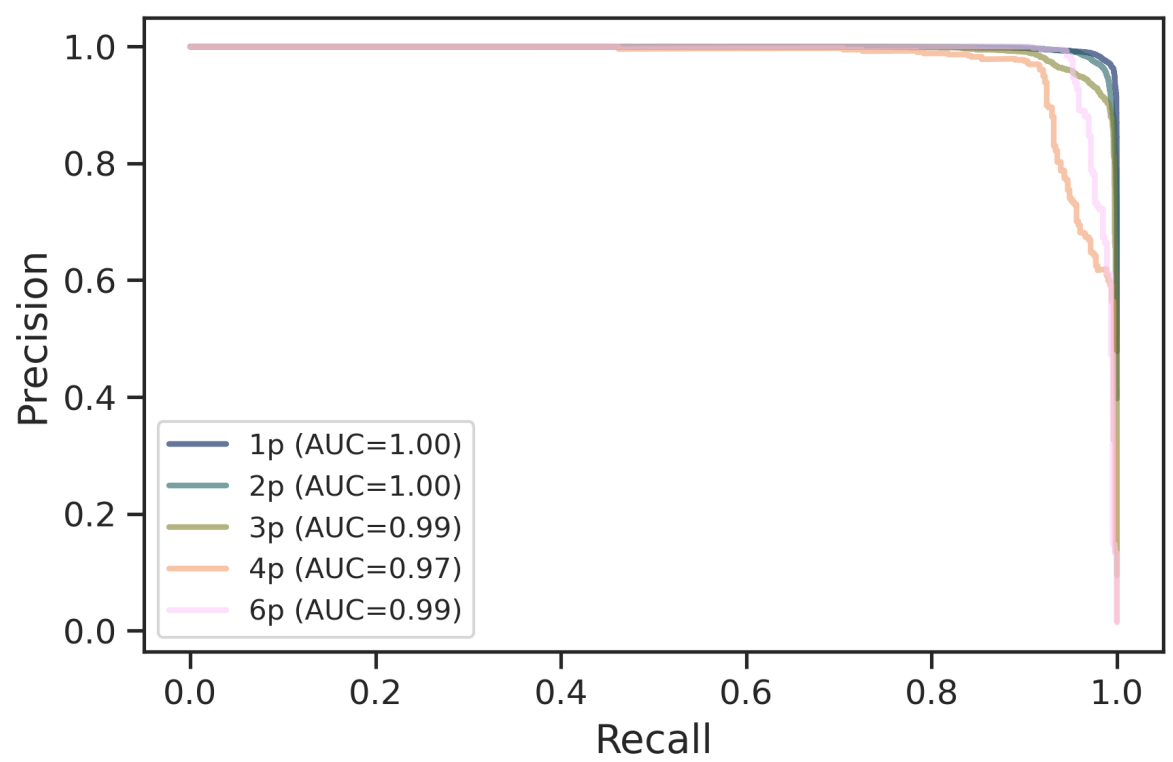

(a) Precision recall curve.

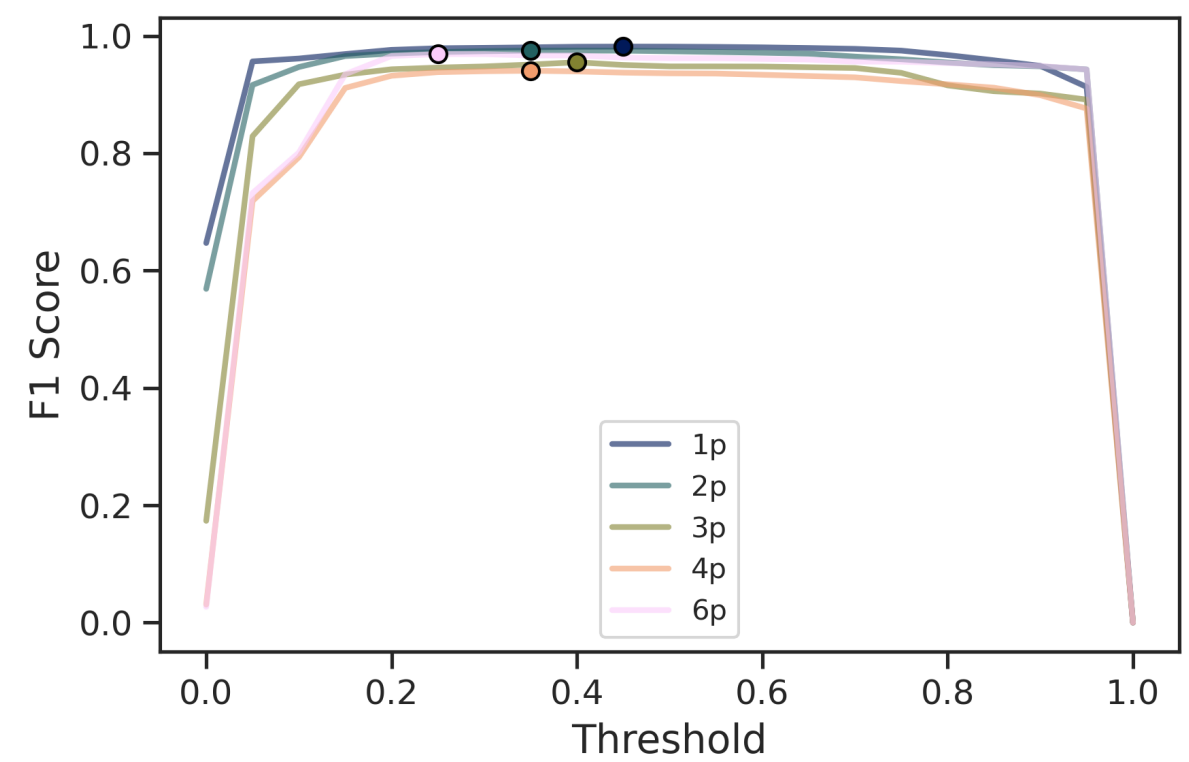

(b) F1 score versus threshold.

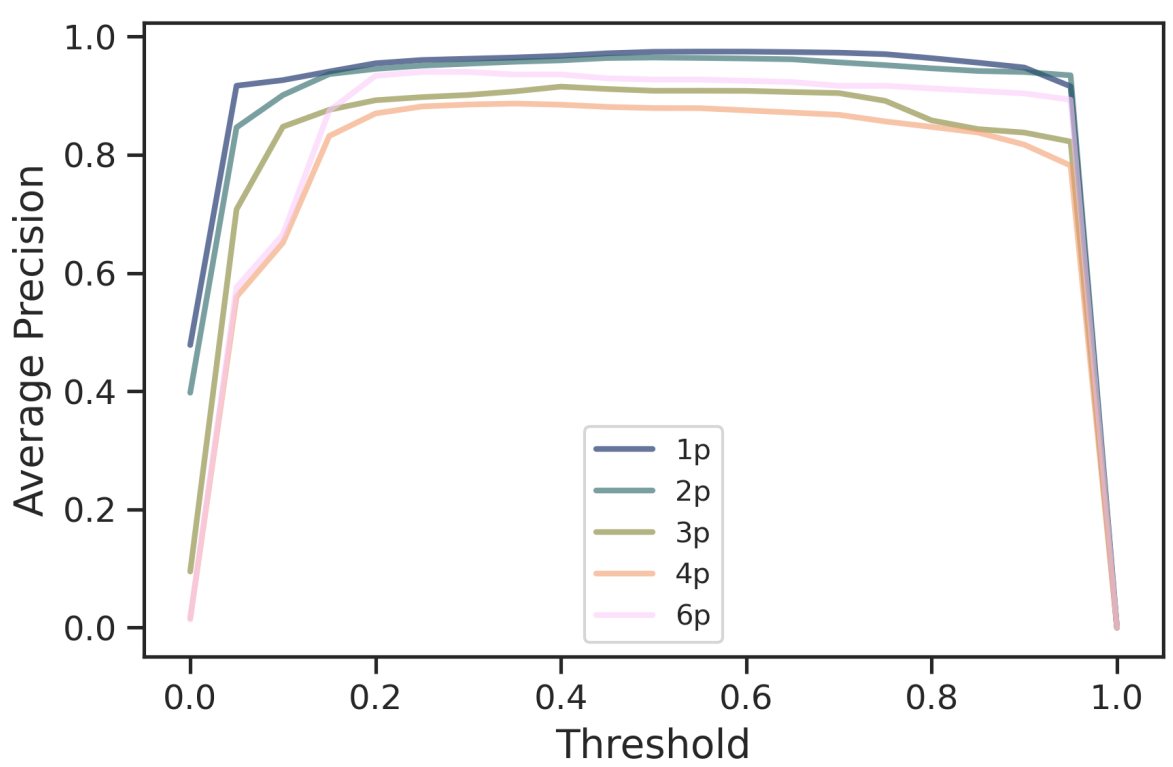

(c) Average precision versus threshold.

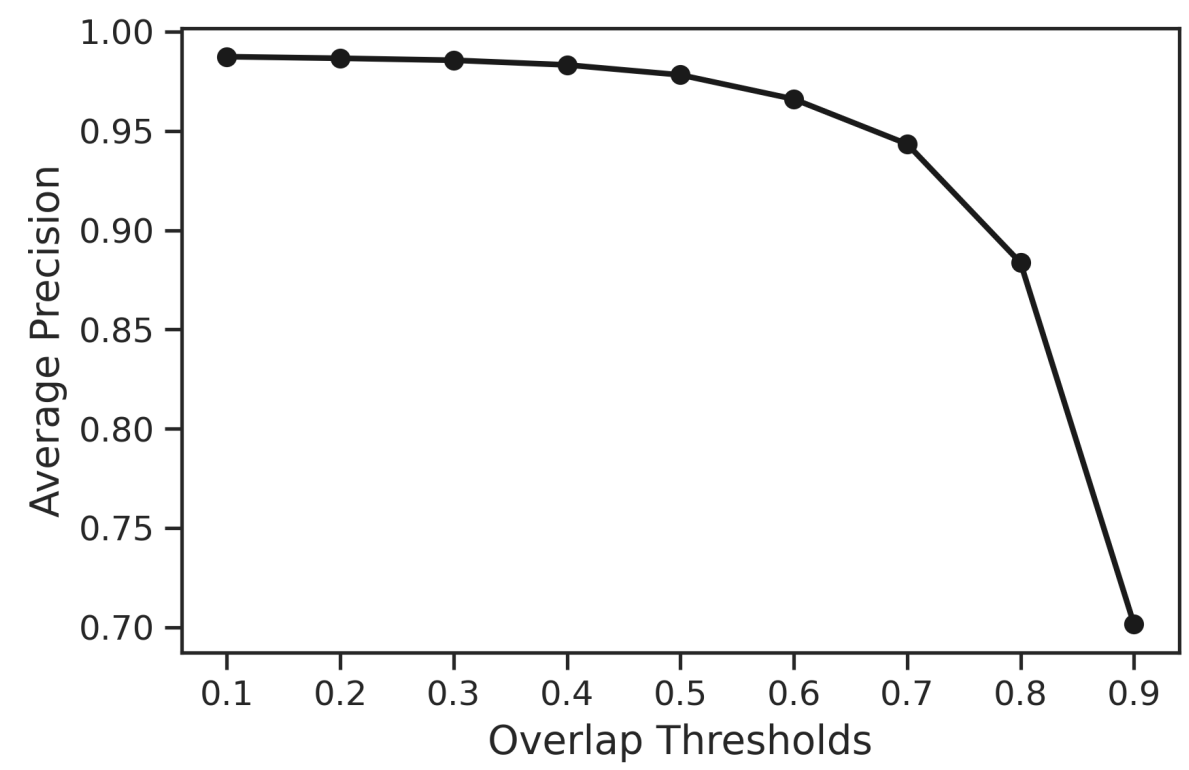

(d) Average precision for different IoU thresholds.

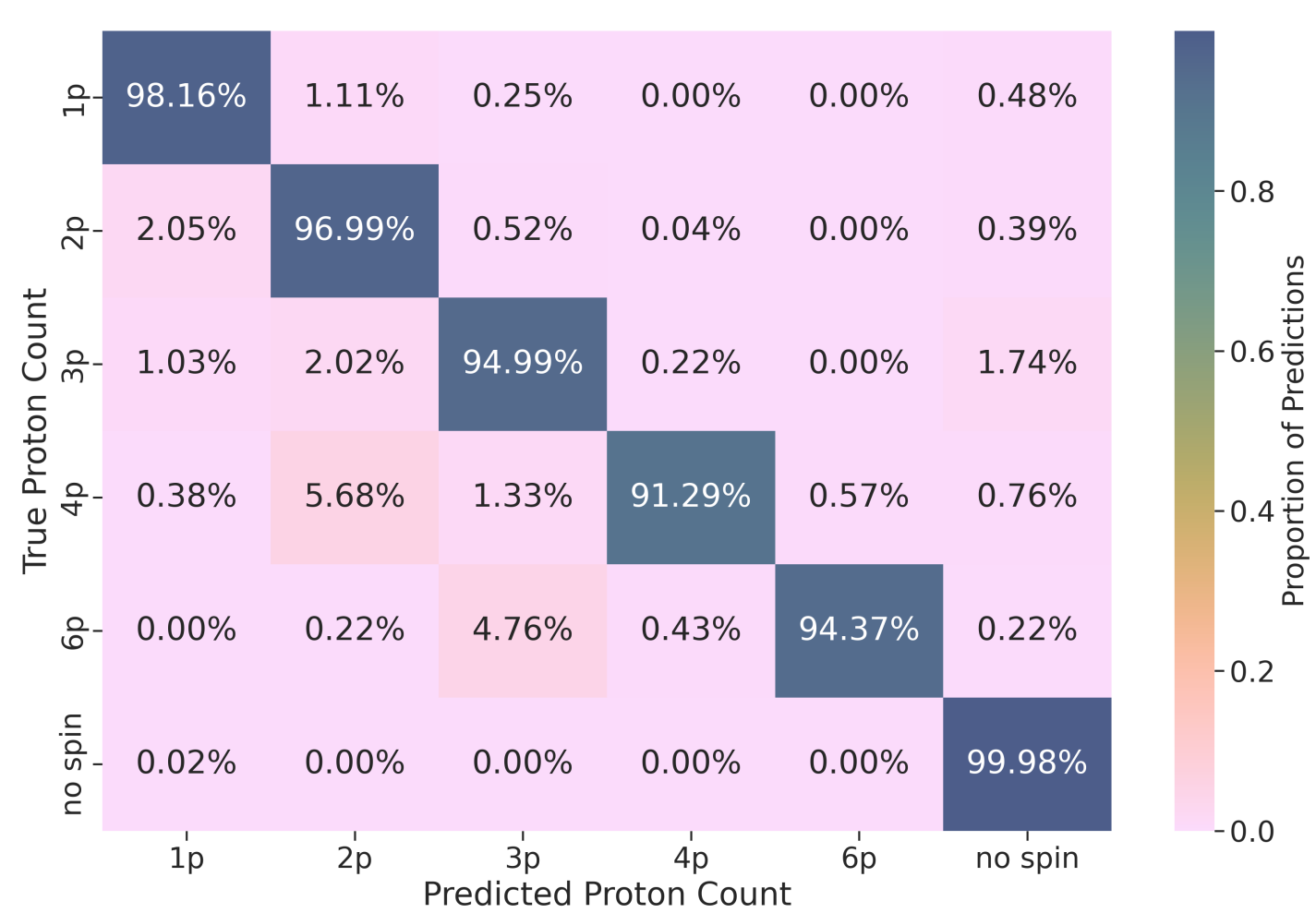

(e) Confusion matrix.

Figure S10: Evaluation metrics for multiple independent spin systems (3). This figure provides a performance analysis based on precision-recall and threshold-dependent metrics for spin identification and spatial signal alignment. (a) The precision-recall curve illustrates model performance in balancing sensitivity and specificity. (b) F1 score as a function of threshold shows the trade-off between precision and recall at various decision boundaries. (c) Average precision as a function of threshold provides insight into performance consistency across decision boundaries. (d) Average precision at different IoU thresholds demonstrates model robustness across spatial alignment criteria, with various IoU levels for accurate signal identification. (e) The confusion matrix highlights classification accuracy across proton counts, including the ”no spin” category, and identifies areas for improvement in complex systems.

4.2 Extended Results Weakly Coupled Spin Systems

Figures S11, S12, and S13 show further analysis of weakly coupled spin systems on distorted simulated data, extending the study presented in the Results Section. Spectra 1 and 3 from SI Section 3.2.1 serve as representative examples of these systems.

As presented in Table S5, the performance metrics for line widths and signal region widths in single weakly coupled spin systems exhibit strong correlations, with coefficients of determination (R^2) of 0.988 and 0.991, respectively. The low MAE and median absolute error values indicate high precision in the estimations, where line widths achieve an MAE of 0.034 Hz and a median error of 0.022 Hz, while signal region widths exhibit an MAE of 0.431 Hz and a median error of 0.163 Hz.

Table S5: Performance for single weakly coupled spin systems.

	**Line Widths**	**Signal Region Widths**
**Coefficient of Determination (R^2)**	0.988	0.991
**Mean Absolute Error (Hz)**	0.034	0.431
**Median Absolute Error (Hz)**	0.022	0.163

Figure S11 illustrates the regression results for chemical shifts and line widths in weakly coupled spin systems. In Figure S11a, the predicted chemical shifts show excellent agreement with the ground truth values, as evidenced by the data points closely aligning along the diagonal line. The residuals displayed in Figure S11b are predominantly distributed within a narrow range around zero, indicating high prediction accuracy with minimal systematic bias. Similarly, Figure S11c demonstrates that the predicted line widths closely match the true values, with Figure S11d showing residuals largely confined within a small interval around zero, suggesting precise and reliable estimations across different line width ranges.

A comparison of Tables S5 and S6 reveals that strongly coupled spin systems exhibit higher mean absolute errors (MAE) for line widths, increasing from 0.034 Hz to 0.066 Hz, while signal region widths show only minor declines. This decrease in line width accuracy may be attributed to the increased complexity and overlapping multiplets inherent in strongly coupled systems, which can obscure precise parameter extraction.

Figure S12 illustrates the model's performance in predicting both signal region widths and coupling constants. In Figure S12a, the predicted signal region widths exhibit a strong correlation with the ground truth, with most data points closely aligning along the identity line. This alignment signifies precise localization of signal boundaries. The residuals in Figure S12b are predominantly centered around zero, indicating minimal prediction errors, although some deviations occur for very narrow signal region widths. For coupling constants, Figure S12c demonstrates a robust correspondence between predicted and true values, though a slight dispersion is evident for small coupling constants. This dispersion is expected, as distinguishing multiplets with very small coupling constants from each other or from singlets becomes increasingly challenging. For instance, two single peaks with broader tips could result either from

small coupling constants (two coupled spins, i.e., two doublets) or from field inhomogeneities (two uncoupled spins, i.e., two singlets). Since the determination of signal region widths depends on the coupling constants, a similar explanation applies here. Interestingly, the performance for multiple independent spin systems is even better in this context (see Section 4.1). We hypothesize that the presence of multiple spin systems facilitates the inference of proton counts and coupling constants in these ambiguous cases and aids in separating signals from distortions caused by factors such as field inhomogeneities. The residuals depicted in Figure S12d are generally small, though they exhibit a minor spread.

Figure S13 summarizes various classification performance metrics for weakly coupled spin systems across different proton counts (1p, 2p, 3p). The precision-recall curves in Figure S13a exhibit high precision across a wide range of recall values for all proton systems, indicating robust and consistent classification performance. The F1 score versus threshold plot in Figure S13b reveals optimal performance around a threshold value of 0.4, where the balance between precision and recall is maximized. Figure S13c confirms this optimal threshold range by showing peak average precision between thresholds of 0.4 and 0.6. The average precision for different IoU thresholds in Figure S13d indicates strong model performance at lower IoU thresholds, with a gradual decline as the IoU threshold increases, suggesting decreased accuracy under stricter overlap criteria. Lastly, the confusion matrix in Figure S13e demonstrates high overall classification accuracy, with most instances correctly classified across all proton counts and only a few misclassifications observed in the less frequent scenarios involving higher proton counts. Note that coupled spin systems in our dataset do not include 4p and 6p signals.

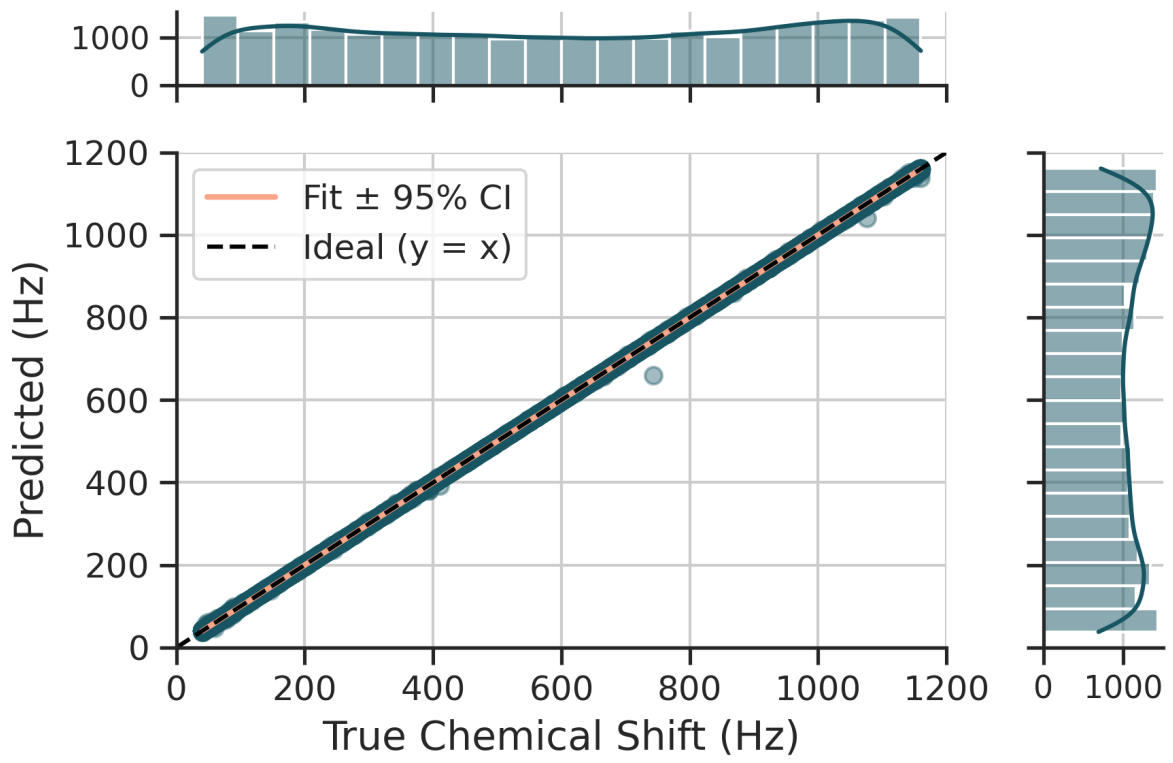

(a) Chemical shift predicted versus ground truth

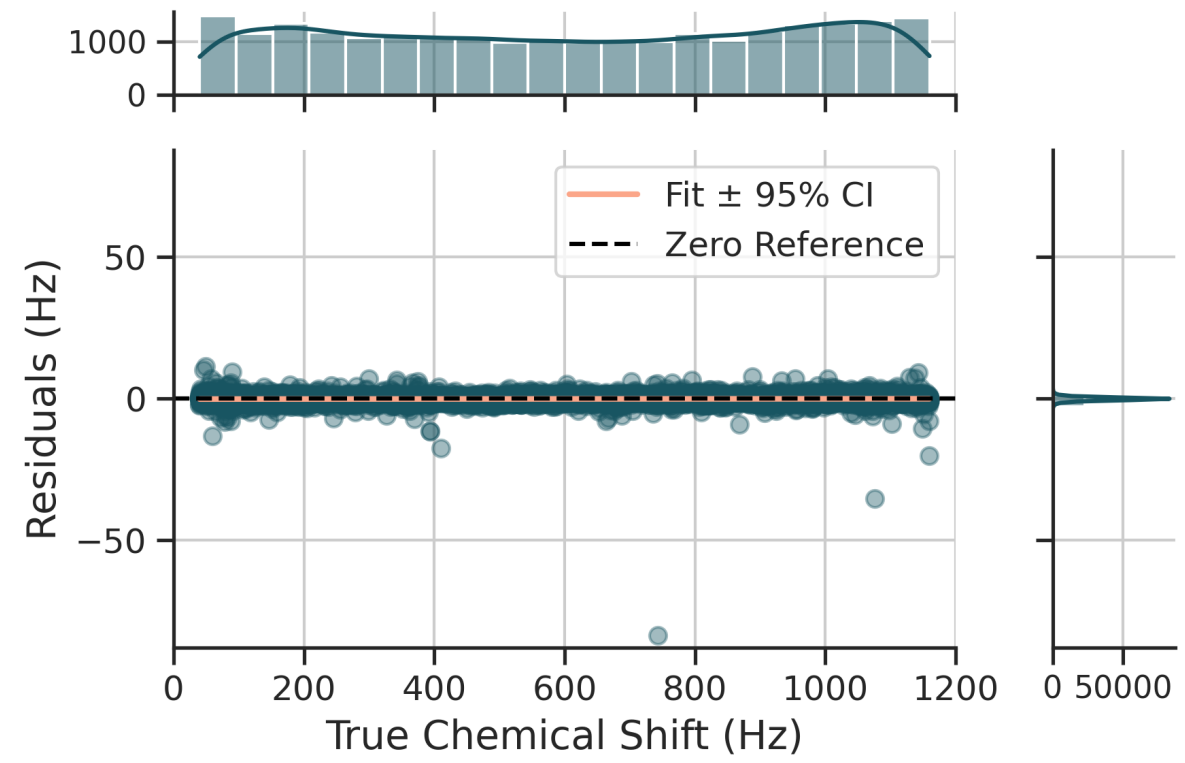

(b) Chemical shift residuals

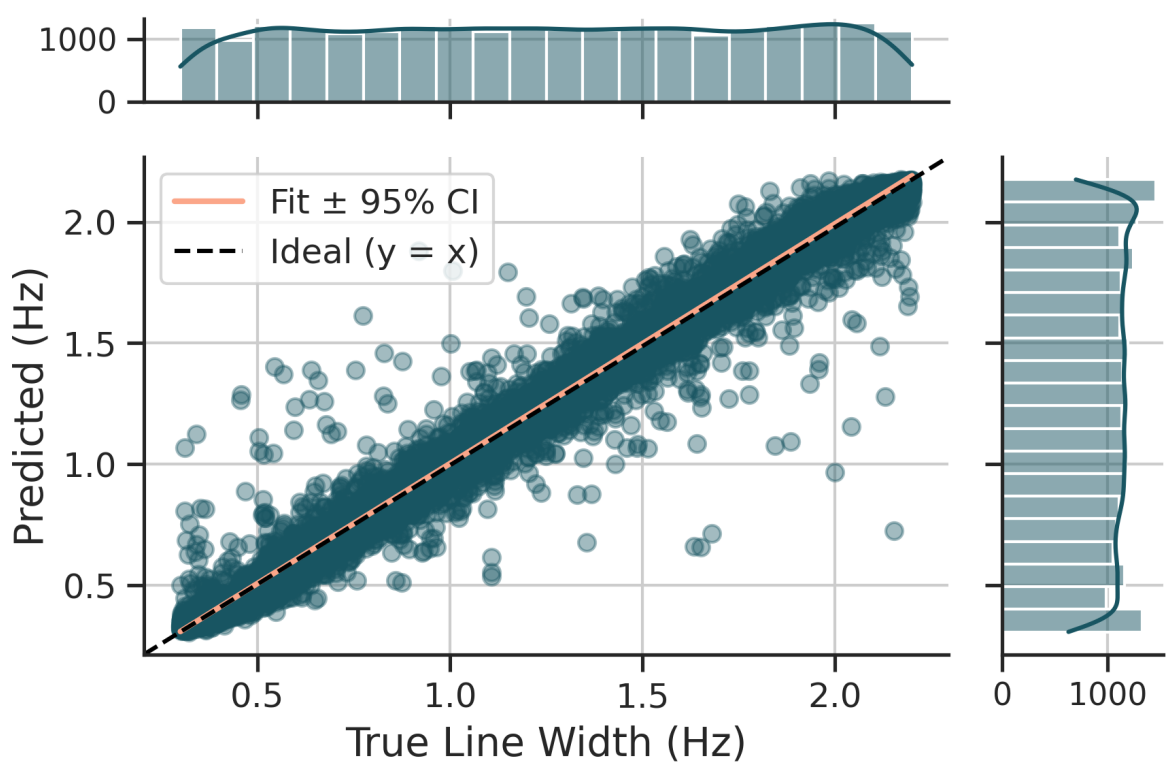

(c) Line width predicted versus ground truth

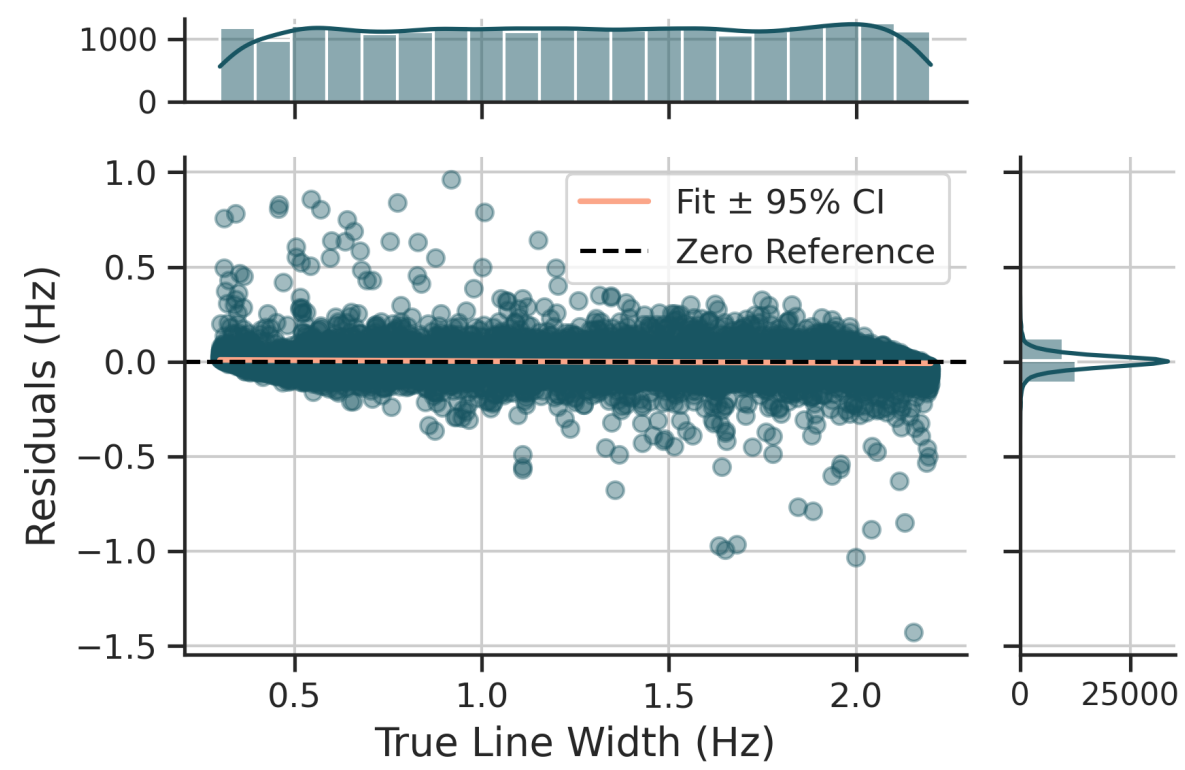

(d) Line width residuals

Figure S11: Evaluation metrics for weakly coupling spin systems (1): This figure compares predicted values with ground truth for key spectral parameters. (a) The scatter plot illustrates the relationship between the predicted chemical shifts and their corresponding ground truth values, showcasing the accuracy of the model. (b) The residuals for chemical shift predictions highlight the deviations between predicted and actual values, providing insight into model performance. (c) A similar scatter plot for line width shows the model's efficacy in estimating this parameter. (d) Line width residuals indicate the consistency and reliability of the predictions across the dataset.

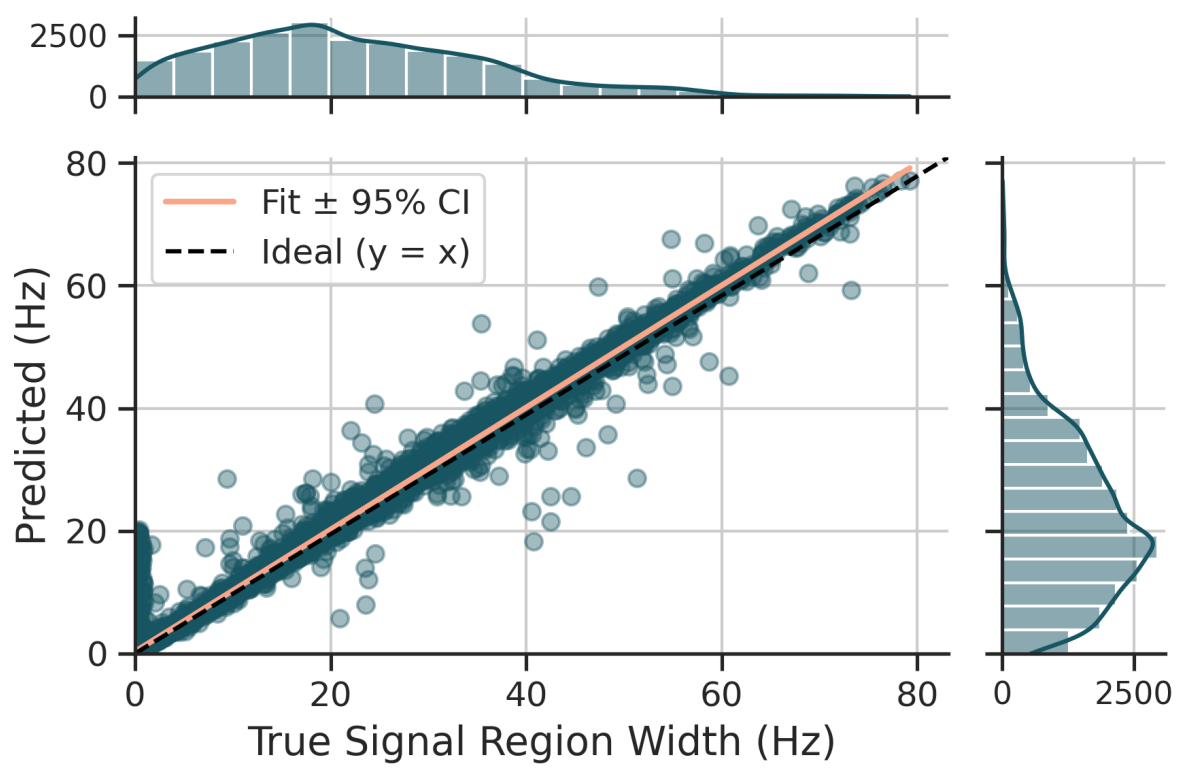

(a) Signal region predicted versus ground truth

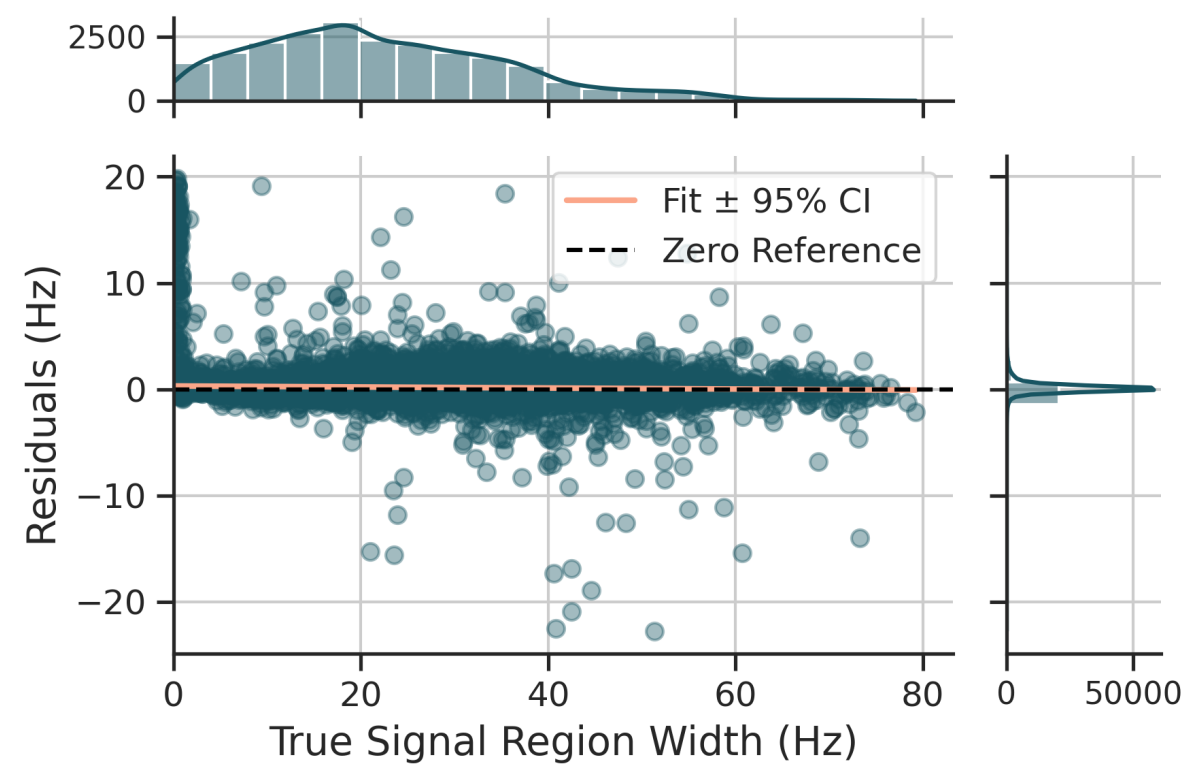

(b) Signal region residuals

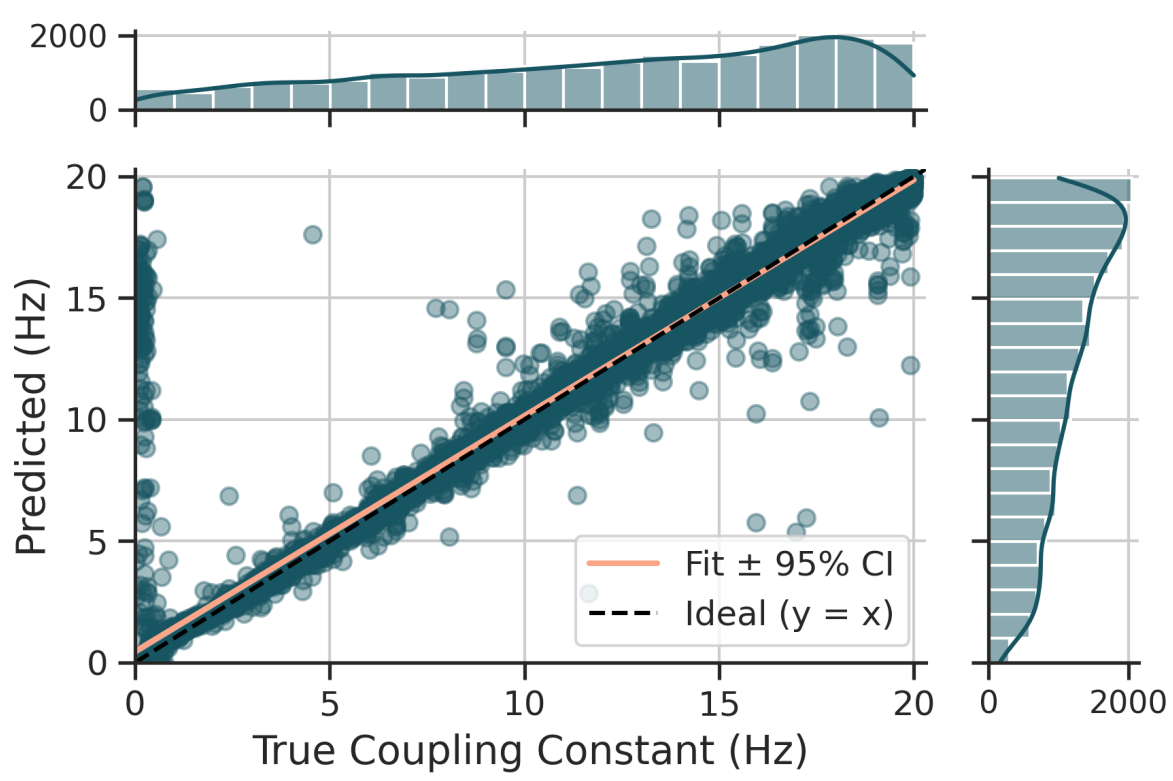

(c) Coupling constant predicted versus ground truth

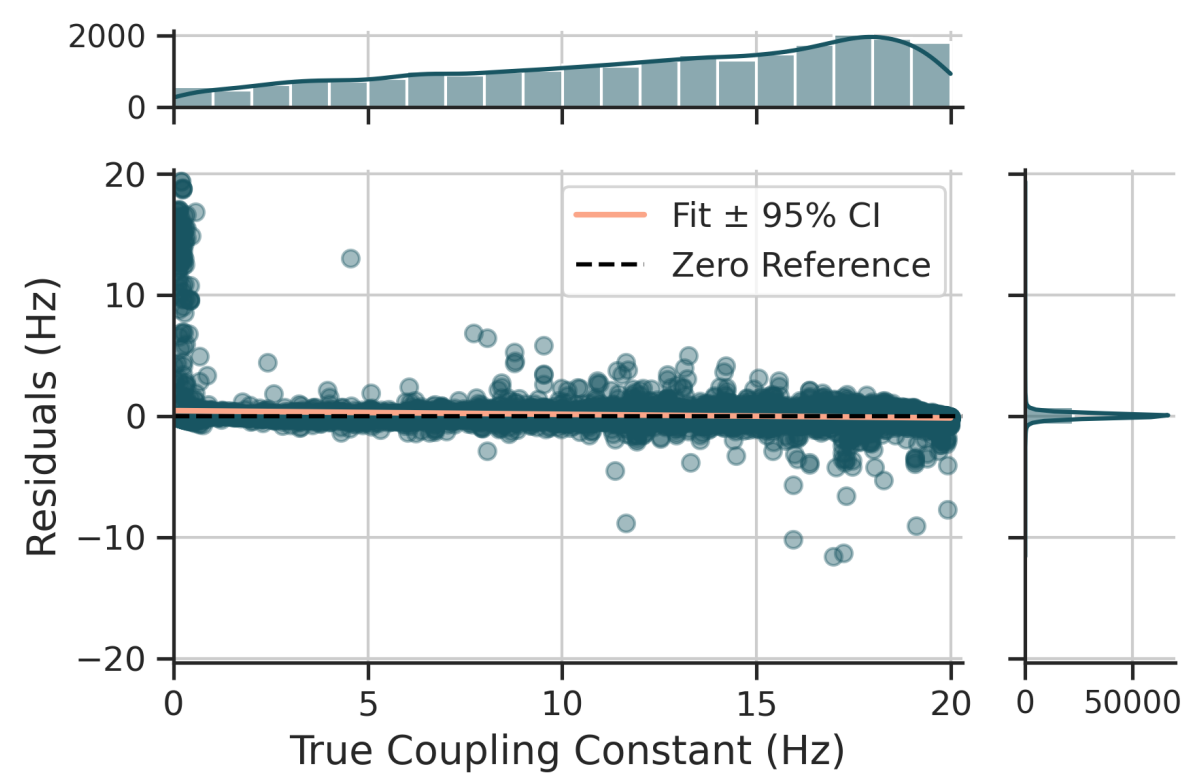

(d) Coupling constant residuals

Figure S12: Evaluation metrics for weakly coupling spin systems (2): This figure further analyzes model performance through signal region and coupling constant predictions. (a) The plot compares predicted signal region widths to ground truth, illustrating how well the model identifies spatial features in the spectra. (b) Residuals for signal region predictions reveal discrepancies, aiding in the assessment of spatial prediction accuracy. (c) The scatter plot for coupling constant predictions versus ground truth evaluates the model's performance in estimating interaction strengths. (d) Coupling constant residuals provide a detailed error analysis.

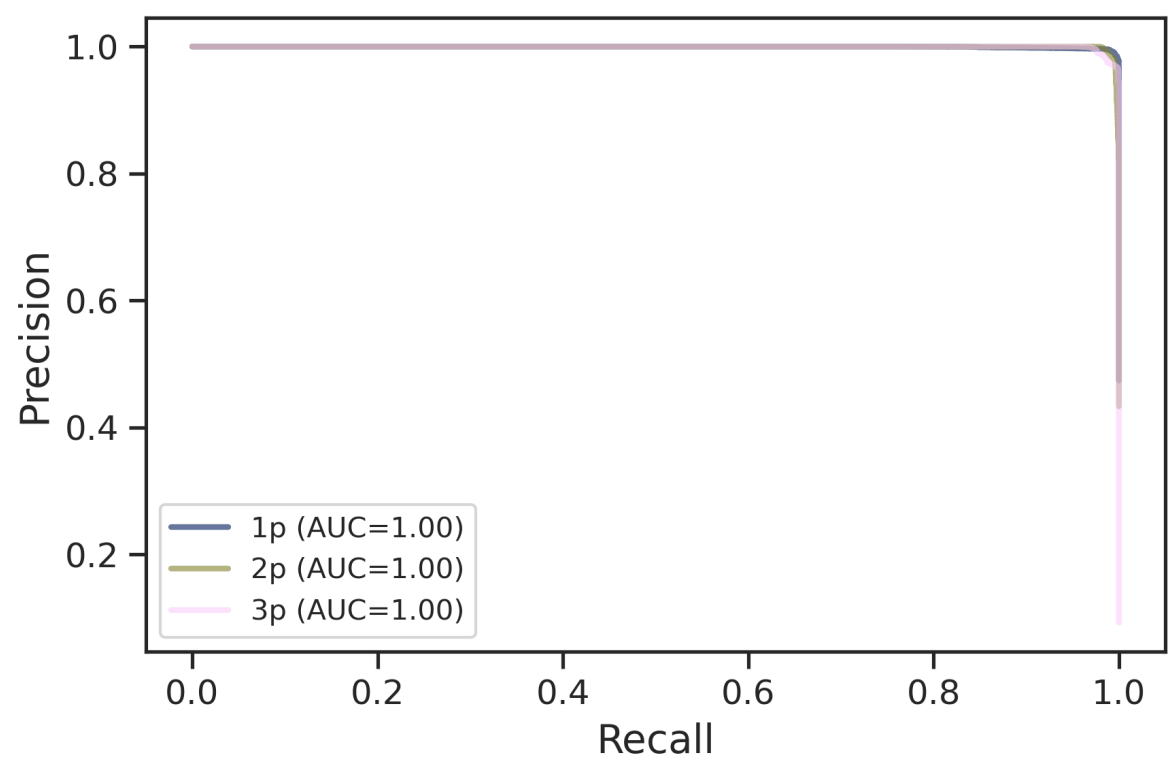

(a) Precision recall curve

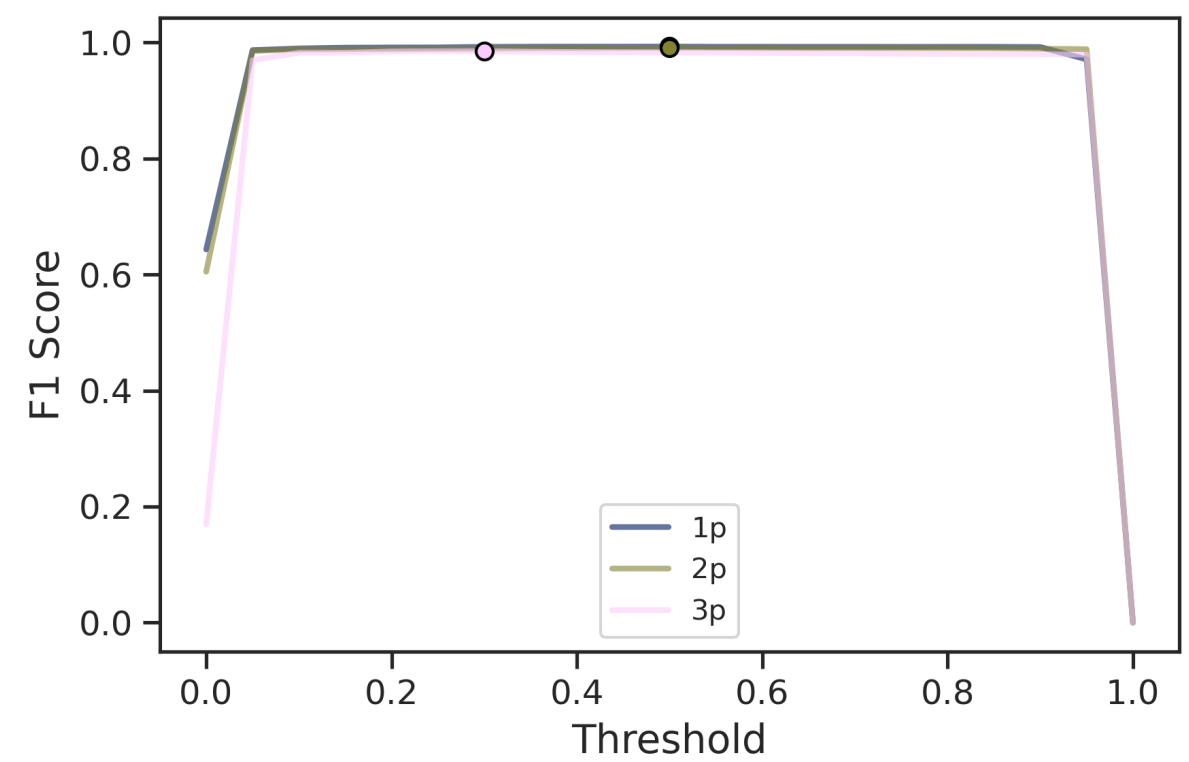

(b) F1 score versus threshold

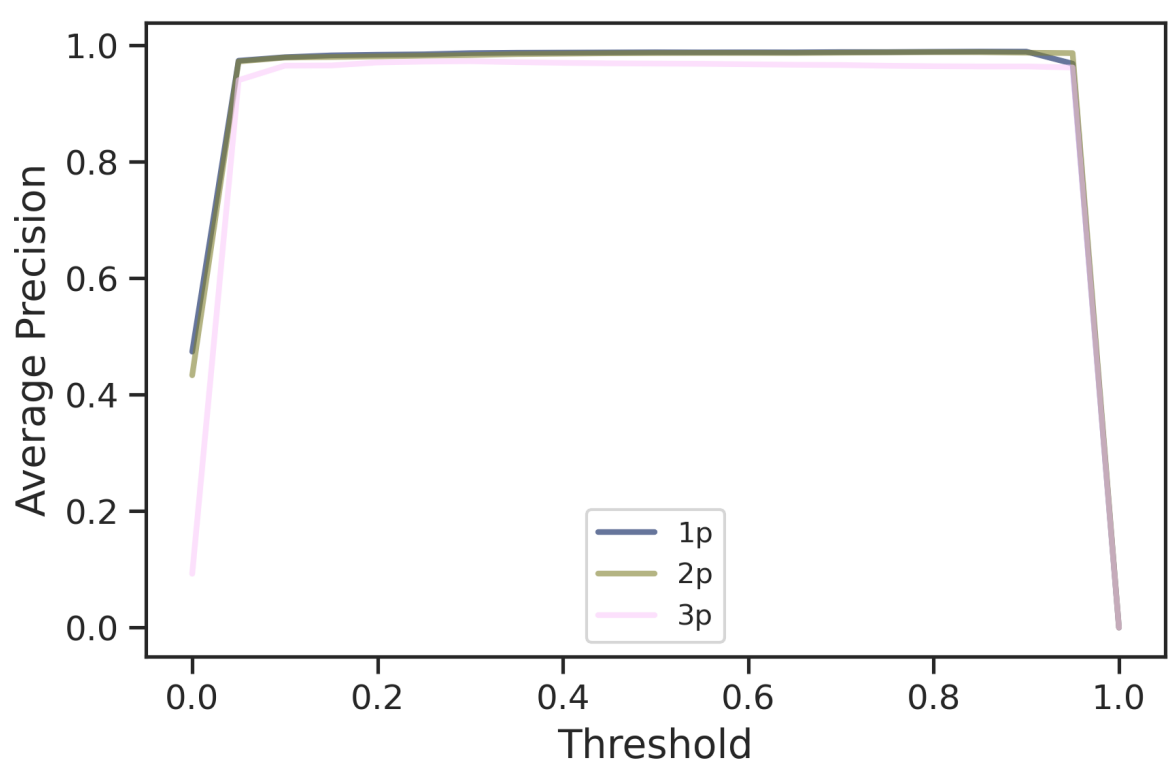

(c) Average precision versus threshold

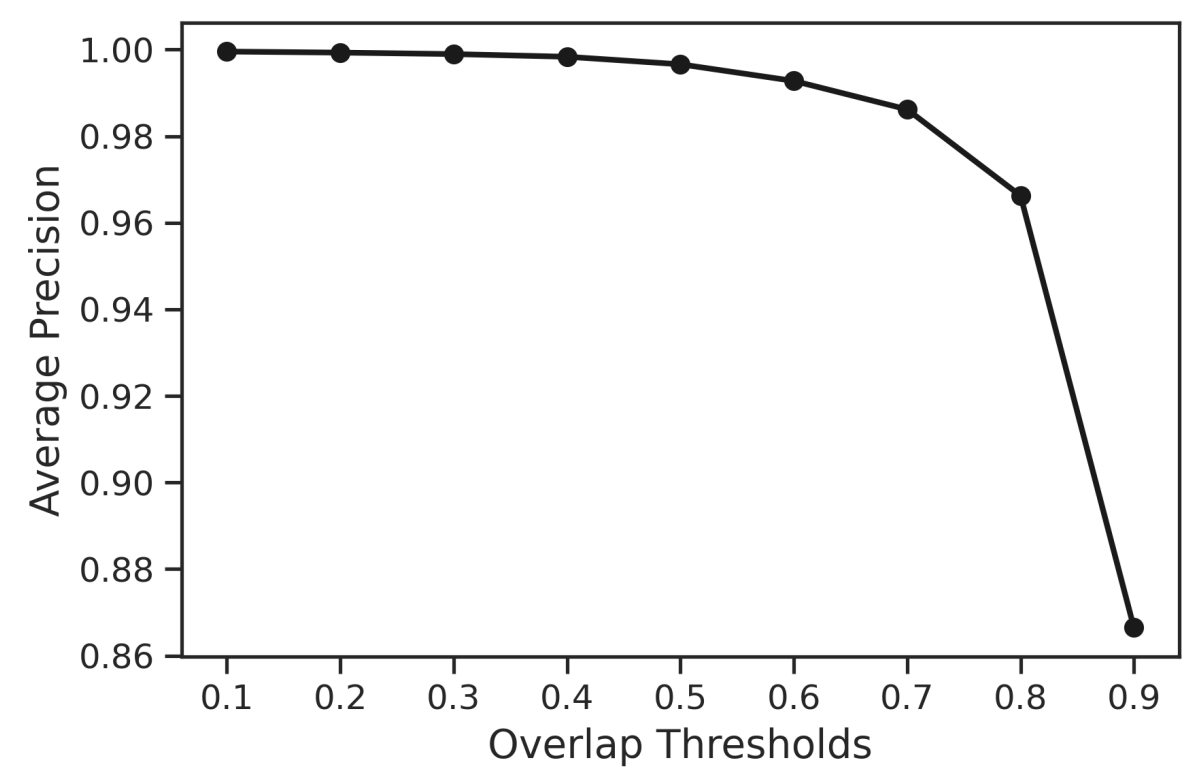

(d) Average precision for different IoU thresholds

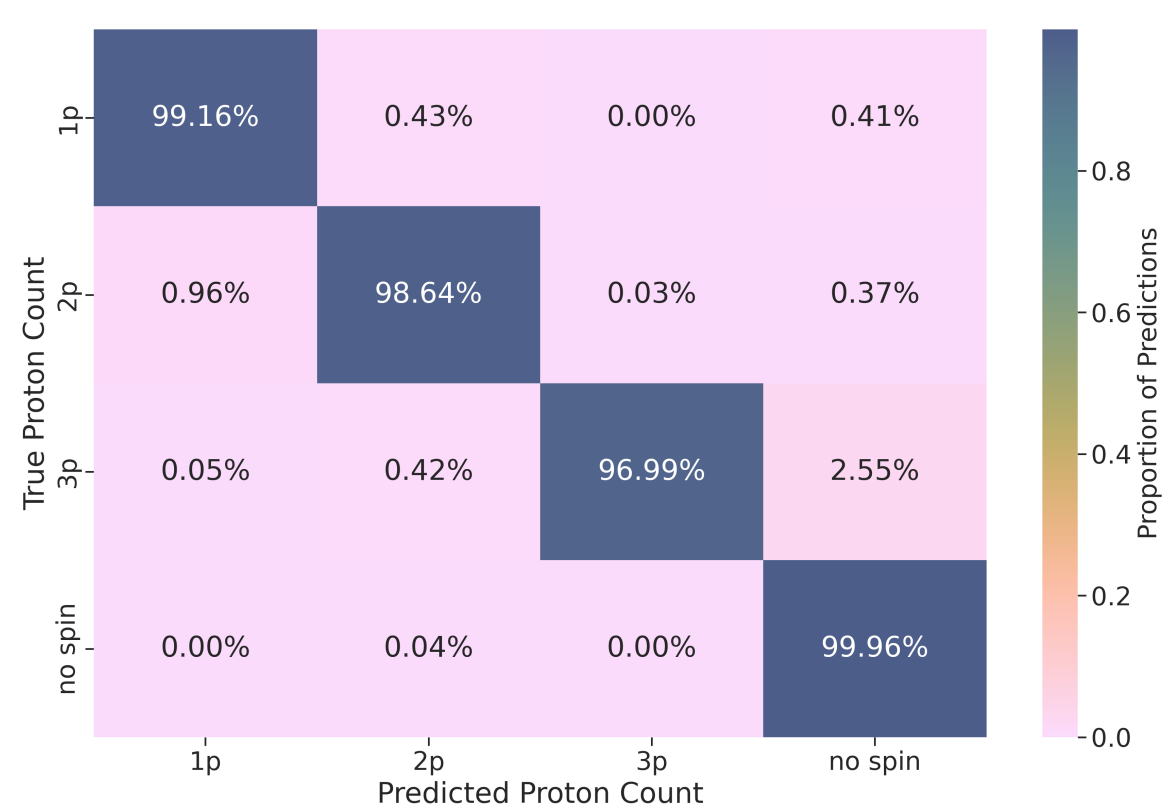

(e) Confusion matrix

Figure S13: Evaluation metrics for weakly coupling spin systems (3): This figure presents various performance metrics for the model. (a) The precision-recall curve illustrates the model's ability to distinguish between positive and negative instances for spin identification. (b) The F1 score plotted against threshold values demonstrates the balance between precision and recall across different decision boundaries. (c) Average precision as a function of threshold offers insights into the consistency of the model's performance. (d) Average precision across different IoU thresholds assesses model robustness in spatial alignment tasks. (e) The confusion matrix provides a comprehensive overview of model predictions, highlighting true positives, false positives, true negatives, and false negatives.

4.3 Extended Results for Strongly Coupled Spin Systems

Further analysis of strongly coupled spin systems on simulated data, building on the Results Section, is shown in Figures S14, S15, and S16. Spectra 4 and 5 from SI Section 3.2.1 serve as representative examples of these systems.

As presented in Table S6, the performance metrics for line widths and signal region widths in single strongly coupled spin systems exhibit strong correlations, with coefficients of determination (R^2) of 0.949 and 0.984, respectively. The low MAE and median absolute error values indicate high precision in the estimations, where line widths achieve an MAE of 0.066 Hz and a median error of 0.035 Hz, while signal region widths exhibit an MAE of 0.686 Hz and a median error of 0.258 Hz.

Table S6: Performance for strongly coupled spin systems.

	**Line Widths**	**Signal Region Widths**
**Coefficient of Determination (R^2)**	0.949	0.984
**Mean Absolute Error (Hz)**	0.066	0.686
**Median Absolute Error (Hz)**	0.035	0.258

Figure S14 presents the regression results for chemical shifts and line widths in strongly coupled spin systems. In Figure S14a, the predicted chemical shifts align well with the ground truth, as the points clustering around the diagonal line indicate. The residuals in Figure S14b reveal no bias in the predictions. Similarly, Figure S14c shows that line width predictions are generally accurate, although the residuals in Figure S14d exhibit a trend where lower line widths are slightly overestimated, and higher line widths are underestimated, suggesting some room for improvement.

Figure S15 evaluates the model's performance in predicting signal region widths and coupling constants within strongly coupled systems. In Figure S15a, the predicted signal region widths closely correspond to the ground truth, as indicated by the majority of data points aligning near the identity line. The residuals presented in Figure S15b are predominantly centered around zero, though some dispersion is evident. This suggests that while the predictions are generally reliable, their accuracy diminishes for narrow signal region widths. Regarding coupling constants, Figure S15c demonstrates a reasonable alignment between predicted and true values. However, the residuals shown in Figure S15d reveal a tendency for the model to slightly underestimate larger coupling constants. This underestimation may require further refinement to achieve more precise predictions. Notably, the ambiguity associated with small coupling constants (as observed in Section 4.2) appears to be less pronounced compared to weakly coupled systems. We hypothesize that this improvement may result from the more substantial alteration of signal profiles caused by strong coupling, even at small coupling constants, leading to more distinguishable signals.

Figure S16 summarizes the classification performance metrics for strongly coupled spin systems across different proton counts (1p, 2p, 3p). The precision-recall curves in Figure S16a demonstrate high precision across various recall levels, indicating consistent classification performance across different proton systems. The F1 score versus threshold plot in Figure S16b highlights an optimal threshold around 0.5, where the balance between precision and

recall is maximized. Figure S16c supports this finding, showing peak average precision within the 0.4 to 0.6 threshold range. The average precision for different IoU thresholds in Figure S16d shows that while the model performs well under less strict criteria, there is a noticeable decline in accuracy as the IoU threshold increases. Finally, the confusion matrix in Figure S16e shows high overall classification accuracy, with most instances correctly classified across all proton systems. However, minor misclassifications are observed, particularly in rare 3p systems. Note that coupled spin systems in our dataset do not include 4p and 6p signals.

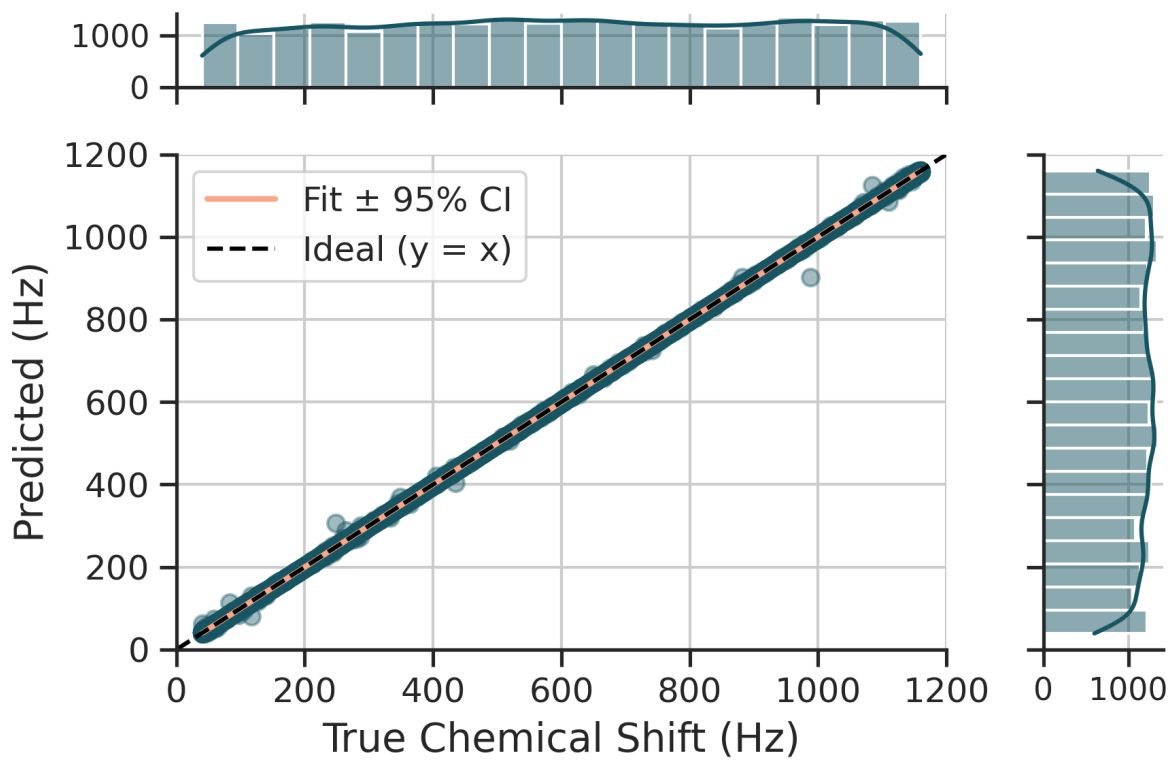

(a) Chemical shift predicted versus ground truth

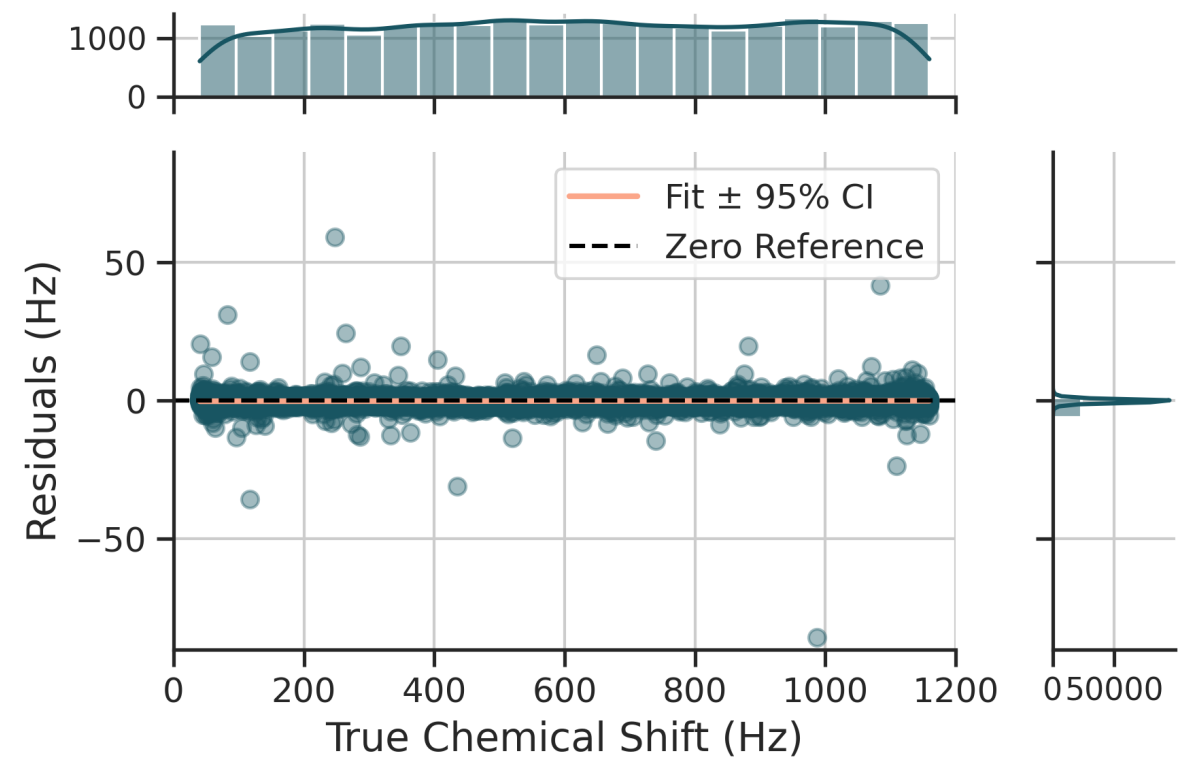

(b) Chemical shift residuals

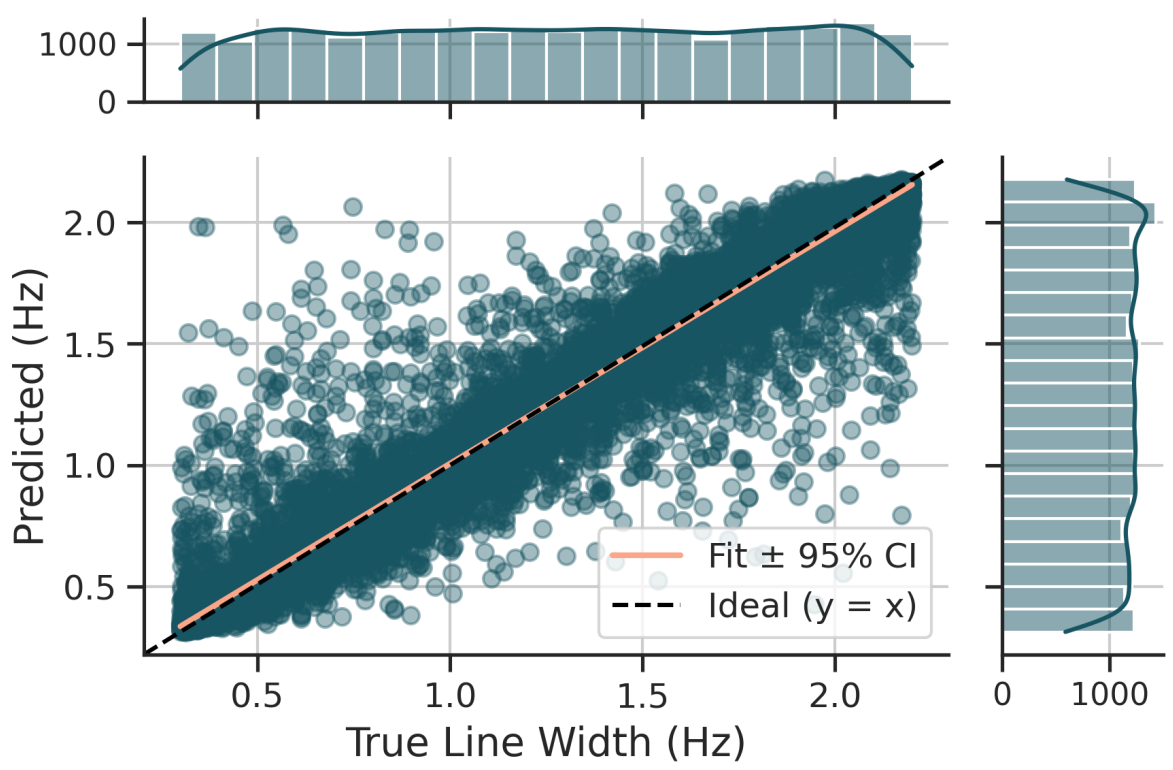

(c) Line width predicted versus ground truth

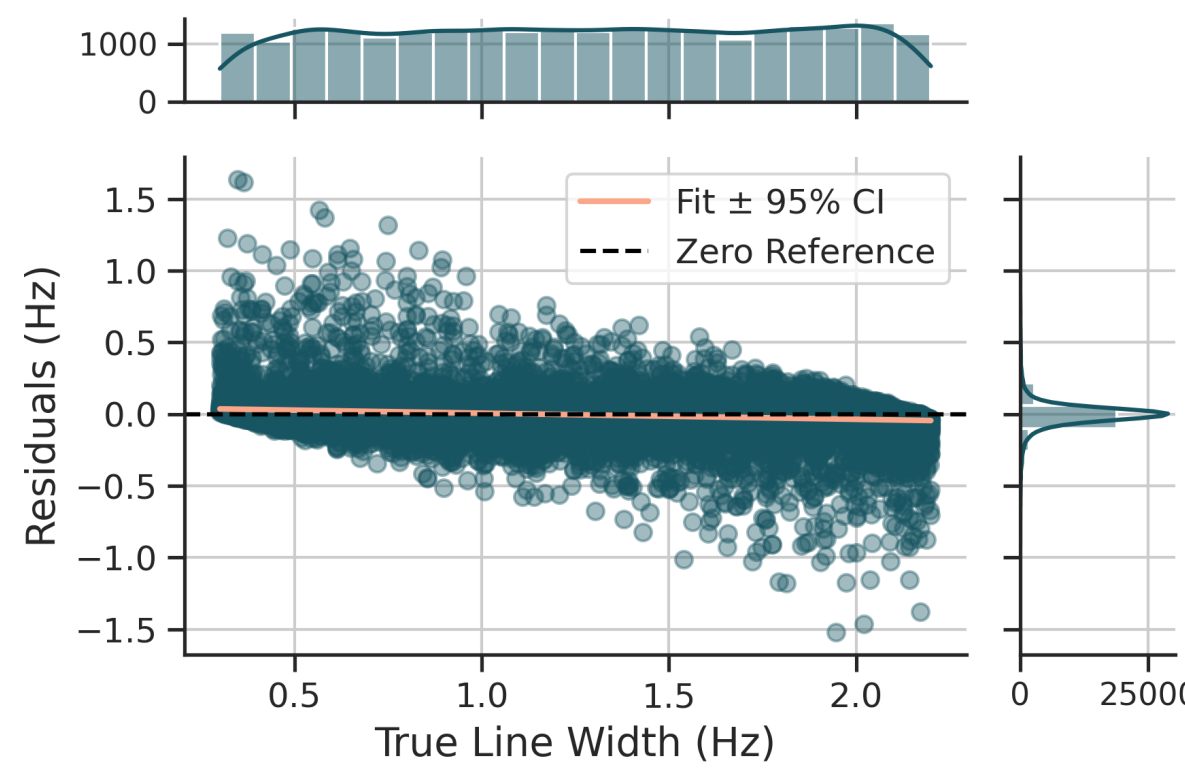

(d) Line width residuals

Figure S14: Evaluation metrics for strong coupling spin systems (1): This figure presents the accuracy of model predictions for key spectral parameters in strong coupling scenarios. (a) The predicted chemical shifts versus ground truth demonstrate the model's ability to predict resonance positions within complex, strongly coupled environments. (b) Residuals for chemical shift predictions provide an error analysis, revealing prediction consistency and any systematic deviations. (c) Predicted versus ground truth for line width assesses the model's precision in estimating line broadening, a challenging parameter in strong coupling conditions. (d) Line width residuals further break down these predictions, illustrating the model's stability across the dataset.

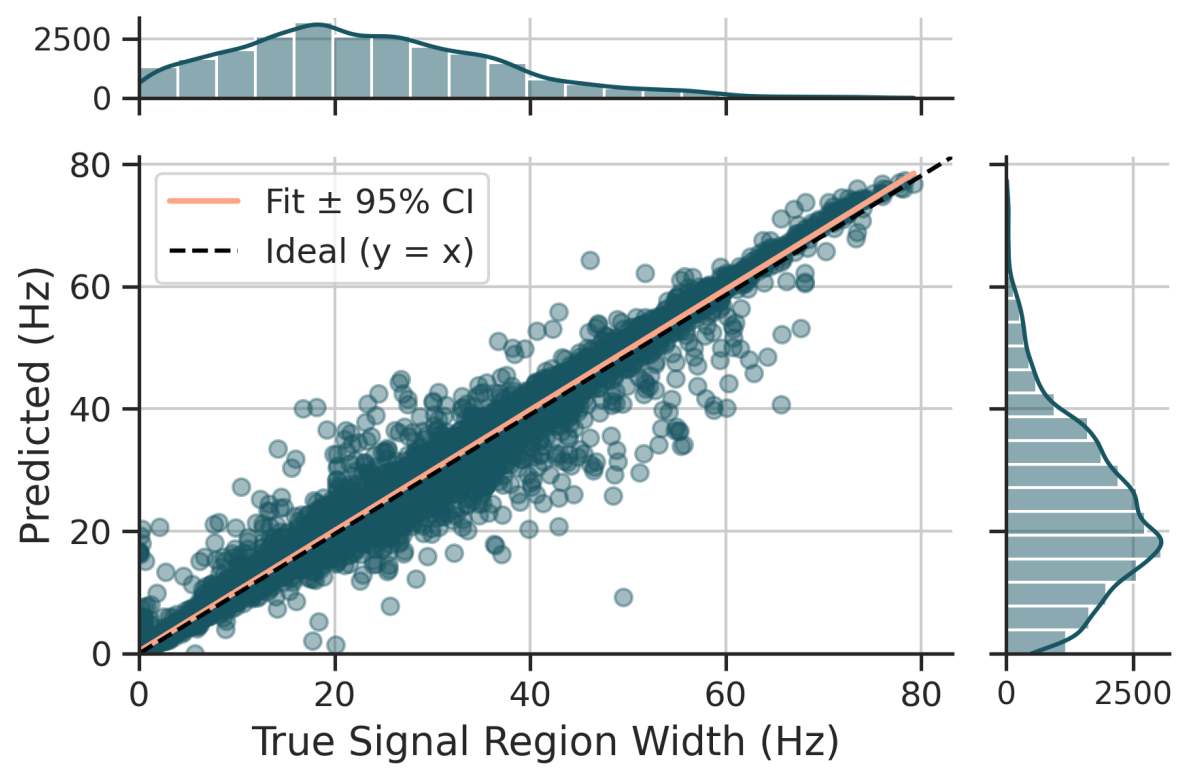

(a) Signal region predicted versus ground truth

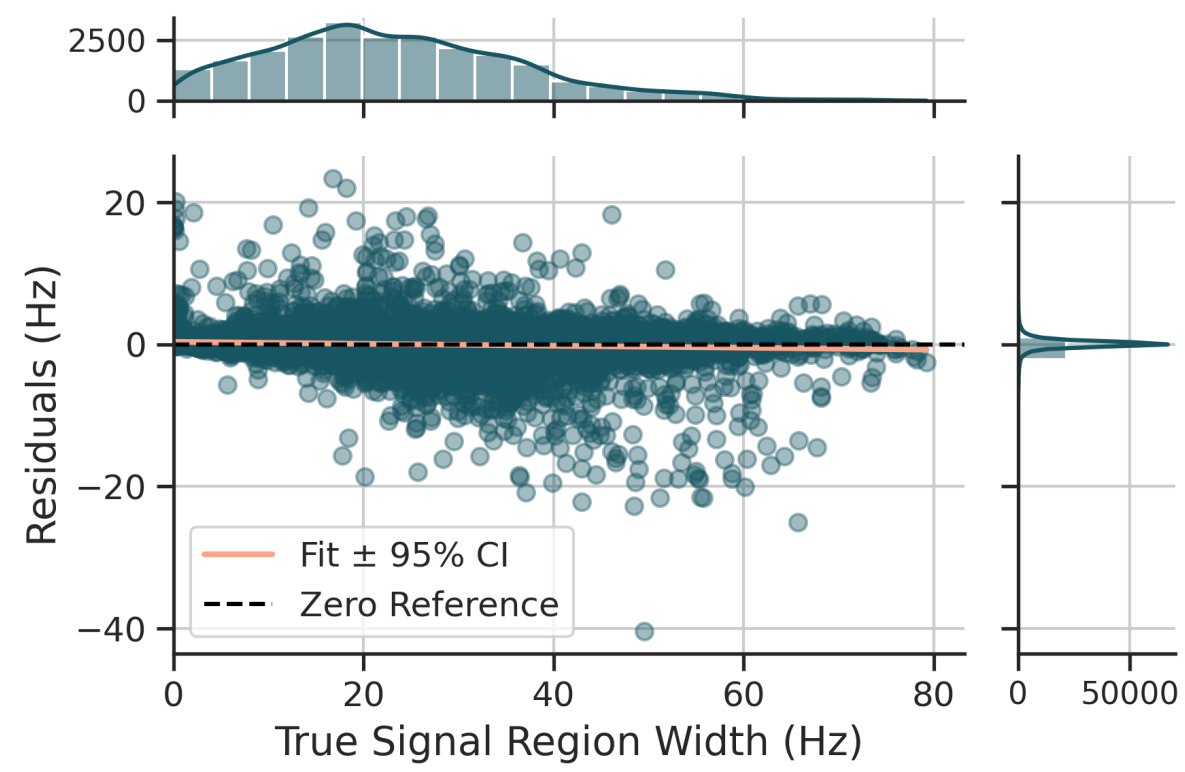

(b) Signal region residuals

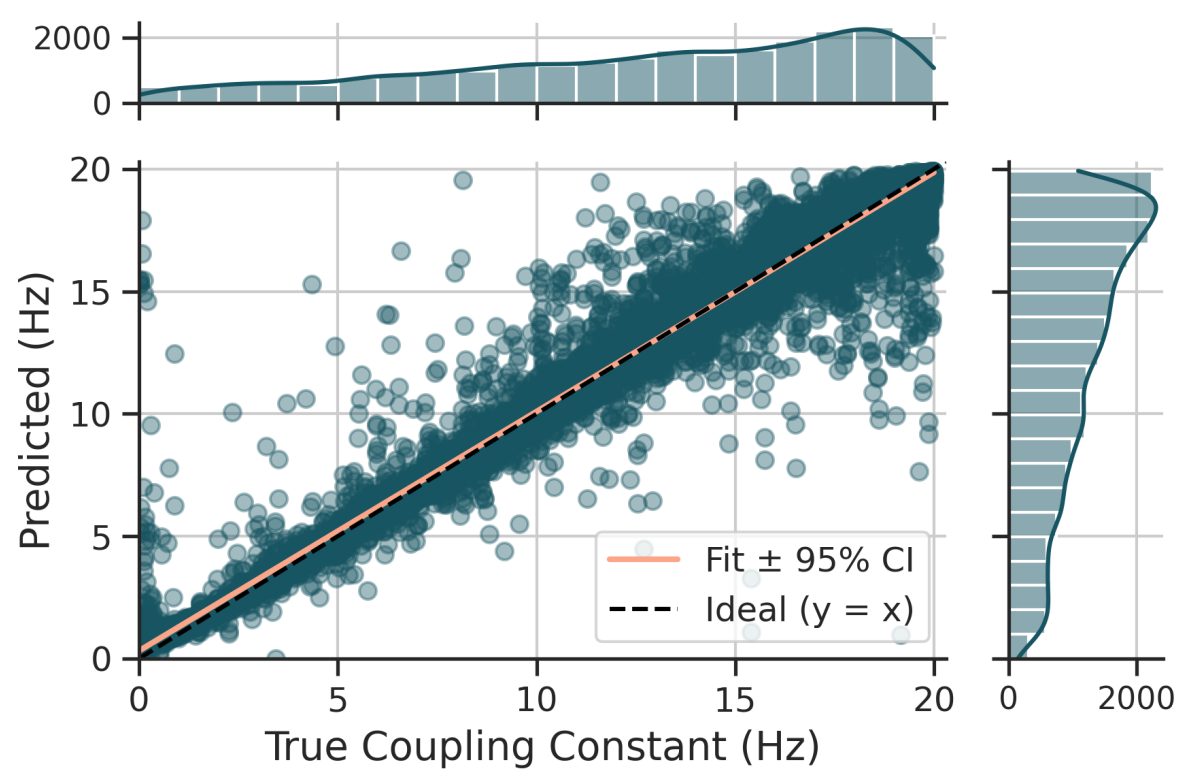

(c) Coupling constant predicted versus ground truth

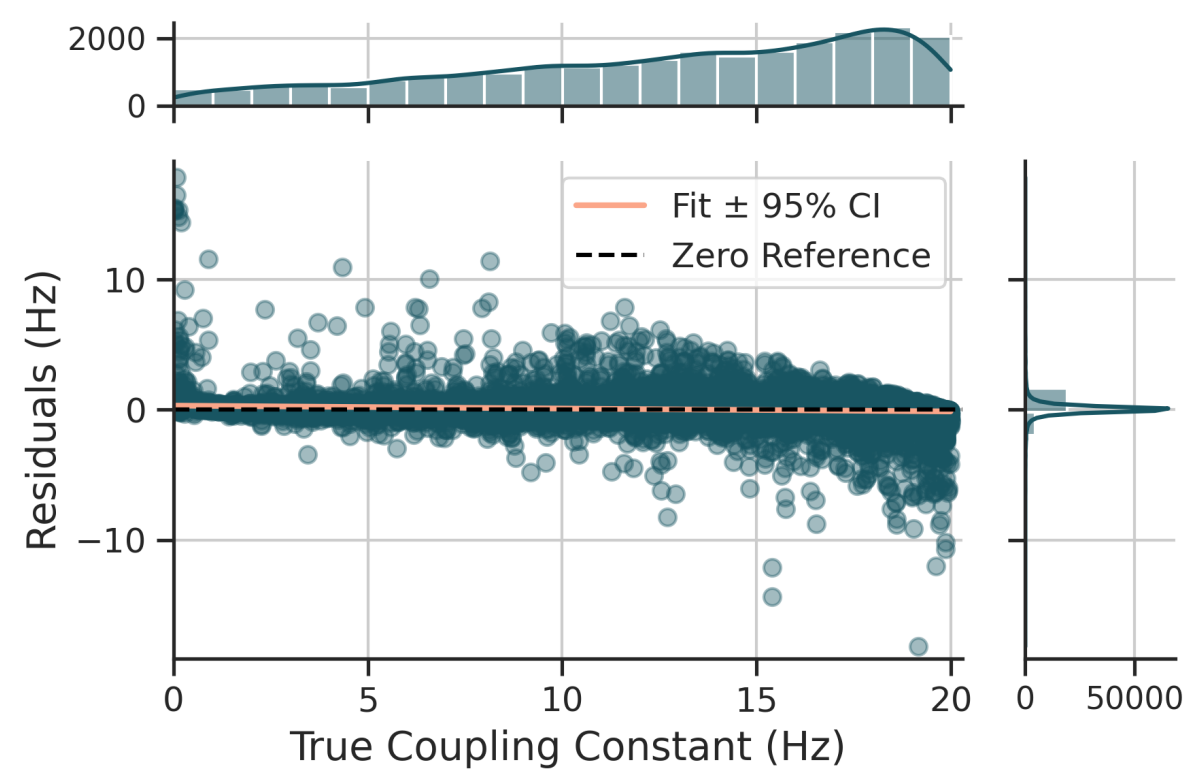

(d) Coupling constant residuals

Figure S15: Evaluation metrics for strong coupling spin systems (2): This figure expands the analysis to spatial and coupling interaction predictions. (a) Predicted signal region widths compared to ground truth reveal the model's capacity to accurately locate signals in the spectrum despite overlapping effects from strong coupling. (b) Residuals for signal region predictions highlight spatial accuracy and areas where model improvements could enhance prediction precision. (c) The coupling constant predictions versus ground truth underscore the model's effectiveness in identifying coupling strengths, which are more intricate in strongly coupled spin systems. (d) Coupling constant residuals provide detailed insights into prediction errors, helping to identify regions for refining coupling parameter estimations.

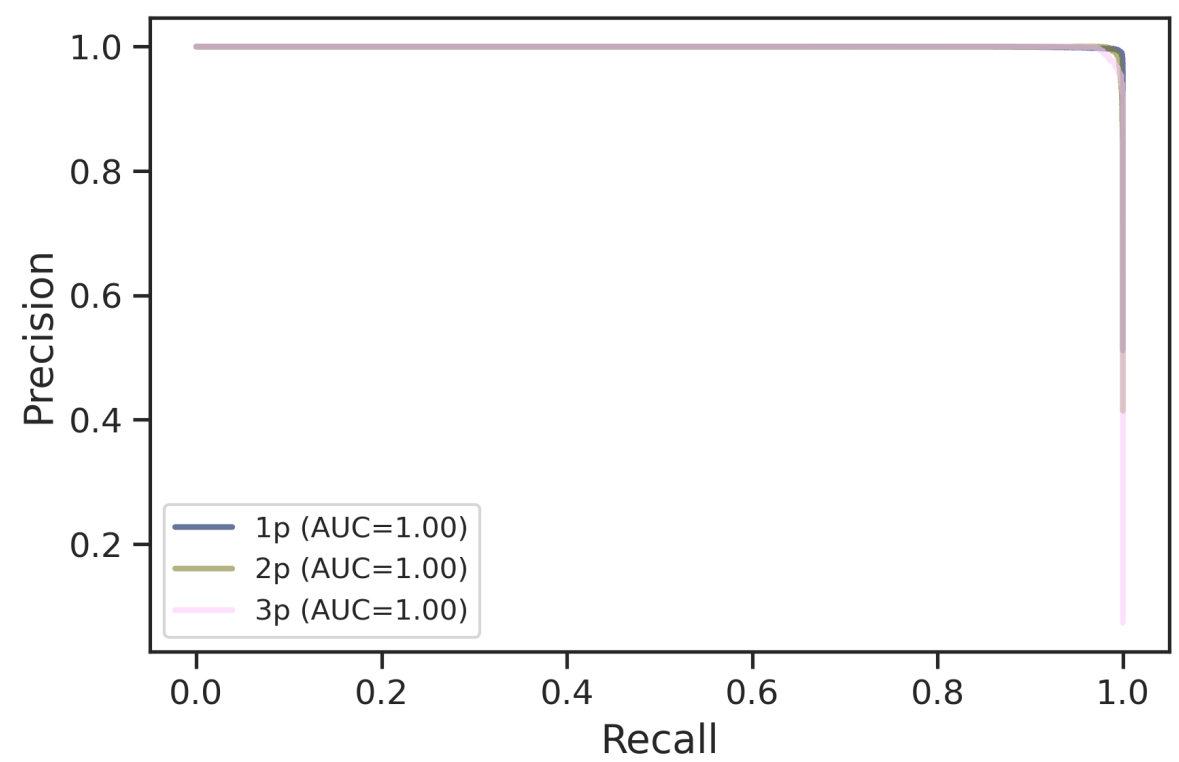

(a) Precision recall curve

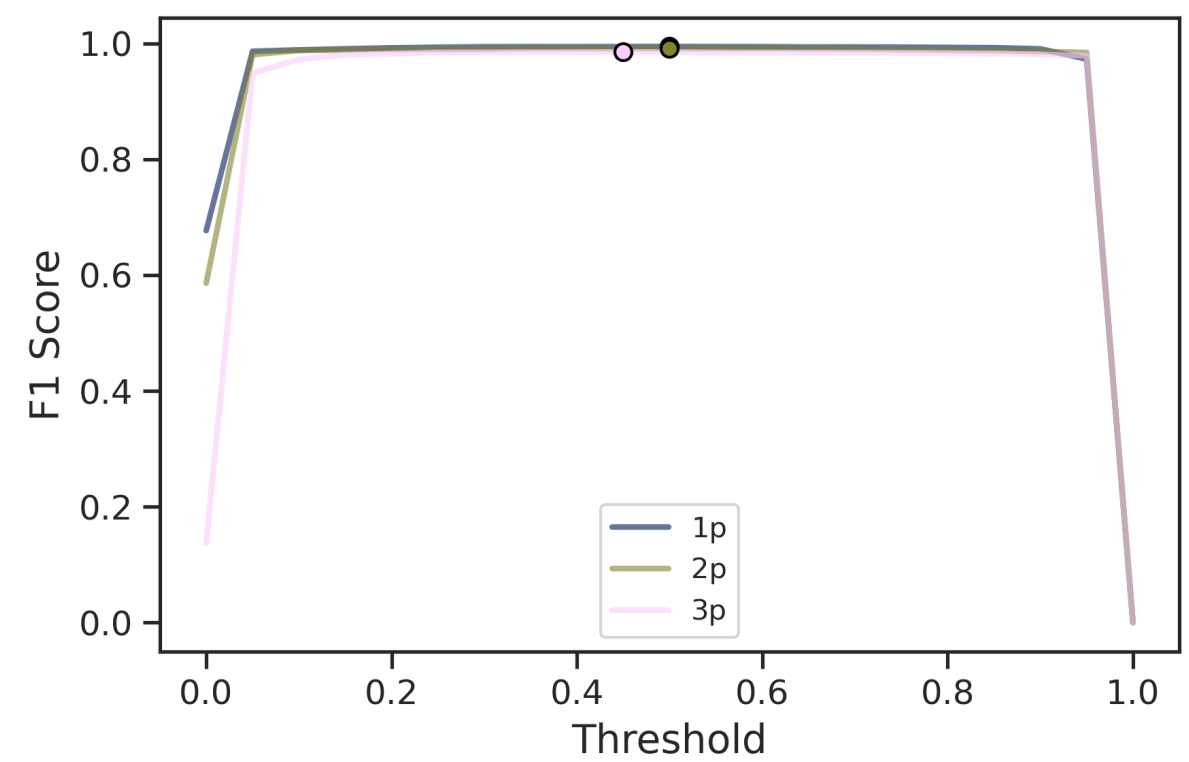

(b) F1 score versus threshold

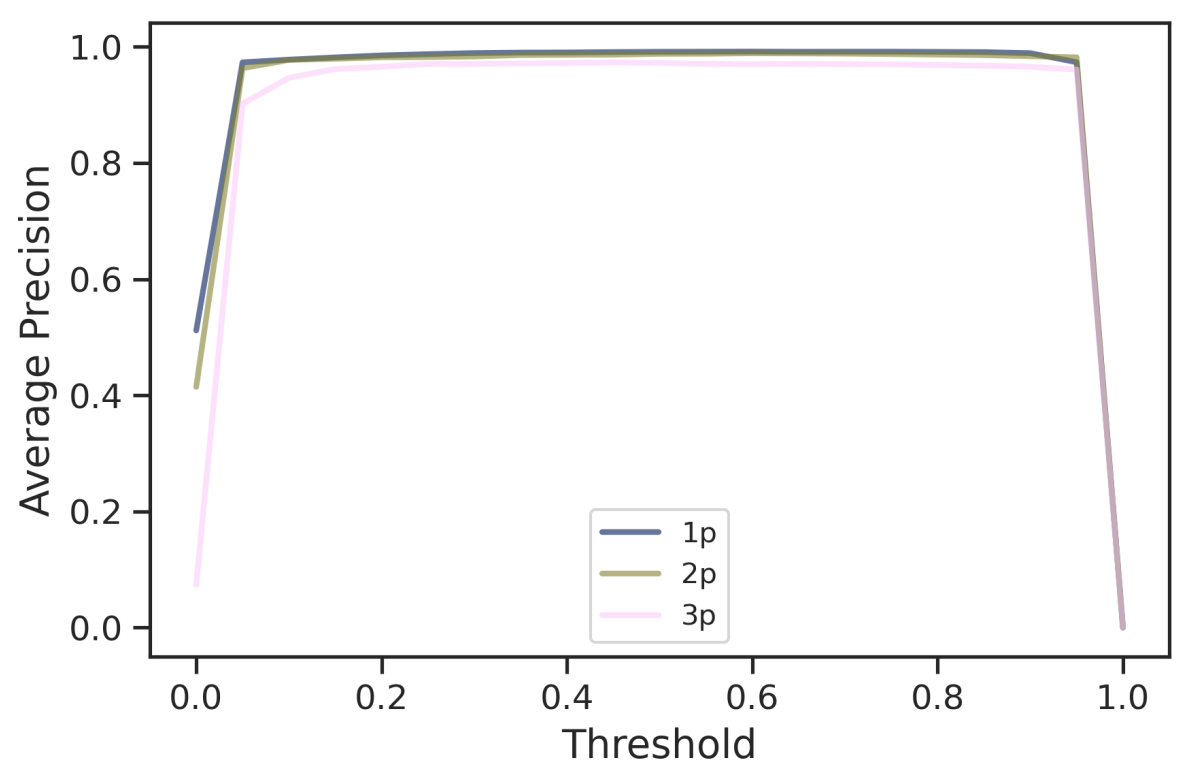

(c) Average precision versus threshold

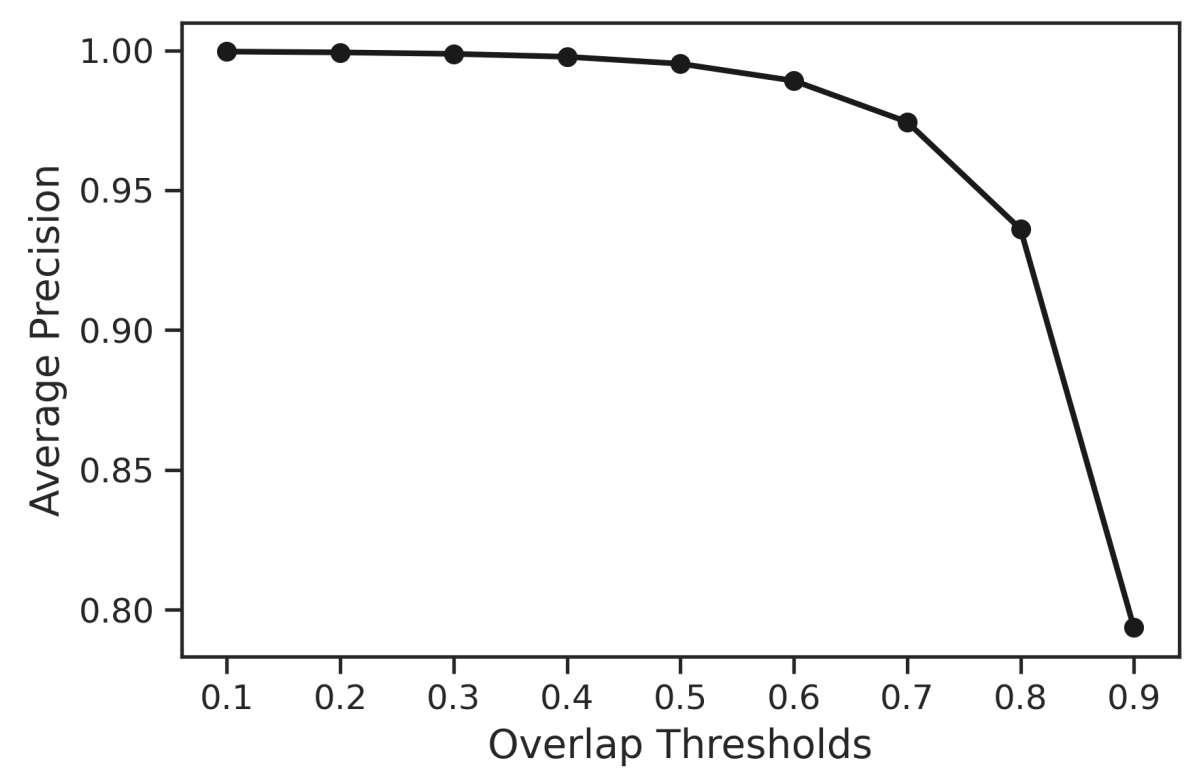

(d) Average precision for different IoU thresholds

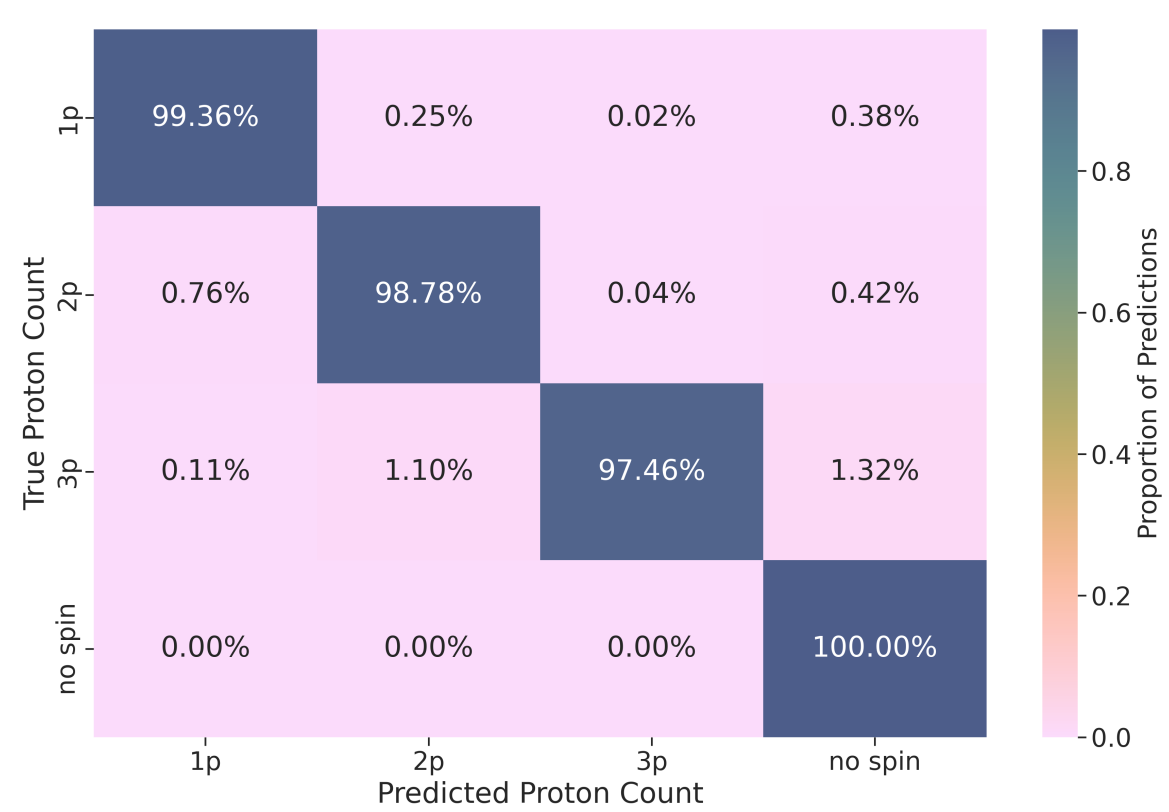

(e) Confusion matrix

Figure S16: Evaluation metrics for strong coupling spin systems (3): This figure displays the model's classification and threshold-dependent performance. (a) The precision-recall curve demonstrates the model's classification sensitivity and precision under strong coupling effects, which often obscure clear signal separation. (b) The F1 score against threshold shows how varying thresholds affect the balance between precision and recall, especially in complex systems. (c) Average precision versus threshold evaluates consistency across decision boundaries, a critical metric in high-noise environments. (d) Average precision at various IoU thresholds reveals the model's robustness across spatial criteria, essential in distinguishing overlapping spectral features. (e) The confusion matrix summarizes prediction outcomes, showing the model's ability to classify signals accurately while handling potential misclassifications in strongly coupled regions.

4.4 Experimental Test Spectra

The experimental test set comprised 19 spin systems from 12 distinct 1D ^{1}H NMR spectra, corresponding to 13 analyzed regions of interest (ROIs), 9 of which are aromatic. Across these systems, we curated 44 distinct magnetically equivalent spin groups (76 protons in total). Ground-truth assignments were obtained by expert interpretation guided by known molecular structures, databases, and DFT calculations and, when appropriate, validated by spin-system simulations. Of the 12 spectra, 7 were recorded on an 80 MHz benchtop spectrometer. The 80 MHz spectra were acquired at Bruker Switzerland AG; the high-field spectra (300, 500, and 600 MHz) were acquired at the University of Zurich. Experiment-specific metadata, including molecular structures and solvents, were provided by the original data sources at Bruker Switzerland AG and the University of Zurich. Spin-system annotations not available from these sources were completed by the authors, guided by known molecular structures, chemical-shift and coupling-constant prediction tools, and quantum-mechanical spin-system simulations. All annotations were independently verified by an external expert spectroscopist. Chemical shifts and proton counts are considered complete annotations. Coupling constants represent the dominant interaction per spin group (see Section 2.2 for the $\max(J)$ evaluation protocol); in strongly coupled or congested systems, only unambiguously extractable J-values were annotated. Unreported couplings reflect quantification limits, not physical absence. No experimental molecules, spectra, or molecular-specific information appear in the training data, which consists entirely of synthetic spectra with randomly sampled spin-system parameters (see Section 3.2). All spin system types represented in the test set (e.g., ABC, AA′BB′C, A_2X_3) are also present in the training data (Table S2), but the specific parameter values differ.

Before analysis, each spectrum was resampled to a constant points-per-Hz grid, and Gaussian line broadening ($\sigma = 0.4$ Hz) was applied. A spectrum may contribute one or more ROIs (for example, S5 provided two independent ROIs). ROIs were chosen to fully contain the annotated spin systems; phenomena outside the scope of this study (for example, unrelated signals far outside the annotated region) were not included. If an analyzed ROI contained fewer than the standard window of 6144 points, the remainder of the window was zero-padded; the ppm ranges listed in Table S7 refer to the actual (unpadded) spectrum points. Concentrations for the historical test spectra were not provided by the original sources. MolDeTr's predictions are robust to absolute concentration because inputs are normalized on a per-spectrum basis and amplitudes are randomized during training (SI Sections 4.2 and 3.2.2).

For visual reference, the ROI plots are shown in Figs. S17–S21. In each panel, the blue trace is the normalized experimental ROI, and green dashed vertical lines mark the curated chemical shifts δ (one line per spin group). Proton counts (nH) and coupling constants (J) follow the curation annotations used to establish ground truth.

Table S7: Experimental test set (12 spectra; 13 ROIs; 19 curated spin systems; 44 spin groups, 76 protons). The analyzed region (ppm) reports the actual (unpadded) chemical-shift range used for evaluation. For 80 MHz benchtop spectra without deuterium lock (S1, S2, S6, S7), ppm values reflect the raw receiver reference and may include global offsets relative to TMS. MolDeTr is reference-agnostic (see Methods).

Spectrum	ROI	Compound	Solvent	Base freq. (MHz)	Analyzed region (ppm)
S1	R1	Ethyl Acetate	—	80.155	[-8.57, 6.41]
S2	R1	Ethylbenzene	CDCl3	80.153	[-4.57, 10.40]
S3	R1	Ethyl-cis-3-iodacrylate	CDCl3	80.147	[-3.61, 11.36]
S4	R1	Ibuprofen	—	80.148	[5.17, 10.05]
S5	R1	Ethyl Vanillin	—	80.147	[-3.89, 5.27]
S5	R2	Ethyl Vanillin	—	80.147	[6.73, 7.95]
S6	R1	Ethyl Acetate	—	80.155	[-8.68, 1.53]
S7	R1	Ethylbenzene	CDCl3	80.153	[0.65, 10.40]
S8	R1	Vanillin	DMSO-d6	300.130	[6.29, 8.56]
S9	R1	Cinnamic Acid	CDCl3	300.130	[7.28, 7.74]
S10	R1	Guajazulene	C6D6	500.130	[7.29, 8.85]
S12	R1	Cocaine	D2O	600.130	[6.89, 8.89]
S13	R1	Caffeic Acid	acetone-d6	600.130	[5.91, 7.91]

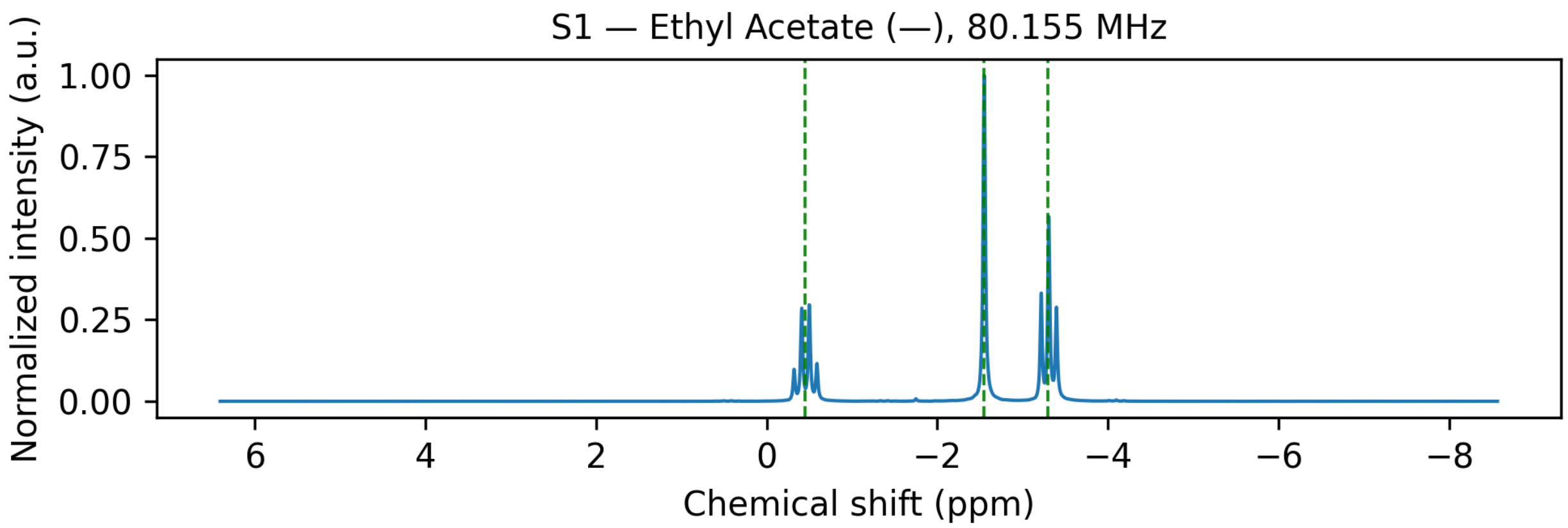

(a) S1 — Ethyl Acetate, 80.155 MHz. ROI R1 [-8.57, 6.41]. Annotations: g1, 2H, δ=-0.450 ppm, J=7.13 Hz; g2, 3H, δ=-2.550 ppm; g3, 3H, δ=-3.300 ppm, J=7.13 Hz.

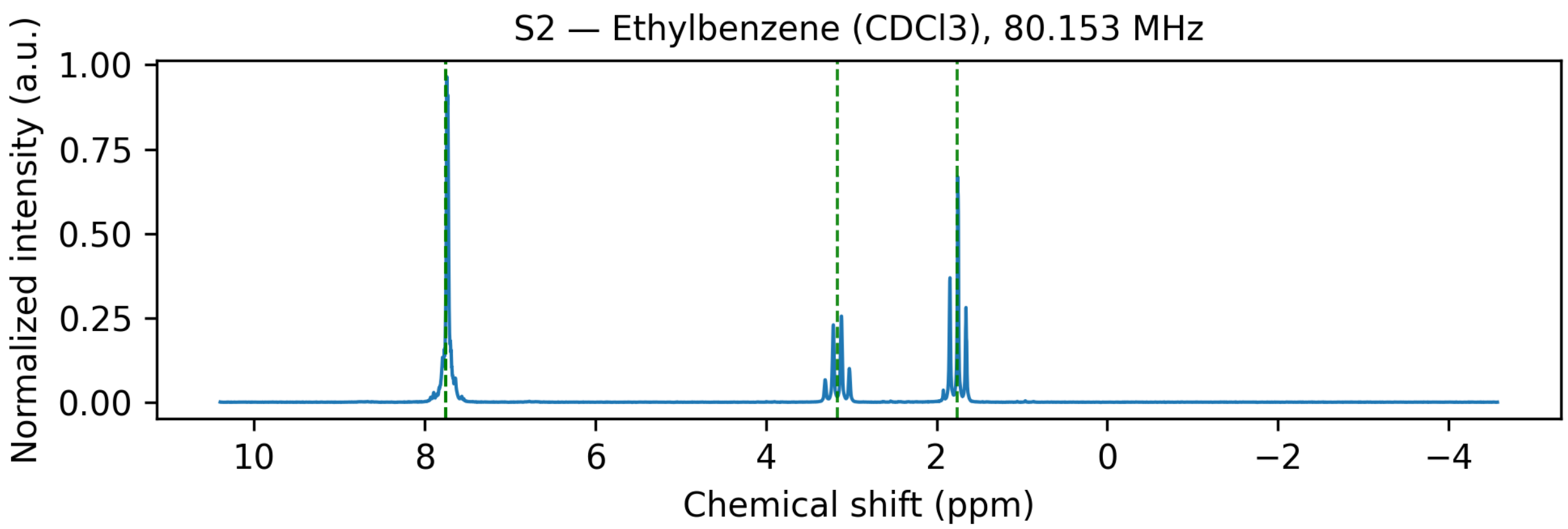

(b) S2 — Ethylbenzene, 80.153 MHz, $CDCl_3$. ROI R1 [-4.57, 10.40]. Annotations: g1, 2H, δ=7.750 ppm; g2, 2H, δ=7.750 ppm; g3, 1H, δ=7.750 ppm; g4, 2H, δ=3.166 ppm, J=7.53 Hz; g5, 3H, δ=1.756 ppm, J=7.60 Hz (duplicate δ values denote distinct, magnetically inequivalent spin groups).

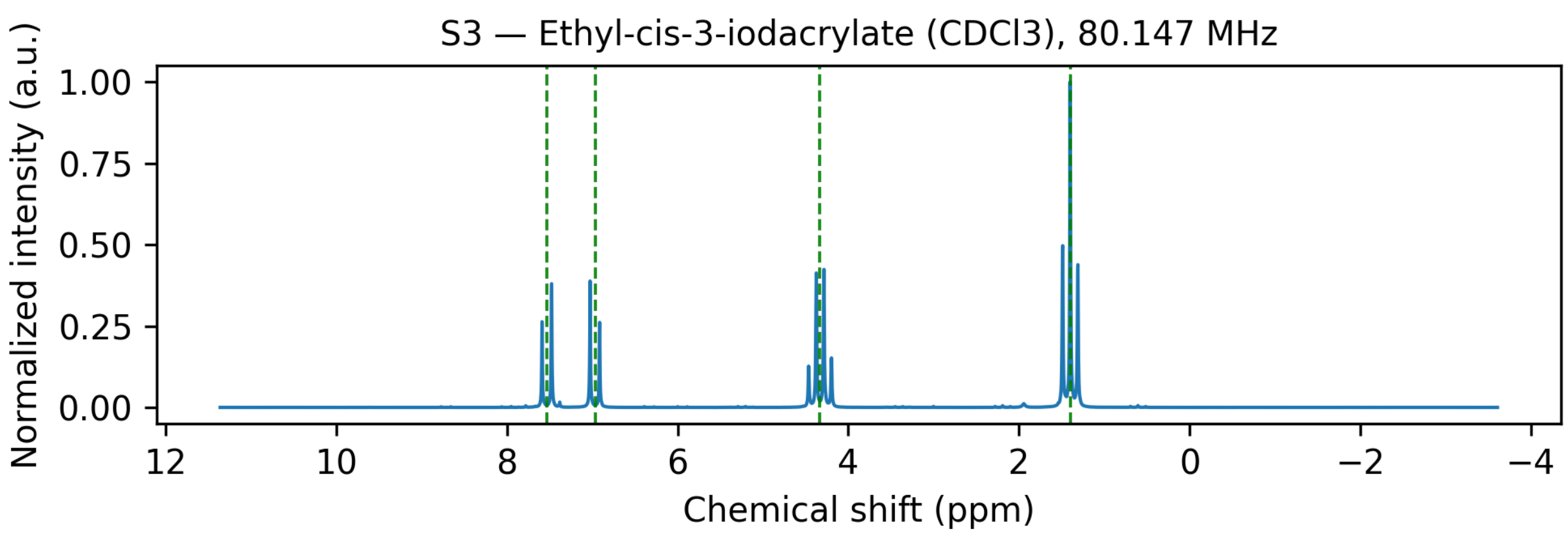

(c) S3 — Ethyl-cis-3-iodacrylate, 80.147 MHz, $CDCl_3$. ROI R1 [-3.61, 11.36]. Annotations: g1, 1H, δ=7.533 ppm, J=8.84 Hz; g2, 1H, δ=6.965 ppm, J=8.90 Hz; g3, 2H, δ=4.340 ppm, J=7.10 Hz; g4, 3H, δ=1.400 ppm, J=7.00 Hz.

Figure S17: Experimental regions of interest (ROIs), panels 1–3 of 13. ROI trace (blue), curated chemical shifts δ (green dashed lines). Annotations use g# to denote magnetically equivalent spin groups, with reported proton count, chemical shift (ppm), and J couplings (Hz). Intensities are normalized per panel; the x-axis follows the NMR convention (increasing δ from right to left).

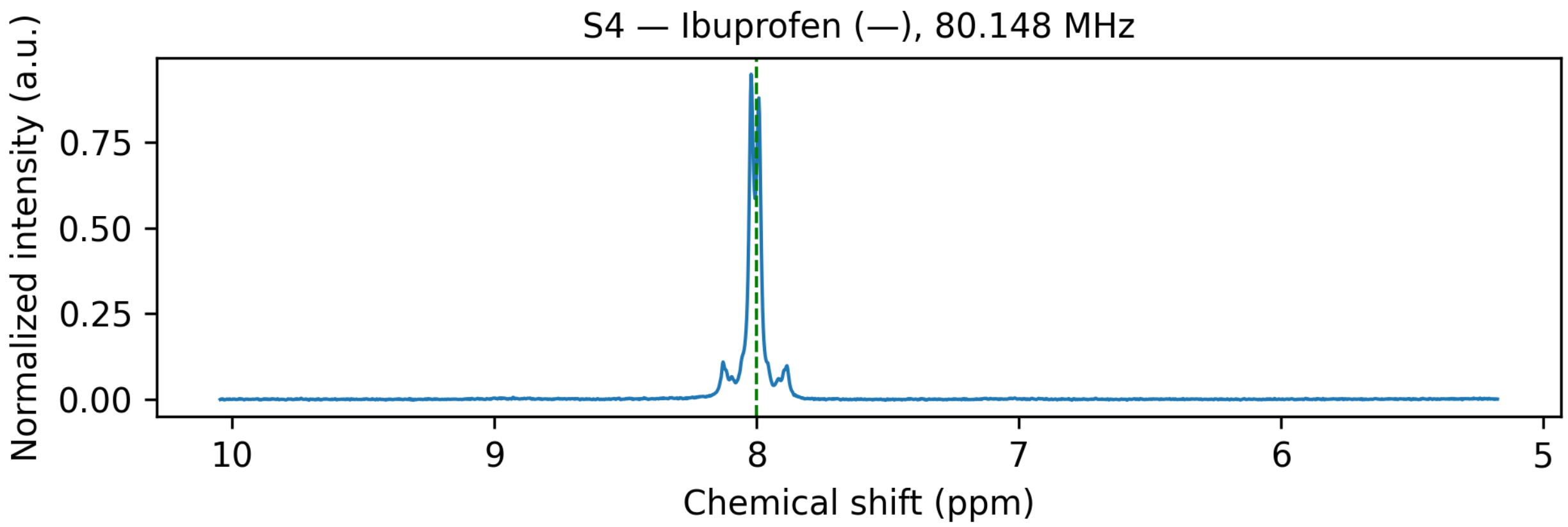

(a) S4 — Ibuprofen, 80.148 MHz. ROI R1 [5.17, 10.05]. Annotations: g1, 2H, δ=8.000 ppm; g2, 2H, δ=8.000 ppm (duplicate δ values denote distinct, magnetically inequivalent spin groups).

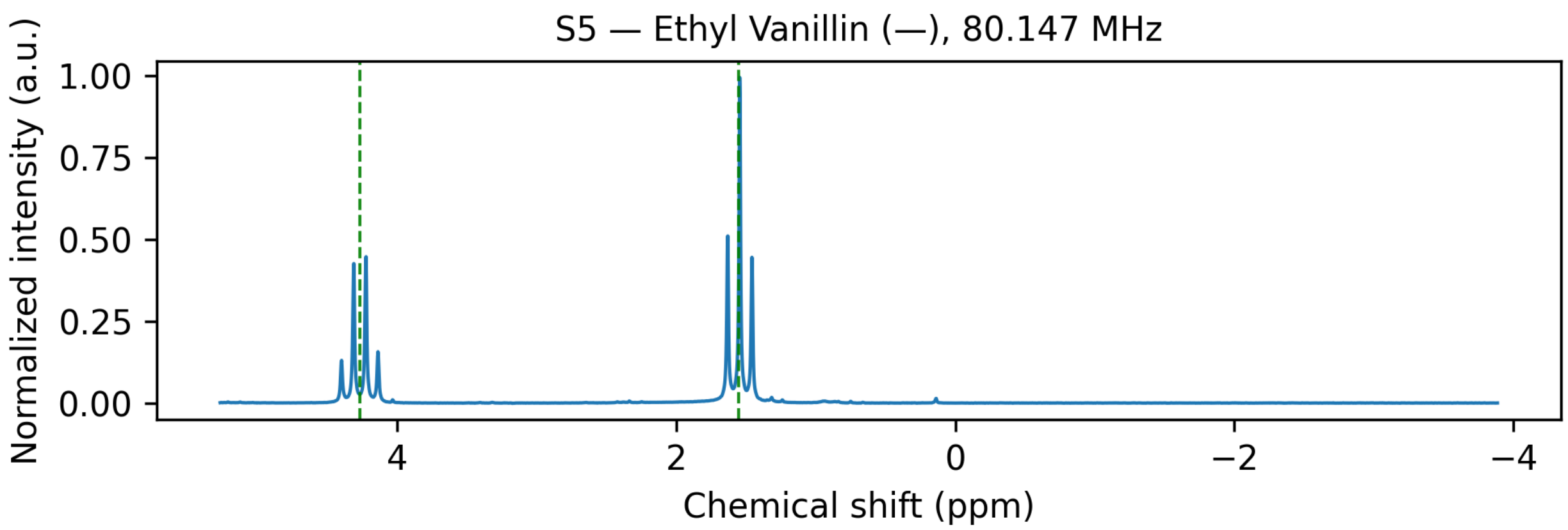

(b) S5 — Ethyl Vanillin, 80.147 MHz. ROI R1 [-3.89, 5.27]. Annotations: g1, 2H, δ=4.270 ppm, J=6.96 Hz; g2, 3H, δ=1.550 ppm, J=7.00 Hz.

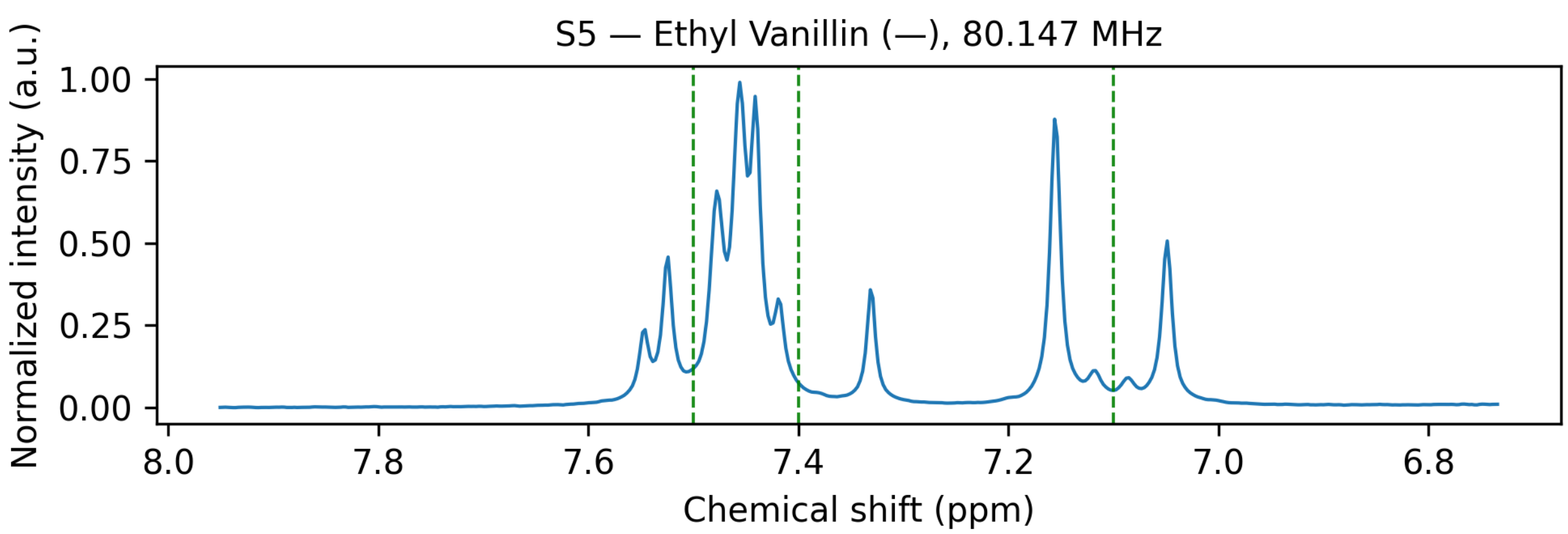

(c) S5 — Ethyl Vanillin, 80.147 MHz. ROI R2 [6.73, 7.95]. Annotations: g1, 1H, δ=7.500 ppm; g2, 1H, δ=7.400 ppm; g3, 1H, δ=7.100 ppm.

Figure S18: Experimental ROIs, panels 4–6 of 13. ROI trace (blue), curated chemical shifts δ (green dashed lines). g# denotes magnetically equivalent spin groups. Intensities normalized per panel.

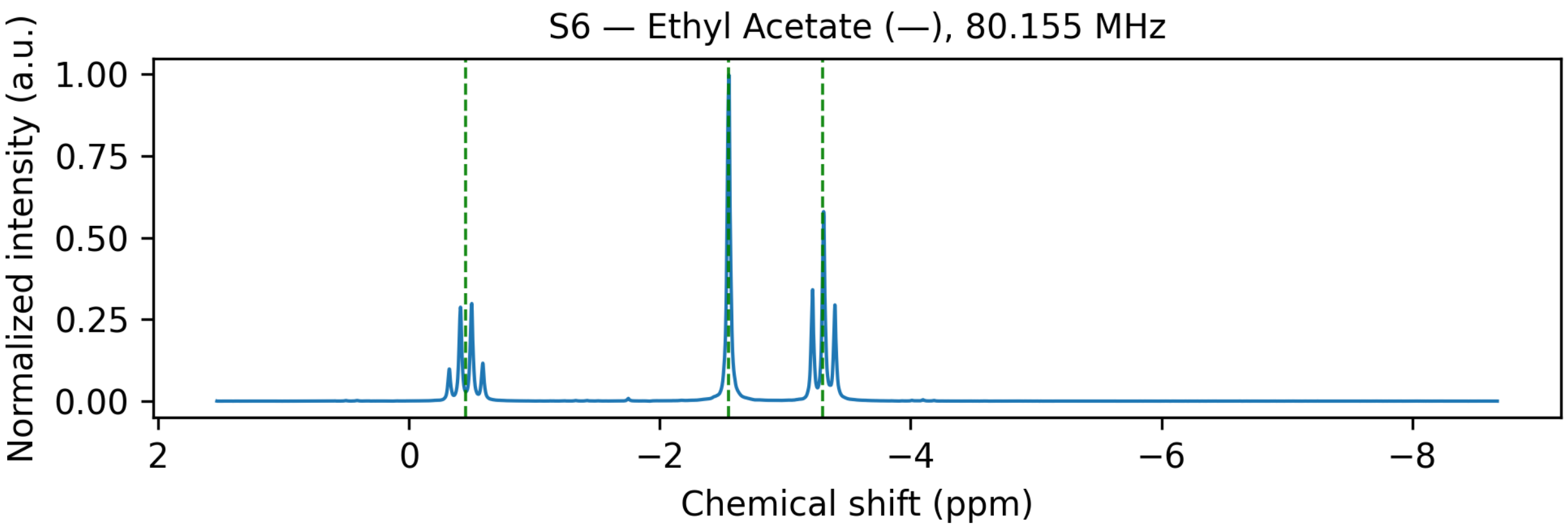

(a) S6 — Ethyl Acetate, 80.155 MHz. ROI R1 [-8.68, 1.53]. Annotations: g1, 2H, δ=-0.450 ppm, J=7.13 Hz; g2, 3H, δ=-2.550 ppm; g3, 3H, δ=-3.300 ppm, J=7.14 Hz.

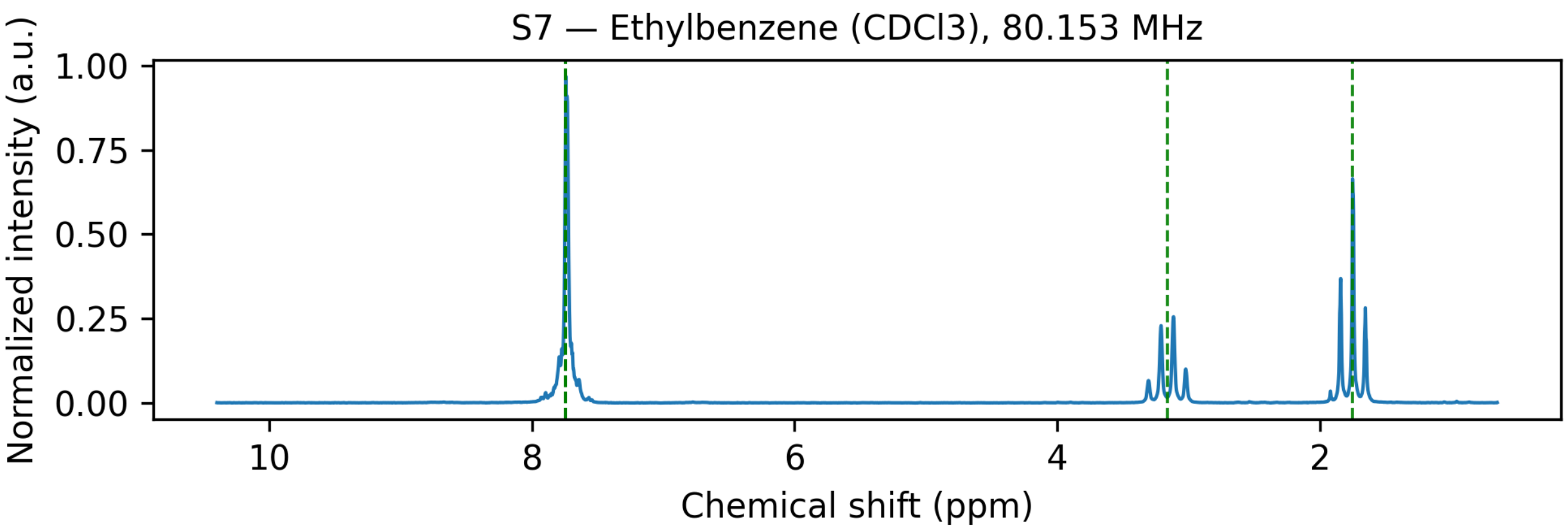

(b) S7 — Ethylbenzene, 80.153 MHz, $CDCl_3$. ROI R1 [0.65, 10.40]. Annotations: g1, 2H, δ=7.745 ppm; g2, 2H, δ=7.745 ppm; g3, 1H, δ=7.745 ppm; g4, 2H, δ=3.164 ppm, J=7.65 Hz; g5, 3H, δ=1.754 ppm, J=7.65 Hz (duplicate δ values denote distinct, magnetically inequivalent spin groups).

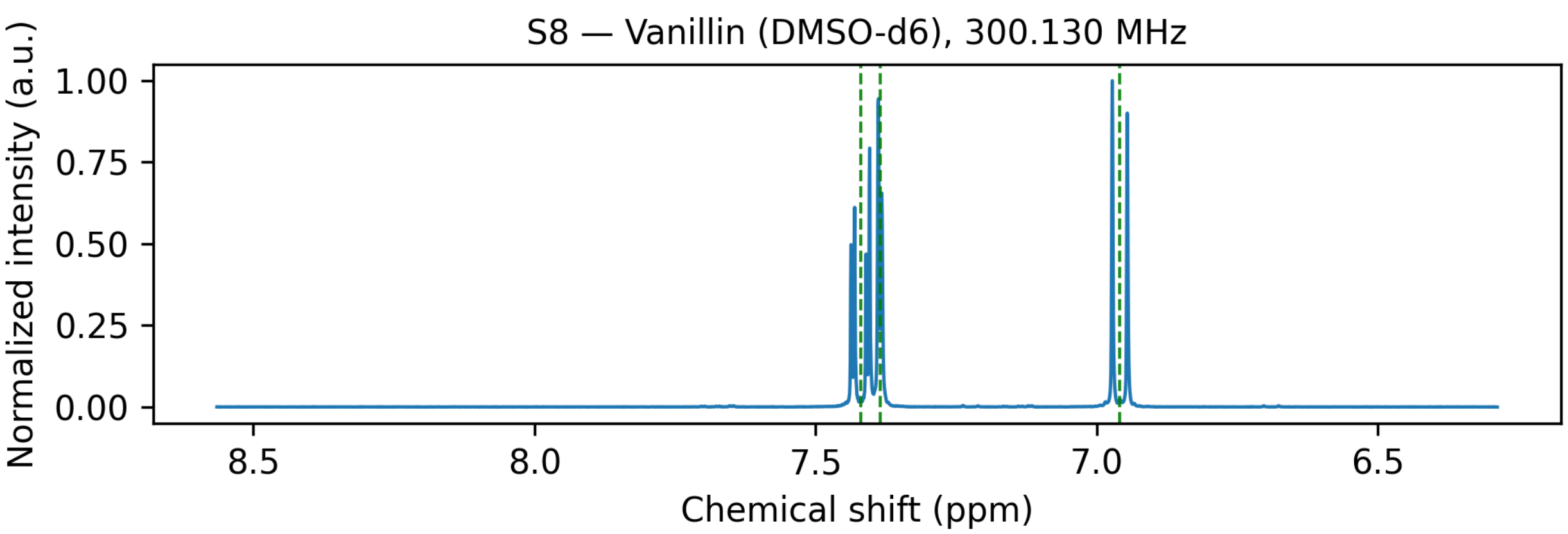

(c) S8 — Vanillin, 300.130 MHz, DMSO-d_6. ROI R1 [6.29, 8.56]. Annotations: g1, 1H, δ=7.420 ppm, J=8.10 Hz; g2, 1H, δ=7.385 ppm, J=2.00 Hz; g3, 1H, δ=6.959 ppm, J=8.10 Hz.

Figure S19: Experimental ROIs, panels 7–9 of 13. ROI trace (blue), curated chemical shifts δ (green dashed lines). g# denotes magnetically equivalent spin groups. Intensities normalized per panel.

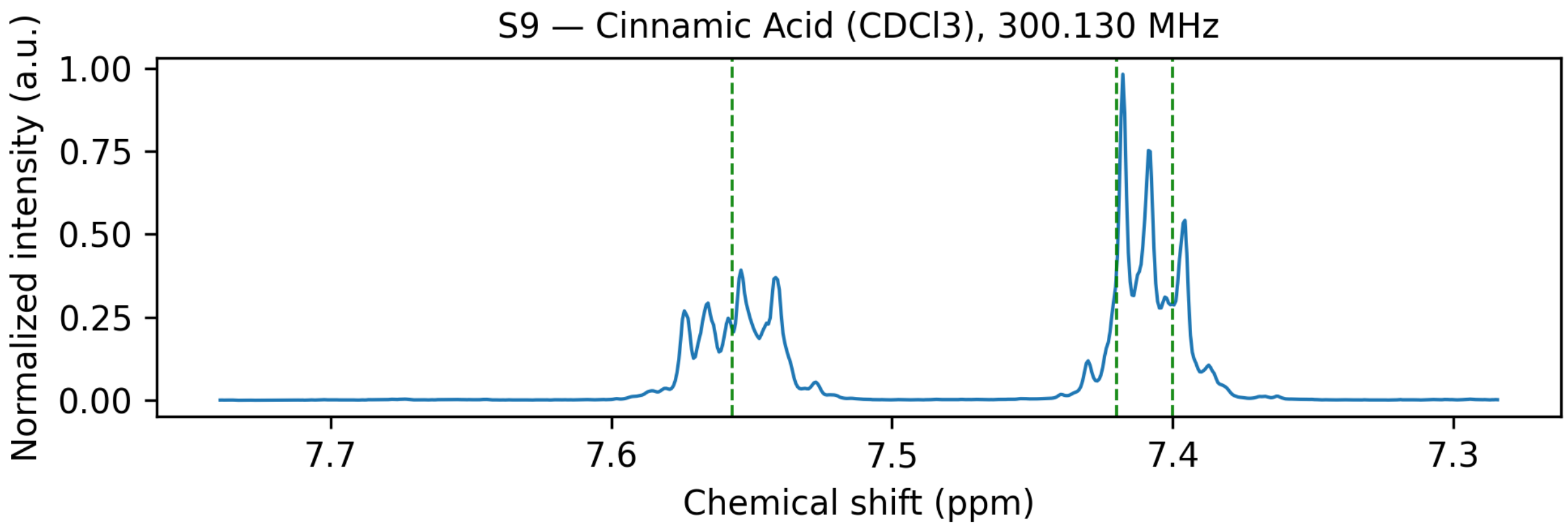

(a) S9 — Cinnamic Acid, 300.130 MHz, $CDCl_3$. ROI R1 [7.28, 7.74]. Annotations: g1, 2H, δ=7.557 ppm; g2, 1H, δ=7.420 ppm; g3, 2H, δ=7.400 ppm.

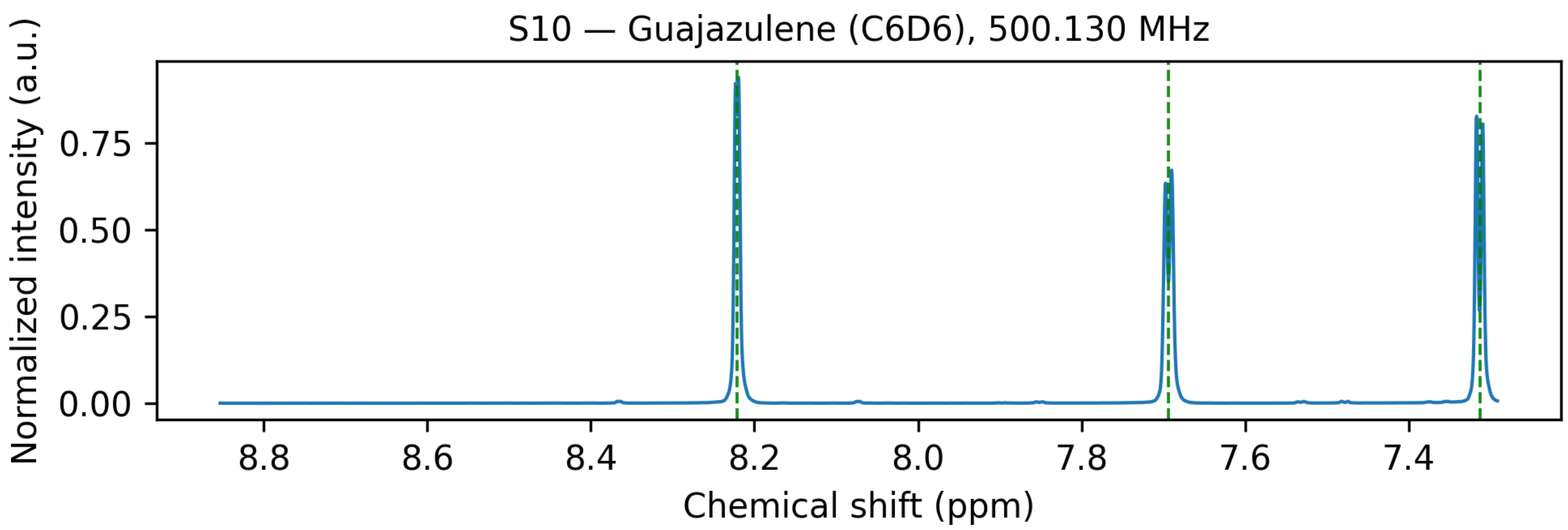

(b) S10 — Guajazulene, 500.130 MHz, C_6D_6. ROI R1 [7.29, 8.85]. Annotations: g1, 1H, δ=8.221 ppm, J=1.80 Hz; g2, 1H, δ=7.694 ppm, J=3.70 Hz; g3, 1H, δ=7.313 ppm, J=3.70 Hz.

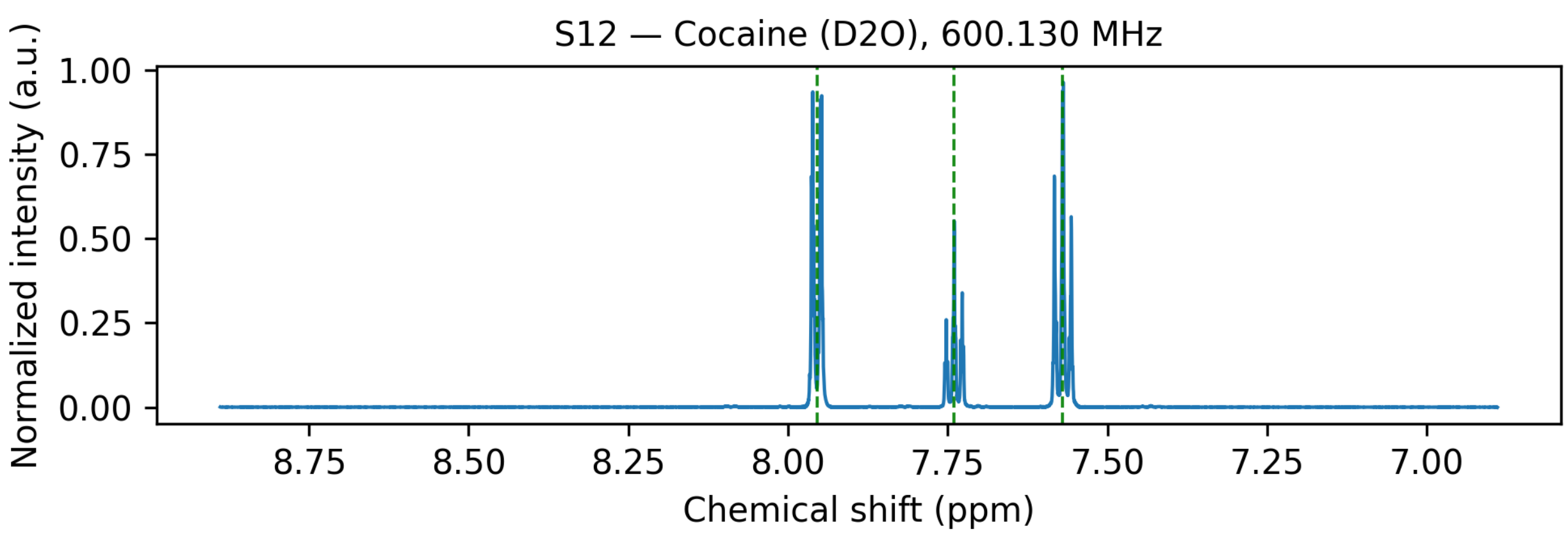

(c) S12 — Cocaine, 600.130 MHz, D_2O. ROI R1 [6.89, 8.89]. Annotations: g1, 2H, δ=7.955 ppm; g2, 1H, δ=7.740 ppm; g3, 2H, δ=7.570 ppm.

Figure S20: Experimental ROIs, panels 10–12 of 13. ROI trace (blue), curated chemical shifts δ (green dashed lines). g# denotes magnetically equivalent spin groups. Intensities normalized per panel.

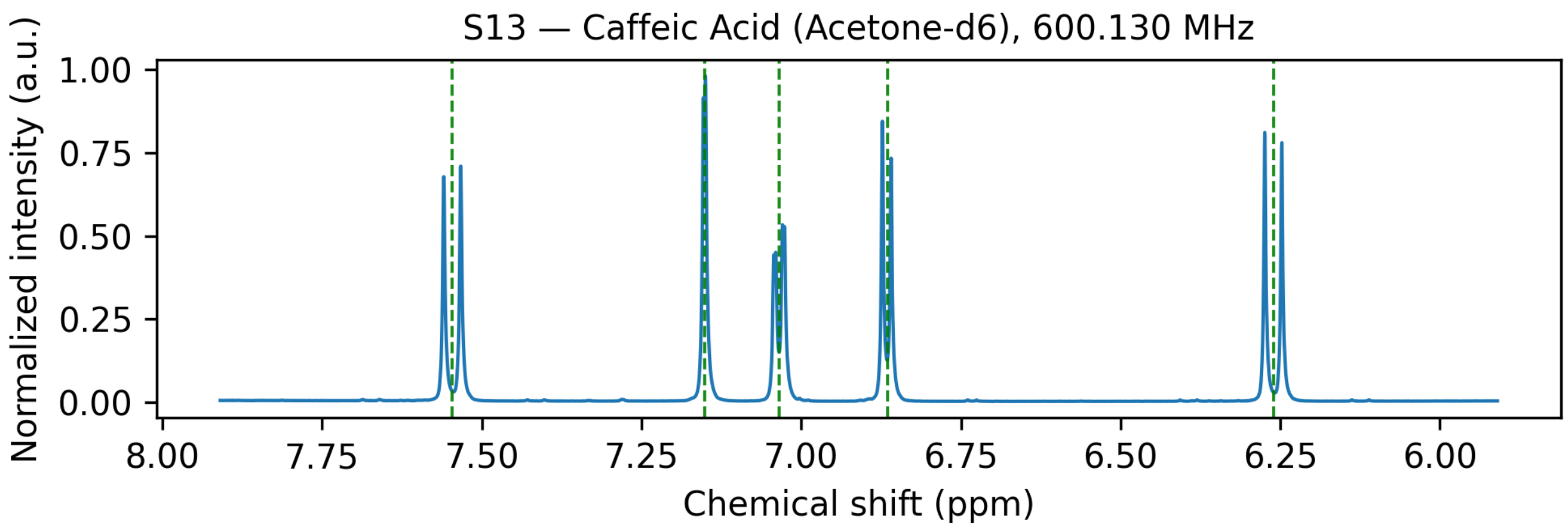

(a) S13 — Caffeic Acid, 600.130 MHz, acetone-d_6. ROI R1 [5.91, 7.91]. Annotations: g1, 1H, δ=7.546 ppm, J=16.00 Hz; g2, 1H, δ=7.151 ppm, J=2.20 Hz; g3, 1H, δ=7.035 ppm, J=8.30 Hz; g4, 1H, δ=6.865 ppm, J=8.10 Hz; g5, 1H, δ=6.260 ppm, J=16.20 Hz.

Figure S21: Experimental ROIs, panel 13 of 13. ROI trace (blue), curated chemical shifts δ (green dashed lines). g# denotes magnetically equivalent spin groups. Intensities normalized per panel.

4.5 Confusion Matrix for Proton Count Predictions on Experimental Spectra

The confusion matrix (Figure S22) demonstrates high classification accuracy across proton count classes, with correct identification rates of 97.00% for single protons, 88.75% for two-proton systems, 75.00% for three-proton systems, and 95.35% for no-spin cases. Notably, four- and six-proton systems were absent from the experimental test set.

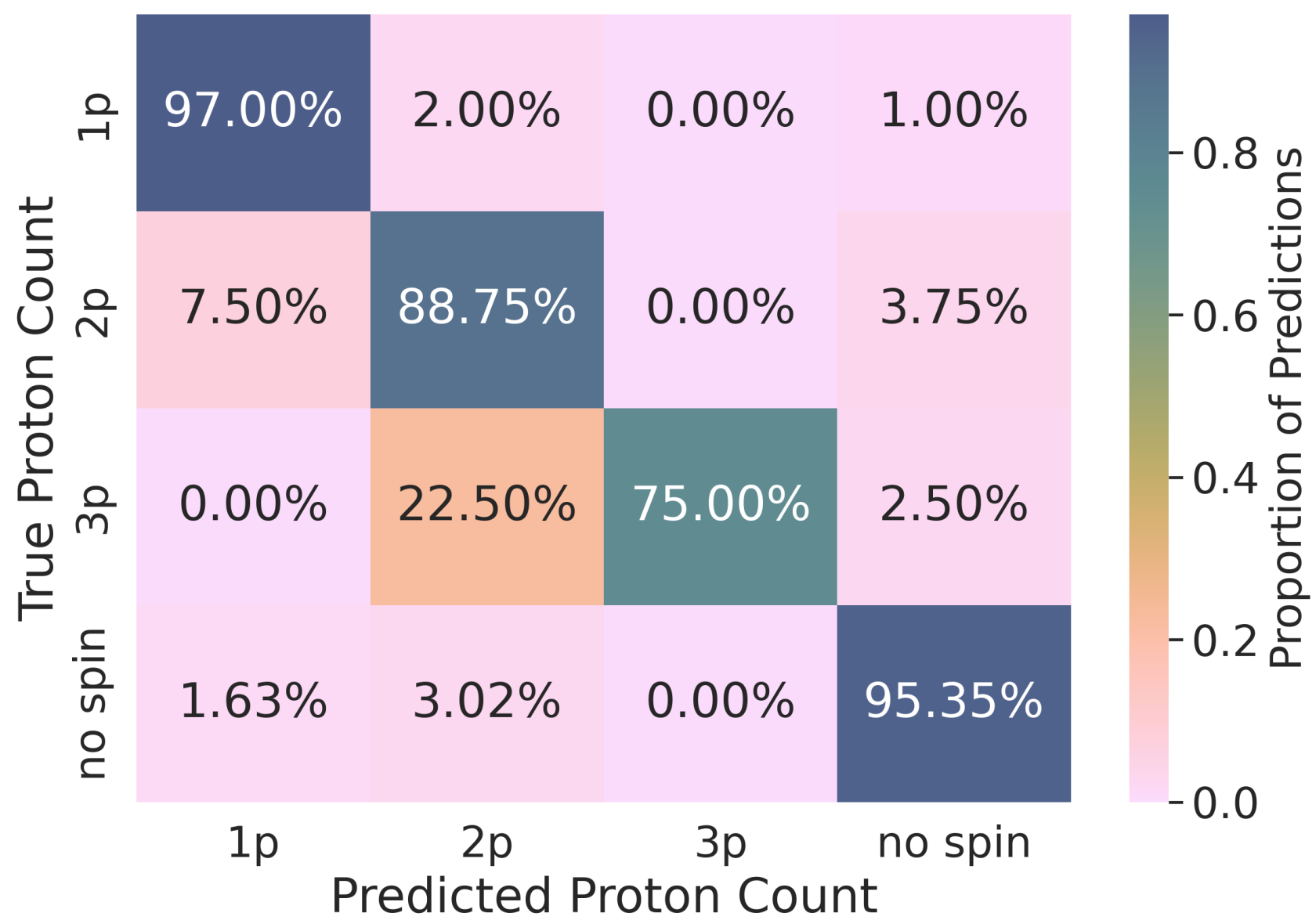

Figure S22: Confusion matrix for proton count predictions, showing classification performance across proton classes in experimental spectra.

4.6 Experimental Case Study of the Aromatic Region of Cinnamic Acid

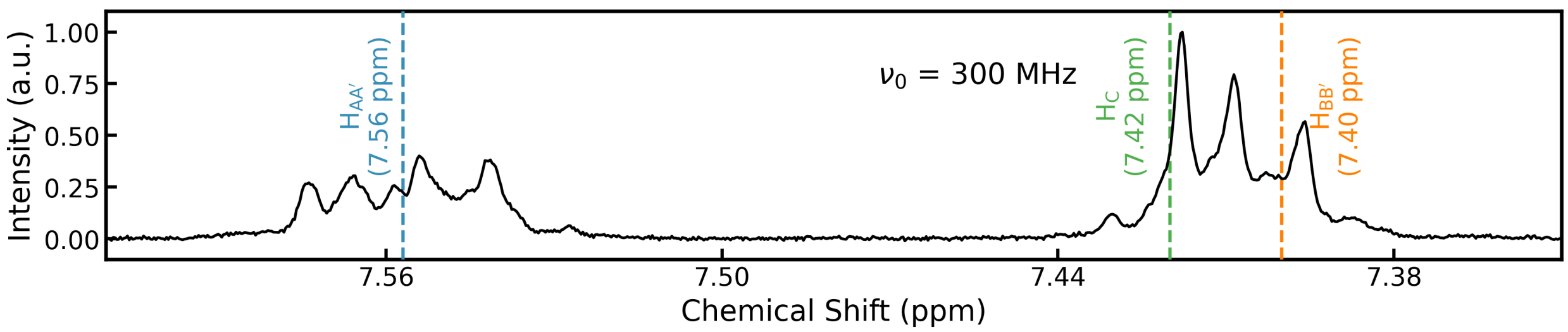

Figure S23: **Cinnamic Acid Aromatic Region.** A ^{1}H NMR spectrum acquired at 300 MHz, highlighting the `AA′BB′C` spin system. Multiple strong coupling effects cause overlapping resonances, with dashed, color-coded vertical lines denoting ground truth chemical shifts.

In cinnamic acid's aromatic region, the `AA′BB′C` spin system exhibits a complex multiplet pattern due to strong coupling interactions between multiple protons. Although the overlapping resonances present a challenge for conventional analysis, MolDeTr accurately identified the underlying spin system from data recorded at 300 MHz, reproducing chemical shifts that closely match the ground truth reference values (Figure S23). Specifically, MolDeTr predicted chemical shifts for the `AA′` (7.56 ppm), `BB′` (7.41 ppm), and `C` (7.41 ppm), which demonstrate its ability to resolve complex aromatic coupling networks (Table S8).

For simplicity, symmetrical coupling constants were assumed (e.g., $J_{\text{A-B}} = J_{\text{A}'\text{-B}'}$). *It is important to note that the ground truth coupling constants serve as approximate measures, due to the inherent difficulties in precisely fitting this strongly coupled system. The chemical shifts were rounded to two decimal places.

Table S8: Comparison of Ground Truth and MolDeTr Predictions for the Cinnamic Acid Aromatic Region (`AA′BB′C` Spin System) at 300 MHz.

**Spin**	**Proton Count**	**Chemical Shift (ppm)**	**J-Couplings (Hz)**
		**Ground Truth**	
AA′	2	7.56	2.0, 8.0*
BB′	2	7.40	8.0, 8.0*
C	1	7.42	2.0, 8.0*
		**MolDeTr**	
AA′	2	7.56	1.8, 8.3
BB′	2	7.41	8.3, 9.3
C	1	7.41	0.8, 10.0

These results confirm MolDeTr's ability to accurately predict and deconvolute strongly coupled aromatic spin systems.

References

[1] Jacobsen, N. E. *NMR Data Interpretation Explained: Understanding 1D and 2D NMR Spectra of Organic Compounds and Natural Products* (John Wiley & Sons, 2016). Google-Books-ID: OxQWCgAAQBAJ.

[2] Jeannerat, D. & Cobas, C. Application of multiplet structure deconvolution to extract scalar coupling constants from 1d nuclear magnetic resonance spectra. *Magnetic Resonance* **2**, 545–555, DOI: 10.5194/mr-2-545-2021 (2021). Publisher: Copernicus GmbH.

[3] Laatikainen, R., Tiainen, M., Korhonen, S.-P. & Niemitz, M. Computerized analysis of high-resolution solution-state spectra. In *eMagRes*, DOI: 10.1002/9780470034590.emrstm1226 (John Wiley & Sons, Ltd, 2011). _eprint: https://onlinelibrary.wiley.com/doi/pdf/10.1002/9780470034590.emrstm1226.

[4] Dashti, H. *et al.* Spin system modeling of nuclear magnetic resonance spectra for applications in metabolomics and small molecule screening. *Analytical Chemistry* **89**, 12201–12208, DOI: 10.1021/acs.analchem.7b02884 (2017). Publisher: American Chemical Society.

[5] Schmid, N. *et al.* Deconvolution of 1d NMR spectra: A deep learning-based approach. *Journal of Magnetic Resonance* **347**, 107357, DOI: 10.1016/j.jmr.2022.107357 (2023).

[6] Li, D.-W., Hansen, A. L., Bruschweiler-Li, L., Yuan, C. & Brüschweiler, R. Fundamental and practical aspects of machine learning for the peak picking of biomolecular NMR spectra. *Journal of Biomolecular NMR* **76**, 49–57, DOI: 10.1007/s10858-022-00393-1 (2022).

[7] Li, D.-W., Hansen, A. L., Yuan, C., Bruschweiler-Li, L. & Brüschweiler, R. DEEP picker is a deep neural network for accurate deconvolution of complex two-dimensional NMR spectra. *Nature Communications* **12**, 5229, DOI: 10.1038/s41467-021-25496-5 (2021). Publisher: Nature Publishing Group.

[8] Fischetti, G. *et al.* A deep learning framework for multiplet splitting classification in 1H NMR. *Journal of Magnetic Resonance* **373**, 107851, DOI: 10.1016/j.jmr.2025.107851 (2025).

[9] Zhu, X. *et al.* Deformable DETR: Deformable transformers for end-to-end object detection. In *International Conference on Learning Representations (ICLR)*, DOI: 10.48550/arXiv.2010.04159 (2021). ArXiv:2010.04159.

[10] He, K., Zhang, X., Ren, S. & Sun, J. Deep residual learning for image recognition. In *2016 IEEE Conference on Computer Vision and Pattern Recognition (CVPR)*, 770–778, DOI: 10.1109/CVPR.2016.90 (2016). ISSN: 1063-6919.

[11] Chen, Q. *et al.* Group DETR: Fast DETR training with group-wise one-to-many assignment. In *Proceedings of the IEEE/CVF International Conference on Computer Vision (ICCV)*, 6610–6619, DOI: 10.1109/ICCV51070.2023.00610 (2023).

[12] Liu, L. *et al.* On the variance of the adaptive learning rate and beyond (2020).

[13] Kingma, D. P. & Ba, J. Adam: A method for stochastic optimization, DOI: 10.48550/arXiv.1412.6980 (2017). 1412.6980[cs].

[14] Carion, N. *et al.* End-to-end object detection with transformers. In Vedaldi, A., Bischof, H., Brox, T. & Frahm, J.-M. (eds.) *Computer Vision – ECCV 2020*, 213–229, DOI: 10.1007/978-3-030-58452-8_13 (Springer International Publishing, 2020). TLDR: This work presents a new method that views object detection as a direct set prediction problem, and demonstrates accuracy and run-time performance on par with the well-established and highly-optimized Faster RCNN baseline on the challenging COCO object detection dataset.

[15] Corsaro, C. *et al.* NMR in metabolomics: From conventional statistics to machine learning and neural network approaches. *Applied Sciences* **12**, 2824, DOI: 10.3390/app12062824 (2022). Number: 6 Publisher: Multidisciplinary Digital Publishing Institute.

[16] Debus, B., Parastar, H., Harrington, P. & Kirsanov, D. Deep learning in analytical chemistry. *TrAC Trends in Analytical Chemistry* **145**, 116459, DOI: 10.1016/j.trac.2021.116459 (2021).

[17] Hoffman, R. A., Forsén, S. & Gestblom, B. Instant analysis of NMR spectra. In Hoffman, R. A., Forsén, S. & Gestblom, B. (eds.) *Analysis of NMR Spectra*, 49–134, DOI: 10.1007/978-3-642-65205-9_4 (Springer, 1971).

[18] Reich, H. J. NMR resources (2024).

[19] Corio, P. L. The analysis of nuclear magnetic resonance spectra. *Chemical Reviews* **60**, 363–429, DOI: 10.1021/cr60206a003 (1960). Publisher: American Chemical Society.

[20] Ernst, R. R., Bodenhausen, G. & Wokaun, A. *Principles of Nuclear Magnetic Resonance in One and Two Dimensions* (Clarendon Press, 1987). Google-Books-ID: XndTnwEACAAJ.

[21] De Lorenzi, F. *et al.* Bayesian analysis of 1d 1h-NMR spectra. *Journal of Magnetic Resonance* **364**, 107723, DOI: 10.1016/j.jmr.2024.107723 (2024).

[22] van Meerten, S. G. J. SHIMpanzee: A program for the simulation of NMR shim lineshapes (2021).

[23] Huang, Z., Bi, T., Jiang, H. & Liu, H. Review on NMR as a tool to analyse natural products extract directly: Molecular structure elucidation and biological activity analysis. *Phytochemical Analysis* **35**, 5–16, DOI: 10.1002/pca.3292 (2024). TLDR: By these methods, molecular structure and biological activity information will be directly obtained from NMR analysis of natural products extract, aiming to save experimental time and expenses.

[24] Emwas, A.-H. *et al.* NMR spectroscopy for metabolomics research. *Metabolites* **9**, 123, DOI: 10.3390/metabo9070123 (2019). Number: 7 Publisher: Multidisciplinary Digital Publishing Institute.

[25] Markley, J. L. *et al.* The future of NMR-based metabolomics. *Current Opinion in Biotechnology* **43**, 34–40, DOI: 10.1016/j.copbio.2016.08.001 (2017).

[26] Emwas, A.-H. *et al.* NMR as a "gold standard" method in drug design and discovery. *Molecules* **25**, 4597, DOI: 10.3390/molecules25204597 (2020). Number: 20 Publisher: Multidisciplinary Digital Publishing Institute.

[27] Renzi, P., Hioe, J. & Gschwind, R. M. Enamine/dienamine and brønsted acid catalysis: Elusive intermediates, reaction mechanisms, and stereoinduction modes based on in situ NMR spectroscopy and computational

studies. *Accounts of Chemical Research* **50**, 2936–2948, DOI: 10.1021/acs.accounts.7b00320 (2017). Publisher: American Chemical Society.

[28] Holzgrabe, U. & Malet-Martino, M. Analytical challenges in drug counterfeiting and falsification—the NMR approach. *Journal of Pharmaceutical and Biomedical Analysis* **55**, 679–687, DOI: 10.1016/j.jpba.2010.12.017 (2011).
